# Motif and conserved module analysis in DNA (promoters, enhancers) and RNA (lncRNA, mRNA) using AlModules

**DOI:** 10.1038/s41598-022-21732-0

**Published:** 2022-10-20

**Authors:** Muharrem Aydinli, Chunguang Liang, Thomas Dandekar

**Affiliations:** Department of Bioinformatics, Biocenter, University of Würzburg, Am Hubland, 97074 Würzburg, Germany

**Keywords:** Computational biology and bioinformatics, Evolution

## Abstract

Nucleic acid motifs consist of conserved and variable nucleotide regions. For functional action, several motifs are combined to modules. The tool AIModules allows identification of such motifs including combinations of them and conservation in several nucleic acid stretches. AIModules recognizes conserved motifs and combinations of motifs (modules) allowing a number of interesting biological applications such as analysis of promoter and transcription factor binding sites (TFBS), identification of conserved modules shared between several gene families, e.g. promoter regions, but also analysis of shared and conserved other DNA motifs such as enhancers and silencers, in mRNA (motifs or regulatory elements e.g. for polyadenylation) and lncRNAs. The tool AIModules presented here is an integrated solution for motif analysis, offered as a Web service as well as downloadable software. Several nucleotide sequences are queried for TFBSs using predefined matrices from the JASPAR DB or by using one’s own matrices for diverse types of DNA or RNA motif discovery. Furthermore, AIModules can find TFBSs common to two or more sequences. Demanding high or low conservation, AIModules outperforms other solutions in speed and finds more modules (specific combinations of TFBS) than alternative available software. The application also searches RNA motifs such as polyadenylation site or RNA–protein binding motifs as well as DNA motifs such as enhancers as well as user-specified motif combinations (https://bioinfo-wuerz.de/aimodules/; alternative entry pages: https://aimodules.heinzelab.de or https://www.biozentrum.uni-wuerzburg.de/bioinfo/computing/aimodules). The application is free and open source whether used online, on-site, or locally.

## Introduction

Nucleic acid motifs are fascinating, composed of conserved as well as variable nucleotide regions. Several motifs often combine to modules. Our new software AIModules identifies nucleic acid motifs as well as combinations of these. It looks at conservation comparing several nucleic acid stretches. Our software allows analysis of promoter and transcription factor binding sites (TFBS), but also it identifies conserved modules (motif combinations) shared between several gene families. Biological applications include the study of promoter regions and the analysis of shared and conserved other DNA motifs such as enhancers and silencers. However, also RNA can be analyzed for motifs and combinations of them for instance mRNA for regulatory elements such as regulation of polyadenylation as well as lncRNAs for motifs and lncRNA-specific modules.

We present a free and open-source tool which offers basic and stand-alone analysis of promoter regions including individual transcription factor binding sites (TFBS), analysis of TFBS combinations (modules) and the option to compare partial or full conservation of individual TFBS and complete modules looking at a number of promoter regions at the same time upon which a longer, more detailed and specific evaluation can be built. Different biological use cases (e.g. motif and module search) are presented and show also the more general analysis options for DNA and RNA. We generalized our tool so that other DNA motifs can be searched as well as more complex combinations of such. Moreover, RNA motifs and conserved bindings sites in RNA can also be efficiently searched.

With its functionalities for identification of conserved motifs and conserved modules in nucleic acids, we fill a gap between more sophisticated commercial tools and analysis packages and direct motif discovery tools and databases. Transcription factors can be found in databases such as GenBank or Uniprot, and their binding sites can also be found in other resources such as specific promoter region databases (e.g. Prodoric for prokaryotes), or gene regulatory element databases (e.g. MotifMap), etc. In addition, the combination of both binding site and corresponding transcription factor is provided in some databases, such as TRANSFAC®. TRANSFAC® offers a publicly free but outdated version from the year 2005 of its binding sites for download, and a free web-service for the new release of version 2021.3. A download of the updated database is only available commercially. However, our aim here is the identification of conserved nucleic acid motifs. In promoter analysis this would be the specific task to spot conserved TFs and TFBSs shared between several promoter sequences. There are companies such as *Genomatix* (currently owned by Intrexon Bioinformatics Germany GmbH) which offer this functionality as commercial software as well as a detailed promoter analysis including expert evaluation of gene expression changes and several further software suites. Similarly, an academic institution would do an in-depth analysis for a promoter region or transcriptome analysis where the conserved promoter, TFBS or module search is only one item of a large-scale study. However, to offer something similarly broad and intensive as this service is not the intention of our work.

For our queries, we employ a user-friendly ready-made collection of matrices for promoter and DNA element analysis: JASPAR. The up-to-date JASPAR^1^ database uses high quality matrices generated from SELEX, protein binding microarray (PBM), ChiP-based assays, etc.^2^ (see Supplement Evaluation section “Matrix Generation”). Via these experimental techniques, TFs and their binding sites can be identified with high confidence. However, bioinformatical searches relying only on the DNA motif (the nucleotide sequence of the binding site) for predicting a TFBS besides predicting the correct binding site often produce false positive results (overprediction), for example due to not considering TF-TF protein interactions in the preinitiation complex or other details during the DNA annealing of the TF. A good strategy to avoid such overpredictions is to search for conserved TFBSs in related promoter regions restricted to a gene family either within one species or shared between species. Even better specificity (correctly predicting the true binding sites) is achieved by considering conserved combinations of TFBS, so called modules. There are cell type specific modules, such as TFBS combinations specific for a liver cell, as well as functional modules, e.g. an immunoglobulin promoter.

Hence, to elucidate gene expression and its regulation better, including conservation of TFBSs and TFBS combinations (modules) in related gene families within a species or shared between species, our tool AIModules was created.

## Results

### Motif searches in nucleic acids

There are several programs that find modules, defined as a combination of motifs in nucleic acid sequences. In particular, within the MEME^3,4^ suite, both MAST^5^ and MCAST^6^ can find such modules in the nucleic acid stretch of choice. The software is both free and on a website.

However, a careful test of how far the motifs and modules are conserved over or shared between several nucleic acid stretches is not involved but allows further insights: By this comparison, it is possible to find conserved motifs and even conserved modules shared between genes, or shared between several RNAs or several nucleic acid stretches of choice. A suitable software for this should only be marking (i) conserved motifs in the nucleic acids compared and subsequently mark and show (ii) conserved modules.

Therefore, we sought to make an open-source tool available that allows the user to insert their chosen DNA stretches and matrices, but also provides a comprehensive collection of matrices from JASPAR. The AIModules tool is a web service which allows easy inclusion and application of new features, e.g. inclusion of the TRANSFAC® database. To decrease the burden on our servers, only the motif search is conducted there. The calculations for the module discovery are then performed automatically in the browser of the user’s computer.

The User Interface is designed to support the user, so that a quick start is possible: the user sequence is given in FASTA format and checked for the presence of nucleotide characters only, and then the submit button appears (see Supplement Tutorial file for detailed instructions). Progress is indicated by a rotating wheel close by. Alternatively, the user can first hit any of the demo buttons to see the results AIModules delivers for different use cases.

**Figure 1:**
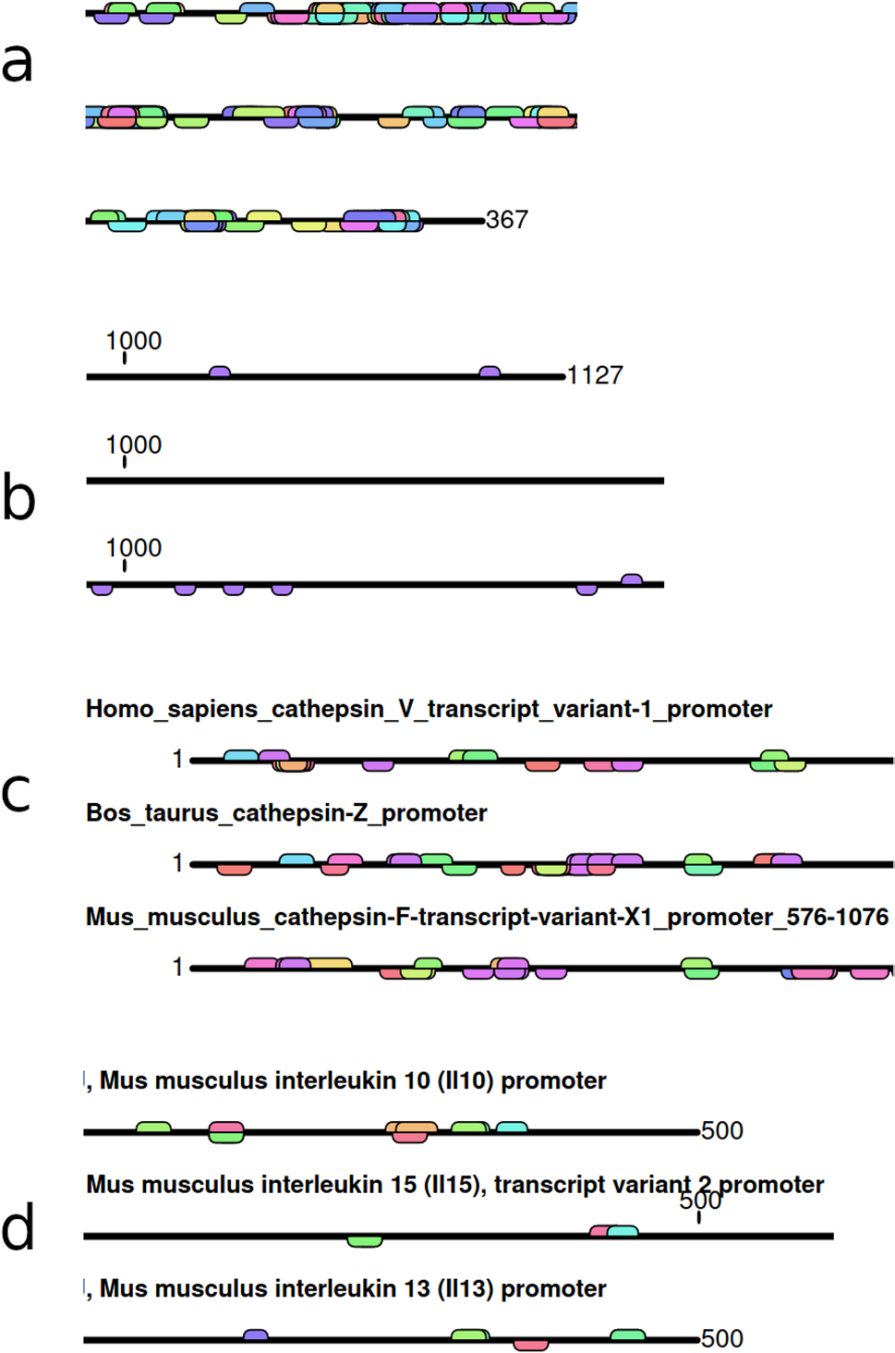
Transcription factor binding and module searches using AIModules. Subfigures (**a**–**d**) are only excerpts; for full results visit https://bioinfo-wuerz.de/aimodules/; the black line represents the sequence itself. Above the line you will find the motifs of the (+) strand and below the line are the motifs from the (−) strand. The results can be downloaded from the web application as an Excel file; the application is described in detail in the Supplementary Tutorial file; the sequences can be found in the Supplement Evaluation section “Used sequences”; (**a**) TFBS search for the promoters X73536.1_H.sapiens_promoter_region_of_human_IL-10_gene, AY486432.1_Macaca-mulatta_ interleukin-10-(IL-10)_gene_promoter_region and AF121965.1_Mus-musculus_interleukin-10-(IL10)_gene_promoter_partial_sequence (configuration: La 6, Ld 8, vertebrates matrices from JASPAR DB) (excerpt contains base pairs 250–400; results can be found in the file Supplements.zip); (**b**) Poly adenylation site motif search for the promoters NM_000600.5 Homo sapiens interleukin 6 (IL6)-transcript variant 1, NM_000594.4 Homo sapiens tumor necrosis factor (TNF) and NM_020525.5 Homo sapiens interleukin 22 (configuration: La 9, Ld 8, own Poly-(A)-matrix) (excerpt contains base pairs 990–1150; results can also be found in the Supplement Evaluation section “Poly adenylation site motif results”); (**c**) Module search for promoters Homo_sapiens_cathepsin_V, Bos_taurus_cathepsin-Z and Mus_musculus_cathepsin-F (configuration: threshold 3, La 6, Ld 8, vertebrates matrices from JASPAR DB) (excerpt contains base pairs 1–200; results can also be found in the file Supplements.zip); (**d**) Module search for promoters Mus musculus interleukin 10, Mus musculus interleukin 15 and Mus musculus interleukin 13 (configuration: threshold 3, La 8, Ld 8, vertebrates matrices from JASPAR DB) (excerpt contains base pairs 300-550; NFAT for all sequences was found as previously described in^35^; results can also be found in the Supplement Evaluation Table S10).

After module calculation, the result for the module search is depicted visually and can be downloaded as an Excel file (Fig. 1; for details see Supplementary Tutorial file).

AIModules offers three biological applications for motif or module searches: TFBS motif searches in promoter regions, motif and module searches in other DNA regions and finally RNA motif and module search.

### Motif search in a promoter region

This is a basic search AIModules offers. As far as we know, for exactly this application, only two commercial products (Genomatix’s MatInspector as well as ModelInspector and TRANSFAC® database) were available which could compare and highlight modules comparing different promoter regions.

For this biological application, the AIModules user inserts DNA sequences for analysis, selects the thresholds (La and Ld) and the matrices. These can be selected from the database or individual ones can be inserted. Matrices for AIModules can also be generated in the tool itself. An example on the TFBS search is depicted in Fig. 1a. This basic search is explained in the file “Supplement-Tutorial” as Use case 1.1.; a typical output is shown (Figure S1 of Supplement Tutorial) while the second part of the file takes the user step by step through the program functionalities starting from this use case.

### Motif search in an enhancer region

The functionalities of AIModules can also be used to analyze any other DNA stretch of choice or interest in a comparative way. This can be useful for any motif or module reported by the ENCODE consortium^7,8^. This is a powerful application of AIModules where there are not yet many tools available and is illustrated for the comparative analysis of two enhancer regions. By this, one can easily determine which conserved motifs are used by all putative enhancer regions compared and even different modules if present, can be rapidly determined. AIModules works quickly and is easy to use, but the user must have sufficient knowledge about the biology of the DNA sequences analyzed to properly interpret the result. Furthermore, TFBSs in enhancer regions have complex effects (changing gene activity in genes thousands of nucleotides away) and so the analysis is complex, difficult and needs validation experiments. However, an initial analysis applying AIModules allows one to rapidly see which TFBSs of potential interest are there and equally importantly, which though assumed to be there are missing. To demonstrate this, putative enhancer regions in two virus sequences were examined.

For finding similar enhancer regions we used the sequences of Simian virus 40 partial genomic sequence, strain 4A (AJ276576.1 GI:7339596) and Human herpesvirus 4 complete wild type genome (AJ507799.2 GI:86261677), and searched for the motifs CREB, Myb and p53. The motif analysis however showed 21 Myb, 1 CREB, and no p53 motifs for full length AJ276576.1. The sequence contains a 72 bp tandem repeat enhancer sequence (17.0.88) which contains a TFBS for MA1426.1 MYB124 (31…40).

On the other hand, the sequence from AJ507799.2 contains 30 Myb, 7 CREB and again no p53 motifs. There is a cell type specific enhancer of that sequence (7421.0.8042) and none of the above three TFBSs are present. A conserved module search of the enhancer sequences of both species did not result in any modules, stressing their differences (La = 8, Ld = 7).

The analysis shows that AIModules can be used to rapidly get an overview of which transcription factors bind in two putative enhancer regions, how far they share similarities (including conserved modules) and, as shown here, which assumed transcription factors are found after analysis to be conspicuously missing (i.e. p53 TFBS).

### RNA motif search

AIModules also enables analysis of RNA motifs and combinations of such motifs. The RNA sequence of choice can be analyzed by the user. Uracils are internally converted by our algorithm to thymine. After selecting the parameters La and Ld as well as selecting the matrix, the result is obtained. La is the actual log-odds ratio of the match, whereas Lm is the maximum possible log-odds ratio for a match, i.e. the information content of the consensus sequence. Ld on the other hand is the maximum log likelihood deficit (Lm–La)—or put simply La can be understood as the lower and Ld as the top score threshold between which a TFBS is valid. The score is calculated via position weight matrices (PWMs)^9,10^. The user may select JASPAR matrices from a drop-down menu within the web application or insert their own. We did not include the TRANSFAC® database as the download of the latest version is only available commercially; however, this alternative can easily be uploaded using older public versions^11^. Similarly, an in-house developed search matrix or different matrix database of choice (with correct format) may be uploaded and used.

To illustrate the capabilities for RNA searches, we give the example of how to look for poly adenylation site motifs. These direct the polyadenylation machinery during mRNA maturation so that polyadenylation is affected here and not at another point. Hence, screening for such motifs is helpful both in genomic sequences as well as in mRNAs; this goes beyond simply looking for the long poly-(A) tail. A suitable RNA motif search matrix of twelve poly adenylation site motifs from^12^ was generated in AIModules and used for the analysis. A result using our A-rich motif matrix (including the well-known hexamer motifs AATAAA or AAAAAA) is depicted in Fig. 1b. The matches on the antisense strand apply only to DNA. The result can also be downloaded as an Excel table (see Table 1).

**Table 1:** Results of the poly adenylation site motif search.

Sequence Name	Sequence Length	Matrix Length	Hit Sense	Hit Start	Hit Stop	Hit Score (La)	Hit Max log likelihood ratio score (Lm) or matrix possible	Difference (Ld) (maxscore(Lm)—score(La))	Hit Oligo
**NM_000600.5 Homo sapiens interleukin 6 (IL6), transcript variant 1, mRNA**	1127	6	N	799	804	7.002311	9.889837	2.887525	ACTAAA
			N	1025	1030	9.889837	9.889837	0.000000	AATAAA
			N	1054	1059	6.854213	9.889837	3.035624	AAGAAA
			N	1084	1089	7.002311	9.889837	2.887525	TATAAA
			N	1103	1108	9.889837	9.889837	0.000000	AATAAA
			N	1118	1123	6.854213	9.889837	3.035624	AAAAAA
			R	827	832	7.002311	9.889837	2.887525	TTTAAT
			R	837	842	7.002311	9.889837	2.887525	TTTAAT
			R	842	847	9.889837	9.889837	0.000000	TTTATT
			R	876	881	7.002311	9.889837	2.887525	TTTATG
			R	891	896	7.002311	9.889837	2.887525	TTTATA
			R	893	898	7.002311	9.889837	2.887525	TATATT
			R	923	928	7.002311	9.889837	2.887525	TTTATG
			R	927	932	7.002311	9.889837	2.887525	TGTATT
			R	998	1003	6.854213	9.889837	3.035624	TTTCTT
			R	1062	1067	7.002311	9.889837	2.887525	TTTATA
			R	1064	1069	7.002311	9.889837	2.887525	TATATT
			R	1069	1074	7.002311	9.889837	2.887525	TGTATT
			R	1073	1078	7.002311	9.889837	2.887525	TTTATA
			R	1095	1100	7.002311	9.889837	2.887525	TTTATA
**NM_000594.4 Homo sapiens tumor necrosis factor (TNF), mRNA**	1678	6	N	1655	1660	9.889837	9.889837	0.000000	AATAAA
			N	1673	1678	6.854213	9.889837	3.035624	AAGAAA
			R	928	933	9.889837	9.889837	0.000000	TTTATT
			R	1319	1324	7.002311	9.889837	2.887525	TCTATT
			R	1323	1328	7.002311	9.889837	2.887525	TTTATG
			R	1345	1350	9.889837	9.889837	0.000000	TTTATT
			R	1352	1357	9.889837	9.889837	0.000000	TTTATT
			R	1356	1361	9.889837	9.889837	0.000000	TTTATT
			R	1363	1368	9.889837	9.889837	0.000000	TTTATT
			R	1367	1372	9.889837	9.889837	0.000000	TTTATT
			R	1383	1388	7.002311	9.889837	2.887525	TGTATT
			R	1387	1392	9.889837	9.889837	0.000000	TTTATT
			R	1526	1531	6.854213	9.889837	3.035624	TTTTTT
			R	1538	1543	7.002311	9.889837	2.887525	TTTATC
**NM_020525.5 Homo sapiens interleukin 22 (IL22), mRNA**	1165	6	N	900	905	7.002311	9.889837	2.887525	CATAAA
			N	903	908	6.854213	9.889837	3.035624	AAAAAA
			N	933	938	6.854213	9.889837	3.035624	AAAAAA
			N	981	986	7.002311	9.889837	2.887525	TATAAA
			N	1144	1149	9.889837	9.889837	0.000000	AATAAA
			R	579	584	7.002311	9.889837	2.887525	TTTATG
			R	683	688	6.854213	9.889837	3.035624	TTTTTT
			R	684	689	6.854213	9.889837	3.035624	TTTTTT
			R	685	690	6.854213	9.889837	3.035624	TTTTTT
			R	786	791	7.002311	9.889837	2.887525	TTTATA
			R	824	829	9.889837	9.889837	0.000000	TTTATT
			R	873	878	9.889837	9.889837	0.000000	TTTATT
			R	877	882	6.854213	9.889837	3.035624	TTTTTT
			R	973	978	7.002311	9.889837	2.887525	TTTATA
			R	975	980	7.002311	9.889837	2.887525	TATATT
			R	979	984	7.002311	9.889837	2.887525	TTTATA
			R	987	992	7.002311	9.889837	2.887525	TGTATT
			R	991	996	9.889837	9.889837	0.000000	TTTATT
			R	1015	1020	9.889837	9.889837	0.000000	TTTATT
			R	1019	1024	7.002311	9.889837	2.887525	TTTATA
			R	1029	1034	9.889837	9.889837	0.000000	TTTATT
			R	1043	1048	9.889837	9.889837	0.000000	TTTATT
			R	1047	1052	7.002311	9.889837	2.887525	TTTATA
			R	1100	1105	7.002311	9.889837	2.887525	TTTATG
			R	1131	1136	9.889837	9.889837	0.000000	TTTATT

AIModules allows a search for polyadenylation site motifs. Generally, with AIModules one may search for functional RNA sites as well. The matrix was generated from twelve consensus sequences. Matches on the antisense strand (R) apply only to DNA.

The table is truncated. The full list of analyzed mRNAs can be found in Supplementary Evaluation section “Used sequences” and the complete table of poly adenylation site motif results in Supplementary Evaluation section “Poly adenylation site motif search.”

If we instead wanted to look for a protein cleavage site or even a long stretch of As, the motif matrix must be changed accordingly. One important application area in which to look for such conserved motifs (or even motif combinations comparing a family of RNAs or conserved RNA from several species) is regarding RNA–protein binding motifs such as the Sm-site in splicing RNAs. If a suitable matrix is prepared, AIModules can look for such motifs easily. Similarly, a full 3´UTR machinery of several proteins binding to the 3´UTR for governing polyadenylation or determining mRNA stability can be examined in detail and compared, detecting modules of similar protein binding sites used to control polyadenylation or mRNA stability.

Another potentially even more important application of AIModules is the detection of motifs or motif combinations conserved in lncRNAs such as CHAST lncRNA^13^. Here our algorithm allows a similar strategy as in promoter module search: by comparison of related lncRNAs, the truly conserved motifs and their modules are revealed. However, this requires an intensive, dedicated study of a lncRNA family not pursued here.

### Module search

For the module search, the user may insert their own sequences, select the parameters for La and Ld, and activate the checkbox for module filtering. The conservation of the TF can be selected by the user using a stepper menu. The user may then select either JASPAR matrices or insert their own matrices as well. Results from module searches are shown in Fig. 1c and d.

The promoter sequences we used can be found in Supplement Evaluation section “Used sequences.” Resulting TFBSs and modules can be downloaded as Excel files from the web application; then the user may select and deselect TFs from the result in order to manually search for modules (see Supplementary Tutorial file).

This conserved module search nicely illustrates the strong performance of our tool.

In the file “Supplement-Tutorial” the user is prepared step-by-step for this task by doing Use case 1, starting from individual transcription factor binding sites (Use cases 1.1 and 1.2) to conserved modules which are cell- or function-specific (Use cases 1.3 and 1.4, respectively) and then performing conserved module search (Use case 1.5) and full promoter module analysis (Use case 1.6).

Our module search concentrates on common transcription factor binding sites of the inserted sequences and shows common modules in the next step. These consist of two transcription factors which contain an offset of + /− 200 bp and are shared in at least two of the inserted sequences. For more details on this see the Discussion section.

Our approach for both transcription factor binding site and module search is generic and thus our tool can also be used for RNA sequences (mRNA, lncRNA) as well as ENCODE motifs^7,8^ enhancer, silencer, telomeres, complex regulatory elements in DNA).

From these functionalities, three main biological use cases are detailed in the tutorial supplemental file:

#### Promoter motifs including TFBSs and combinations (modules)

This can start from (i) identification of the conserved binding site of a specific TF or the specific TFBS in a promoter region. The TF motif search can also be adapted as there is more variation recognized e.g. by more experimental data; (ii) identify conserved TF binding sites comparing several genes in a gene family either shared between species or occurring within a species, looking for a conserved promoter module in a gene family, finding a cell-type specific module, e.g. a liver-specific module and finally finding a *functional* module, for instance an immunoglobulin module.

#### RNA motifs and motif combinations

Here applications can range from a polyadenylation site motif (combination) to RNA binding motifs conserved in a gene family or over the same RNA in different species to identification of conserved localization signals (for instance an oskar-mRNA localization motif). This tool should also help better characterize combined motifs in lncRNAs. Such lncRNAs are identified at an increasing pace, but to find out which are functional and if so, which specific function they have, heavily depends on recognition of the involved and conserved motifs; AIModules supports identification of modules shared between lncRNAs in related species or a multicopy lncRNA family within a species. The regulatory lncRNA CHAST in cardiomyocytes is a nice demonstration example^13^.

#### Non-promoter DNA motifs

These can be silencers and enhancers, but also any other conserved DNA motif or module of interest such as telomeres and structural DNA regions, or the numerous motifs and motif combinations discovered by the ENCODE consortium^7,8^. In fact, the ability of our tool to recognize conserved combined motifs denoting an enhancer region should improve the non-promoter motif detection. However, to systematically investigate this is a study of its own and not attempted here; instead we provide a free tool to support this fascinating use case.

### Performance comparison to other tools (motif search)

We compared AIModules to the web application conTraV3^14^ and to Softberry NSite^15–17^ regarding testing TFBS search as offered by the two alternative tools. We analyzed one sequence (AJ223836.1 *Chionodraco hamatus* mRNA for cathepsin; length 1332 bp; for sequence see Supplement Evaluation section “conTraV3 comparison”). For AIModules we chose all 1920 matrices from Jaspar 2022 with the parameters *La* = *7* and *Ld* = *9*, which resulted in 608 TFBSs and 1390 motifs. The analysis took 0.6 s on the server and 4.5 s for rendering on the webpage. For conTraV3 only a maximum of *20 matrices* could be selected, so we picked matrices from *jaspar_2016_core* with the parameters *core* = *0.95* and *similarity matrix* = *0.85.* After 16 s, 18 TFBs and 55 motifs were identified. Next, we performed an analysis on Softberry NSite, where we picked the ooTFD set of TFBSs (8030 non-redundant Human/Animal Transcription Regulatory Elements) and selected a *Statistical Significance Level of 0.95*. After ca. 9 s, 26 different TFBSs and 58 motifs were detected (see Table 2). We want to clarify that AIModules depicts 2 time values—one describes the time passed for the calculations on the server and the other one for calculations on the computer/ in the browser (rendering). The observed run times are only an indication for the user-friendliness of the tool; much faster comparisons are possible using computer processors directly.

**Table 2:** Tool comparison: Performance Statistics for found motifs and common TFBSs.

Product	Time for analyses [sec]	Number of matrices	Found motifs	Common TFBSs
*AIModules*	0.6 for response + 4.5 for rendering	1920	1390	18
*conTraV3*	16	20	55	18
*Softberry NSite*	9	8030	58	4

Compared to conTraV3 and Softberry NSite, AIModules finds many more motifs in a shorter time frame. We found that all products share common motifs. Further details and the selected matrices for contraV3 may be found in the file *contrav3_vs_aimodules_vs_softberry.xls* within the archive *Data-Supplements.zip.*

### Comparison to commercial products (TFBS and Module search)

To analyze how well our solution performs, we took our results and compared them to those from the company Genomatix, using MatInspector and ModelInspector for motif and module discovery, respectively^8^, by looking at an example set of selected genes. Genomatix’s solution is a commercially available tool with a one-week free trial period (available tools in the field of promoter analysis are found in the *Discussion* section). To produce results from the Genomatix solutions, we used the one-week free trial version. The homologous promoters are taken from GenBank and in the first example are cathepsins (Homo_sapiens_cathepsin_V_transcript_variant-1_promoter, Bos_taurus_cathepsin-Z_promoter, Mus_musculus_cathepsin-F-transcript-variant-X1_promoter_576-1076); the second example is IL-10 (X73536.1_H.sapiens_promoter_region_of_human_IL-10_gene, AY486432.1_Macaca-mulatta_interleukin-10-(IL-10)_gene_promoter_region, AF121965.1_Mus-musculus_interleukin-10-(IL10)_gene_promoter_partial_sequence). The sequences are depicted in Supplement Evaluation section “Used sequences.”

The resulting modules from AIModules and ModelInspector were compared by direct inspection, as the modules from ModelInspector were low in number. We found that the results from a crude TFBS search were handled differently. Due to the high number of the findings and the differences between the naming of the TFBSs from both systems, we started with an automatic approach and only directly inspected by eye the processed output on the spreadsheet created. Each result set was put into python arrays and the ModelInspector result sets were copied unchanged into another array. The corresponding arrays from AIModules and ModelInspector were then compared for string equality by a python script (see Supplements.zip). The matches were put into separate arrays with the syntax *AIModules_TFBS_name::Genomatix_TFBS_name,* where *Genomatix_TFBS_name* can consist of multiple hits which are separated by commas. The resulting arrays were then printed to *standard output* and refined manually in *libreoffice Spreadsheet*. The parameters used for both solutions and the statistics of the found TFBSs and modules are depicted in Supplement Evaluation Tables S1–S8.

We observed that AIModules found more TFBSs than Genomatix’s MatInspector (e.g. 253 vs. 155, see Supplement Evaluation Tables S1–S2) and that some motifs are common to both systems (e.g. 15, see Supplement Evaluation Table S2 and S4). Regarding modules, AIModules found ten-fold more modules than Genomatix’s ModelInspector (e.g. 486 vs. 15, see Supplement Evaluation Table S6 and S8).

The amount of found TFBSs from both methods differ in number. This is due to differences in available matrices and the setting of search parameters which are for AIModules La and Ld, and for the Genomatix’s MatInspector 0.75 and *Optimized*. As explained in methods, we give considerations how these parameters can be compared and where they differ. Regarding the module search, the differences between the system parameters are similar to the ones for TFBSs. In AIModules the parameters are La, Ld and the activated checkbox for module search, whereas Genomatix’ ModelInspector uses a *Threshold for number of elements* and a *Maximum number of matches*.

As these parameters are difficult to directly compare and normalize to each other, the found matches have only a small overlap. Additionally, some of the TFBSs are unique to the system used.

We show that our tool is the only one that can detect common modules within the analyzed sequences. Moreover, we combine this feature with a TFBS search as well as RNA motif discovery. Our tool allows the user to insert not only their own sequences but also their own matrices.

Each of the motif discovery tools mentioned in Tables 2 and 3 are the results of meticulous work and they have their own use cases. For the uses specified, however, AIModules has demonstrated its ability to find more modules than even a commercial product. A detailed comparison of the tools from Tables 2 and 3 can be found in the supplementary file “supplement evaluation” with the section “Comparison of the different tools for TFBS discovery.”

**Table 3:** Comparison of promoter analysis tools.

Name	Search for TFBSs	Saved in Databases	Precalculated results	Insert user´s own matrices	Insert user sequences	Show common modules of user sequences
*AIModules*	Yes	Matrices	No	Yes	Yes	Yes
*ContraV3*^14^	Yes	Genomes and Matrices	No	Yes	Yes	No
*TRANSFAC®*^36, 37^ *(free version and commercial version)*	Yes	Genomes, promoters, matrices and modules	No	Yes	Yes	No; modules are calculated for each sequence
*Genomatix*^38^ (MatInspector and ModelInspector; commercial)	Yes	Genomes, promoters, matrices and modules (vertebrates and plants)	No	Yes	Yes	No; for each sequence user modules are depicted (in the free trial version examined)
*Motifmap*^39–41^	Yes	Genomes and Matrices	No	No	No	No
*Promo*^42, 43^	Yes	Matrices	No	No	Yes	No, only common TFBSs
*ModuleMaster*^21^	Could not be run under Linux Mint 64bit and Windows 10 64bit. Not supported anymore, as the originating Lab does not do bioinformatics anymore					
*Prodoric*^44, 45^	Yes	Genomes and Matrices	Yes	No	Yes, but only one	No

**Table 4:** Comparison of promoter analysis tools: Further tools.

Name	Search for TFBSs	Saved in Databases	Precalculated results	Insert user matrices	Insert user sequences	Show common modules of user sequences
*Softberry*^16, 17, 46^	Yes	Matrices	No	No	Yes	No; only one sequence can be input
*TAIR*^47^	Yes; if TFBS is common to at least 3 sequences	Matrices	No	No	Yes	No; shows TFBSs common to at least 3 sequences; only for some plants; only for 6-mer TFBSs
*PlantPan 3.0*^48^	Yes	Genomes	No	No; but consensus sequence as IUPAC code	Yes	No; only common TFBSs
*CisBP*^29, 49^	Yes	Genomes and Matrices	No	No	Yes	No
*UniPROBE*^30, 50^	Yes	Matrices	No	No	Yes	No
*HOCOMOCO*^51, 52^	Yes	Matrices	No	No	Yes	No
*FlyFactorSurvey* ^53^	Yes	Genomes and Matrices	No	No	No	No
*MEME Suite*^54, 4^	Yes	Matrices	No	Yes	Yes	No
*YeTFaSCo*^55, 56^	Yes	Matrices	No	No	Yes	No
*TESS*^9, 34, 10^	Not available any more as a web service. The code of parts of the back-end is available					

## Discussion

### Broad biological applications

AIModules allows efficient module searches on more than one sequence and filters the common modules to render them clearly visible and distinguishable on the website. The application has many biological applications, allowing motif and conserved module analysis in DNA (e.g. promoters, enhancers) and RNA (e.g. lncRNA, mRNA). Direct motif identification is possible using prepared matrices (JASPAR database) as well as new, user generated matrices. Motif combinations (modules) can also be easily found using AIModules. Critical to avoiding overpredictions of motif binding sites (e.g. TFBS) or modules is the easy-to-use comparison of several nucleic acid sequences by AIModules. Using our algorithm, we can directly compare several promoter regions. This is a well-known and popular application where several different alternative programs are currently available with specific differences and limitations in their specific functionalities. An important advantage of AIModules is its flexibility in allowing the investigation of motifs in RNA and other DNA regions with similar efficiency. Regarding RNA, we show this in our example for mRNA/polyadenylation motifs, but this can also be applied to study motifs and motif combinations in lncRNAs. For DNA we depicted the investigation of putative enhancer regions but much more is possible; for example, AIModules could be applied to a detailed analysis of conservation and differences in repetitive DNA regions, where unique TFBSs and specific or conserved modules are highlighted by comparing several regions.

### Evaluation of application and algorithm

We have prepared the application in a way that allows for extensions without much effort, not only due to the architecture but also due to the chosen free and open-source licensing agreement (GPLv2). Moreover, the application is provided on our own server so that the user does not have to use complicated scripts or even commercial software.

Module analysis in AIModules follows a strict algorithm to the search for shared modules between the input sequences. For example, the TFBSs must have a match in *N* input sequences to be valid and hence be included in the module search. The number *N* can be defined by the user via a stepper control and defines the conservation of the TF. The strand orientation for TFBSs is relevant in this step. A module consists of two TFBSs with a fixed offset of + /− 200 bp. Every permutation of every TFBS is tested for validity (AB, AC, AD, …, BC, BD, …). The module is considered valid when it is shared between at least two input sequences.

### Comparison to alternative software

In Genomatix’s ModelInspector, all sequences are analyzed for known modules independently, i.e. ModelInspector does not show common modules. These differences in the module finding process lead to different numbers of modules found. Where AIModules finds all possible modules algorithmically, ModelInspector relies on known co-citations. This means that AIModules may over-represent modules, whereas ModelInspector only shows co-citations and may miss modules that are included in AIModules. However, the number of modules found can be refined in AIModules by increasing La, decreasing Ld, only using user input matrices, or a combination of these. The analyzed sequences for cathepsin and IL-10 showed no overlap in AIModules and Genomatix’s ModelInspector regarding modules. For each of these systems in silico search will not make experimental validation obsolete.

The other commercial product TRANSFAC® is available as a free version after registration. However, the matrices are from 2005, hence outdated, and limited in number (398 matrices). Furthermore, this free version is functionally constrained^18^ and the professional version is only available after licensing. AIModules offers 1920 matrices from the JASPAR DB, whereas the public version of TRANSFAC® contains only 398. Therefore, we chose the more up-to-date and sensitive matrices from the JASPAR DB, which also provides a REST-API and the JASPAR R/Bioconductor package^1^. Additionally, the matrices from TRANSFAC® cannot be downloaded, but must extracted from the website manually, which is time consuming as well as error prone. Since the AIModules application is open source, TRANSFAC® matrices can be added when needed.

It is also possible to register to TRANSFAC® geneXplain platform with a basic account. Via this account and the Composite Module Analyst^19^, modules within functionally related genes can be found. Furthermore, a one-week evaluation period may be obtained^20^ to test the full functionality of the platform (e.g. the MATCH Suite identifies TFs by orthologous and paralogous extension as well as tissue specificity).

Compared to those two most popular databases (Genomatix’s tools and TRANSFAC® public), AIModules offers the possibility to find far more patterns. AIModules can search multiple sequences and obtain comprehensive visualization and statistical results. AIModules also allows the user to select and deselect each of the found TFBSs and assemble TF patterns manually (for more see Supplementary Tutorial file).

By the time this manuscript is revised (16th September 2022) only two products were on the market that could predict modules. These tools are Genomatix’s ModelInspector (from Intrexon Bioinformatics Germany GmbH) and TRANSFAC®. ModuleMaster^21^ is another tool that could predict modules, but we were unable to start the WebStart Application on different operating systems. The lab of the authors of ModuleMaster could not provide any assistance as this bioinformatics research is no longer pursued. Furthermore, for the end user it is easier to use a website than a Java WebStart application that is not up to date, and which may pose a security hazard without a valid certificate. All the other tools in Tables 3 and 4 had no common module search functionality, but rather have their own unique and valuable uses. Moreover, AIModules is not only available as a web application, but can be deployed on an on-site server or locally on a PC or notebook as well.

Tables 3 and 4 are discussed in more detail in Supplement Evaluation section “Comparison of the different tools for TFBS discovery”.

Furthermore, for TFBS analyses R packages from Bioconductor^22^ are available (e.g. TFBSTools^23^, RcisTarget^24^, enrichTF^25^). These, however, must be packaged into new code before use on sequences for TFBS identification, in particular if you want to determine conserved TFBSs between different promoter regions and DNA sequences, or to establish a web server and visualization of TFBSs found.

Compared to the tools conTraV3 and Softberry NSite, we have shown that our tool outperforms those with regard to the time needed for the analyses as well as the number of found motifs (see Table 2). Additionally, we have shown that AIModules is able to detect polyadenylation sites (see Table 1) which were previously described in^10,12^. AIModules is not only faster but also presents features such as module search and RNA motif discovery, where the sequences as well as the matrices can be inserted by the user individually if desired. An overview of tools and features can be found in Tables 3 and 4.

### AIModules handling and limitations

Our tool can help to find conserved motifs in nucleic acids such as TFBSs using a computer with decent hardware. First the user should perform a basic motif tool to identify common TFBSs. With those well-chosen matrices a module search can be conducted to find conserved modules.

The resulting picture (see Fig. 1 or Supplementary Tutorial file) shows that binding site matches frequently overlap. These matches are filtered beforehand by the back-end via the parameters La and Ld, and therefore strong bindings and high score matches are shown. However, one must consider that these results mean that a TF would bind the binding site in vitro, which must be validated through experimentation. In addition, even if the TF binds in vitro*,* it may not necessarily play a role in gene regulation in vivo.

Another caveat is that if a TFBS is not shown in the result of an input sequence, this does not automatically mean none exist. It could mean the match was excluded by a high La or low Ld. Furthermore, the JASPAR database of matrices, while up-to-date, is not exhaustive and is enriched and optimized over time, i.e., the TF may not be available and therefore may not be included in the result. These restrictions also apply to commercial products.

Predictions should be treated as such. A match means that in vitro the corresponding TF is very likely to bind the TFBS. In vivo there are factors like interactions of the TF with chromatin (conformation) which play a crucial role. Furthermore, the quantity of available TF relative to its TFBS and the quantity of cofactors contribute greatly to TF-TFBS interactions.

## Future perspectives

First of all, AIModules can be rapidly improved by considering more motifs and protein binding sites. Important work regarding this has already been done using deep learning^26^ and by machine learning frameworks such as Tensor Flow or PyTorch, etc. In addition, hidden Markov models such as Transcription Factor Flexible Models (TFFMs), that can model positional interdependence within the TFBSs and variable length motifs^27,28^, improve the data on motifs, TFBSs, and modules and motif combinations available for AIModules. Similarly, the high-quality matrices from JASPAR can be enriched with matrices from CisBP^29^ or UniPROBE^30^ as well.

Furthermore, the filtering for modules can be improved in quality by saving conserved TFBS combinations (modules) in a fast database with their specific offsets and applying these to the found TFBSs to improve modules prediction, learning directly from the studied examples.

Since the architecture is also available as a Docker based solution (and a *docker swarm*), it should not be difficult to deploy this system onto a Kubernetes provider with high throughput. In this environment add-ons could be deployed as micro services or server-less components to increase the capacity for load balancing and failover functionality.

Another advancement would be to implement all calculation steps into the server via so called server-side rendering. This should increase the overall performance of AIModules but could also lead to delays when the server is under very high load. This is due to the first-in first-out (FIFO) principle of workloads, which is common to such web applications.

## Conclusions

AlModules is a versatile software package which allows the user to identify motifs and conserved module analysis in DNA as well as RNA. The software performs better than current alternatives and is generic, i.e. it can also be applied to look with a suitable user-specified matrix for any other motifs in DNA such as silencers or repetitive sequences or regulatory motifs in different types of RNA (catalytic RNA, piRNAs, localization motifs in mRNA). The user can thus also investigate conserved motifs of their own choice. We provide both a web-server as well as the stand-alone software for installation. We have hence a particularly flexible and easy to use solution for the interested researcher. AIModules is completely free, non-commercial and open source. We offer an easy and fast detection tool, acknowledging that after motif discovery using AIModules, a more detailed analysis should follow, including validation by experiments.

## Methods

### Architecture

**Figure 2:**
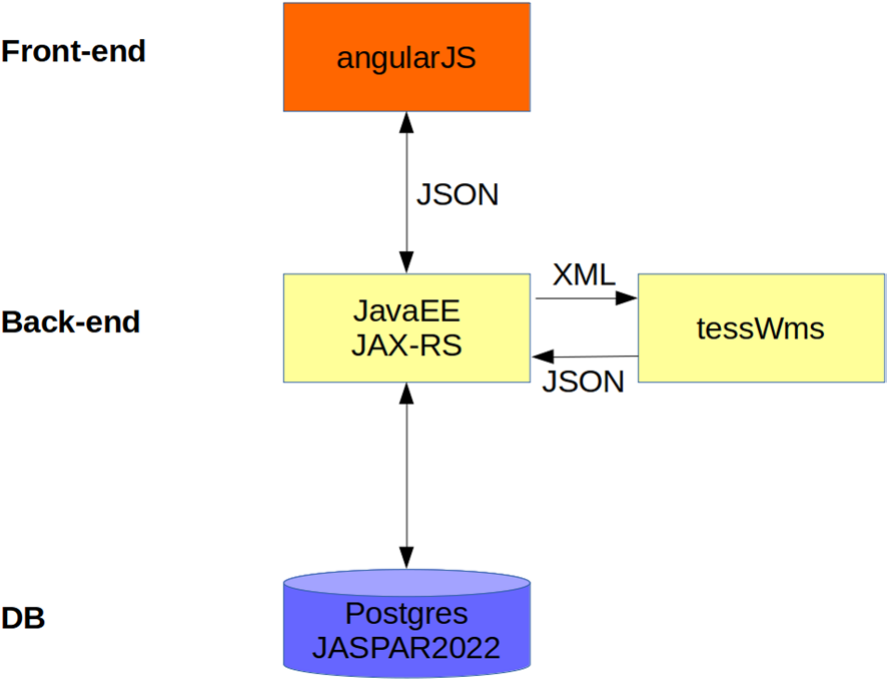
Architecture of AIModules. The three layers (front-end, back-end, DB) and the flow of information are illustrated.

We used a three-layered architecture (Fig. 2): front-end, back-end, database. The searches for TFBSs are done on the back-end, whereas the module search and result rendering are performed on the front-end. Furthermore, we prepared a Docker based solution (see Supplement Evaluation section “Build and Deploy”).

### Architecture parameters

The back-end calls tessWms to search for TFBSs with the parameters selected in the front-end. These are La and Ld. La is the log odds ratio of the match from a PWM, whereas Ld is the maximum log likelihood deficit, i.e. the difference between the maximum ratio score of a PWM (Lm), which is the consensus sequence, and the log odds ratio of the match (Ld = Lm − La)^9,10^. Each position in a binding site can contribute up to the value of two to the score. Thus, the best La corresponds to the consensus sequence (the best La is the Lm) and Ld defines how much worse the La of a TFBS is compared to Lm.

The parameters for MatInspector in the tool Genomatix on the other hand are *Optimized* and 0.75. A perfect match to the matrix means that the binding site is equal to the consensus sequence and receives a score of 1.00. *Optimized* in the context of matrix similarity within MatInspector means that the binding site is valid if the score is greater than 0.75^31^.

### Implementation

Our tool AIModules^32^ uses position frequency matrices (PFMs), introduced in 1982^33^, to predict TFBSs either from the JASPAR 2022 Database or user input PFMs. We decided against including TRANSFAC® matrices, as these are from 2005, and the 398 matrices cannot be downloaded but rather must be extracted manually from their website one by one from HTML, which is time consuming and error prone. The result of the analyses is presented to the user in graphical and file form. Apart from a conservative three-layered architecture (see Fig. 1), AIModules is also implemented and prepared as a Docker container-based solution (see Supplement Evaluation section “Build and Deploy”).

The architecture of AIModules is the product of three layers which are loosely coupled. For the view we chose the single page application framework (SPA) *angularJS* to reduce calls to the back-end. The SPA is offered on an Apache webserver. The front-end communicates with the back-end via *JSON*; the back-end itself is a JAXRS Rest service running on Apache Tomcat® 9.0.0.M1 and *JavaEE.* In this layer the executable *tessWms*^9,10,34^ is called to find TFBSs. We modified tessWms to allow for a JSON interface, hence communication from tessWms to the Java back-end is done via JSON. The user can select TFBS classes in the front-end. Those are read from the REST back-end, which communicates with the third layer—a PostgreSQL 11 DB with the JASPAR 2022 TFBSs—and presents them via specific URLs (see Supplement Evaluation section “Build and Deploy”).

Furthermore, we offer a complete docker packaged environment (see Supplement Evaluation section “Build and Deploy”).

Moreover, all mentioned databases and tools of the paper are described in Supplement Evaluation section “Development.”

## Data availability statement

All data and materials are fully available from the paper and its supplementary materials. The program sources are available via https://github.com/muharrem-aydinli/AIModules.git or https://zenodo.org/badge/latestdoi/363702392. Further project and software information: Project name: AIModules. Project home page: https://bioinfo-wuerz.de/aimodules/ or https://aimodules.heinzelab.de. Operating system(s): Platform independent, Web application. Programming language: Java, JavaScript, C, Python. Other requirements: docker, Java 1.8, Tomcat, PostgreSQL, or use our web application with a web browser. License: GNU GPL v2. Any restrictions to use by non-academics: none.

## Supplementary Information

Supplementary Information 1.

Supplementary Information 2.

Supplementary Information 3.

## Abbreviations

TF: Transcription factor
TFBS: Transcription factor binding site
