# Supplementary material for: Motif and conserved module analysis in DNA (promoters, enhancers) and RNA (lncRNA, mRNA) using AlModules
Source: Sci Rep. 2022 Oct 20;12:17588. doi: 10.1038/s41598-022-21732-0 (PMC9584888; doi:10.1038/s41598-022-21732-0)
Supplement: Supplementary file 2 — Supplementary Information 2. [file 41598_2022_21732_MOESM2_ESM.docx]

Supplementary information

**Motif and conserved module analysis in DNA (promotors, enhancers) and RNA (lncRNA, mRNA) using AlModules**

Muharrem Aydinli^1^*, Chunguang Liang^1^*, Thomas Dandekar^1,§^

^1^ Department of Bioinformatics, Biocenter, Am Hubland, D-97074 University of Würzburg

This supplement (i) evaluates and compares AIModules to alternative software and programs.

(ii) gives technical hints and details

Independent 2^nd^ file: Tutorial

Independent 3^rd^ file:

- Data-Supplements.zip (AIModules_vs_Genomatix_Crude_Results in detail are given)

Overview

[Evaluation 3](#_Toc102922382)

[Comparison to Software MatInspector from the company “Genomatix” 3](#_Toc102922383)

[Interleukin module result table 6](#_Toc102922384)

[Interleukin TFBS result table 21](#_Toc102922385)

[Poly adenylation site motif results 25](#_Toc102922386)

[Common TFBSs for AIModules vs Genomatix’s MatInspector 26](#_Toc102922387)

[Comparison of the different tools for TFBS discovery 31](#_Toc102922388)

[MotifMap (3) 31](#_Toc102922389)

[Promo (5) (6) 32](#_Toc102922390)

[ModuleMaster (7) 35](#_Toc102922391)

[Prodoric® (8) 35](#_Toc102922392)

[Softberry 36](#_Toc102922393)

[TAIR (16) 37](#_Toc102922394)

[PlantPan 3.0 (17) 38](#_Toc102922395)

[TESS - Transcription Element Search System 38](#_Toc102922396)

[Genomatix 38](#_Toc102922397)

[TRANSFAC (23), (24) 38](#_Toc102922398)

[ConTraV3 (28), (29) 39](#_Toc102922399)

[CisBP (30; 31) 39](#_Toc102922400)

[UniPROBE (32; 33) 39](#_Toc102922401)

[HOCOMOCO (34; 35) 40](#_Toc102922402)

[FlyFactorSurvey (36) 41](#_Toc102922403)

[MEME Suite (37; 38) 42](#_Toc102922404)

[YeTFaSCo (39; 40) 42](#_Toc102922405)

[Used sequences 44](#_Toc102922406)

[conTraV3 comparison 44](#_Toc102922407)

[Sequences for poly adenylation site motif analysis 44](#_Toc102922408)

[Analysis on how well AIModules recognizes motifs shown with the example of NFAT (41) 45](#_Toc102922409)

[Genomatix’s solutions vs AIModules: here IL-10 46](#_Toc102922410)

[Genomatix’s solutions vs AIModules: here cathepsins 46](#_Toc102922411)

[Homo sapiens Immunoglobulin 47](#_Toc102922412)

[Drosophila melanogaster *oskar* (osk) 47](#_Toc102922413)

[Technical Hints and Details for AIModules 48](#_Toc102922414)

[Matrix Generation 48](#_Toc102922415)

[Mathematical Consideration 50](#_Toc102922416)

[Using Docker containers 51](#_Toc102922417)

[Text field regex Warnings 51](#_Toc102922418)

[Development 53](#_Toc102922419)

[Build and Deploy 53](#_Toc102922420)

[References 55](#_Toc102922421)

# Evaluation

## Comparison to Software MatInspector from the company “Genomatix”

We compared our solution AIModules to a commercial product regarding not only the results for TFBS, but also the results from the module search.

1. First, we looked at found TFBSs
   1. For the cathepsins

Table S 1 **Parameters for TFBS search for AIModules and Genomatix’s MatInspector** (1) **for cathepsins.**

| AIModules | Genomatix (MatInspector) |
| --- | --- |
| The log likelihood ratio score (La) was set to six and the maximum log likelihood deficit to eight (Ld). These values are selected on the one hand because the README file (2) of the source code suggests this and on the other to not overwhelm the user with findings. The README file suggests the default value for La to be set to six. Furthermore, to restrict the number of findings, Ld was set to eight. These values can be used for initial calculations, but the web applications can handle higher or lower values as well. JASPAR matrices from vertebrates were selected. | The Matrix Family Library Version 11.1 was used with vertebrates matrices (0.75/Optimized) and General Core Promoter Elements (0.75/Optimized) |

Both values, the log-likelihood ratio score and maximum log likelihood deficit, were set in such a way that the results do not overwhelm the user with too much unspecific output. Regarding the log-likelihood ratio score we followed the default value that was suggested in the README file of the public source code which can be downloaded. In contrast, the “maximum log likelihood deficit” also determines the amount of output created. Its values can be modified over a wide range and the value chosen presents a conservative threshold again, so that not too much unspecific output is created but also the risk of losing a binding site is low.

One matrix can generate N Hits.

Table S 2 **TFBS search with AIModules and Genomatix’s MatInspector for cathepsins.** We analyzed three cathepsin promoters for TFBSs and AIModules generated many more TFBSs than MatInspector. Both tools share common TFBSs. (sequence names: a = Homo_sapiens_cathepsin_V_transcript_variant-1_promoter, b = Bos_taurus_cathepsin-Z_promoter, c = Mus_musculus_cathepsin-F-transcript-variant-X1_promoter_576-1076)

| Sequence name | ***a*** | ***b*** | ***c*** |
| --- | --- | --- | --- |
| AIModules TFBS count | 253 | 322 | 492 |
| Genomatix TFBS count | 155 | 224 | 166 |
| Common TFBSs or family | 15 | 21 | 24 |

AIModules found more TFBS in all cases compared to Genomatix. A small portion of the found TFBSs is shared by both systems.

- 1. for IL-10

Table S 3 **Parameters for TFBS search for AIModules and Genomatix’s MatInspector for IL-10.**

| AIModules | Genomatix (MatInspector) |
| --- | --- |
| The log likelihood ratio score was set to six and the maximum log likelihood deficit to eight. JASPAR matrices from vertebrates were selected. | The Matrix Family Library Version 11.1 was used with vertebrates matrices (0.75/Optimized) and General Core Promoter Elements (0.75/Optimized) |

One matrix can generate N Hits.

Table S 4 **TFBS search with AIModules and Genomatix’s MatInspector for IL-10.** We analyzed three IL-10 promoters. AIModules found much more TFBS in all the three cases and both applications shared common TFs. (sequence names: a = X73536.1_H.sapiens_promoter_region_of_human_IL-10_gene, b = AY486432.1_Macaca-mulatta_interleukin-10-(IL-10)_gene_promoter_region, c = AF121965.1_Mus-musculus_interleukin-10-(IL10)_gene_promoter_partial_sequence)

| Sequence name | ***a*** | ***b*** | ***c*** |
| --- | --- | --- | --- |
| AIModules TFBS count | 918 | 963 | 212 |
| Genomatix TFBS count | 289 | 350 | 93 |
| Common TFBSs or family | 36 | 49 | 16 |

AIModules found more than double the count of TFBSs than MatInspector in all the three cases and a small fraction of the TFBSs is shared between both systems.

With these settings we found for X73536.1_H.sapiens_promoter_region_of_human_IL-10_gene and AY486432.1_Macaca-mulatta_interleukin-10-(IL-10)_gene_promoter_region the TFs MA0519.1 Stat5a::Stat5b, MA0518.1 Stat4, MA0137.3 STAT1 and MA0144.2 STAT3 but not for AF121965.1_Mus-musculus_interleukin-10-(IL10)_gene_promoter_partial_sequence.

Genomatix shows the same picture with no Stat TF for AF121965.1_Mus-musculus_interleukin-10-(IL10)_gene_promoter_partial_sequence. The common TFBSs can be found in Table S 12 to Table S 17.

1. Second, we looked at the modules found
   1. For the cathepsins

Table S 5 **Parameters for module search for AIModules and Genomatix’s ModelInspector for cathepsins.**

| AIModules | Genomatix (ModelInspector) |
| --- | --- |
| The log likelihood ratio score was set to six and the maximum log likelihood deficit to eight. JASPAR matrices from vertebrates were selected. The module search checkbox was activated and the threshold for TFs to be valid for module filtering was set to 3 (the amount of user input sequences). | The Vertebrate_Modules Version 6.3 and the Matrix Family Library Version 11.1 were used. Threshold for number of elements: 100.0%  Maximum number of matches: 1000 |

All counts include modules on the *forward* and *reverse* strand.

Table S 6 **Module search with AIModules and Genomatix’s ModelInspector for cathepsins.** We analyzed three cathepsin promoters for modules in both, AIModules and ModelInspector. AIModules found many more Modules in all three cases compared to ModelInspector. There were no common modules. (sequence names: a = Homo_sapiens_cathepsin_V_transcript_variant-1_promoter, b = Bos_taurus_cathepsin-Z_promoter, c = Mus_musculus_cathepsin-F-transcript-variant-X1_promoter_576-1076)

| Sequence name | ***a*** | ***b*** | ***c*** |
| --- | --- | --- | --- |
| AIModules modules count | 486 | 1,497 | 640 |
| AIModules TFBSs count | 47 | 79 | 60 |
| Genomatix modules count | 15 | 29 | 9 |
| Common modules | 0 | 0 | 0 |

AIModules found more modules than Genomatix in all three cases – at least by a factor of 10. Common modules were not identified by manual comparison.

- 1. for IL-10

Table S 7 **Parameters for modules search for AIModules and Genomatix’s ModelInspector for IL-10.**

| AIModules | Genomatix (ModelInspector) |
| --- | --- |
| The log likelihood ratio score was increased to seven, as the calculation of the modules took a long time, and the maximum log likelihood deficit was set to eight. JASPAR matrices from vertebrates were selected. The module search checkbox was activated and the threshold for TFs to be valid for module filtering was set to 3 (the amount of user input sequences). | The Vertebrate_Modules Version 6.3 and the Matrix Family Library Version 11.1 were used. Threshold for number of elements: 100.0%  Maximum number of matches: 1000 |

All counts include modules on the *forward* and *reverse* strand.

Table S 8 **Module search with AIModules and Genomatix’s ModelInspector for IL-10.** We analyzed interleukin genes for modules in AIModules and ModelInspector. AIModules found many more modules than ModelInspector. There were no common modules. (sequence names: a = X73536.1_H.sapiens_promoter_region_of_human_IL-10_gene, b = AY486432.1_Macaca-mulatta_interleukin-10-(IL-10)_gene_promoter_region, c = AF121965.1_Mus-musculus_interleukin-10-(IL10)_gene_promoter_partial_sequence)

| Sequence name | ***a*** | ***b*** | ***c*** |
| --- | --- | --- | --- |
| AIModules modules count | 941 | 1,131 | 502 |
| AIModules TFBSs count | 62 | 80 | 52 |
| Genomatix modules count | 13 | 15 | 3 |
| Common modules | 0 | 0 | 0 |

AIModules found more modules than ModelInspector in all the three cases – at least by a factor of 10. Common modules were not identified by manual comparison. The found TFBSs and modules are documented in the zip file “Data-Supplements”. These differences in the results can easily be the effect from comparing just the freely available version of ModelInspector with AIModules. In general, the results heavily depend of course also on the database of TFBS used. We consider this result hence not an indication that our software is in any way superior or even comes close here to the services offered by the company with the name Genomatix or, in fact, any other more comprehensive analysis of promotor modules. Instead this confirms only that the tool AIModules is a useful stand-alone tool for a first, direct analysis of conserved modules in conserved promotor regions and identifies these rapid and efficiently. Results depend of course on the database and matrices used as well as the promotor regions compared and examined.

Since there are no common modules, we will not elaborate the found modules further.

## Interleukin module result table

Below is the list of the modules found for the interleukin promoters.

Table S 9 **Interleukin module result table.**

| **Sequence Name** | **Module** | **Module Start** | **Module End** | **Sense** |
| --- | --- | --- | --- | --- |
| **NC_000067.6:131019345-131019844 Mus musculus strain C57BL/6J chromosome 1, GRCm38.p6 C57BL/6J, Mus musculus interleukin 10 (Il10) promoter** | MA0868.2 SOX8::MA0152.1 NFATC2 | 98 | 148 | N |
|  | MA0868.2 SOX8::MA1525.1 NFATC4 | 98 | 183 | N |
|  | MA0152.1 NFATC2::MA1621.1 Rbpjl | 142 | 219 | N |
|  | MA0152.1 NFATC2::MA1620.1 Ptf1a(var.3) | 142 | 218 | N |
|  | MA1621.1 Rbpjl::MA1620.1 Ptf1a(var.3) | 206 | 218 | N |
|  | MA0868.2 SOX8::MA1100.2 ASCL1 | 98 | 217 | N |
|  | MA0152.1 NFATC2::MA1100.2 ASCL1 | 142 | 217 | N |
|  | MA1525.1 NFATC4::MA1100.2 ASCL1 | 174 | 217 | N |
|  | MA1621.1 Rbpjl::MA1100.2 ASCL1 | 206 | 217 | N |
|  | MA1620.1 Ptf1a(var.3)::MA1100.2 ASCL1 | 207 | 217 | N |
|  | MA0152.1 NFATC2::MA0868.2 SOX8 | 142 | 347 | N |
|  | MA1525.1 NFATC4::MA0868.2 SOX8 | 174 | 347 | N |
|  | MA1621.1 Rbpjl::MA0868.2 SOX8 | 206 | 347 | N |
|  | MA1620.1 Ptf1a(var.3)::MA0868.2 SOX8 | 207 | 347 | N |
|  | MA1100.2 ASCL1::MA0868.2 SOX8 | 208 | 347 | N |
|  | MA1621.1 Rbpjl::MA1525.1 NFATC4 | 206 | 368 | N |
|  | MA1620.1 Ptf1a(var.3)::MA1525.1 NFATC4 | 207 | 368 | N |
|  | MA1100.2 ASCL1::MA1525.1 NFATC4 | 208 | 368 | N |
|  | MA0868.2 SOX8::MA1525.1 NFATC4 | 338 | 368 | N |
|  | MA0152.1 NFATC2::MA0528.2 ZNF263 | 142 | 421 | N |
|  | MA1621.1 Rbpjl::MA0528.2 ZNF263 | 206 | 421 | N |
|  | MA1620.1 Ptf1a(var.3)::MA0528.2 ZNF263 | 207 | 421 | N |
|  | MA1100.2 ASCL1::MA0528.2 ZNF263 | 208 | 421 | N |
|  | MA0152.1 NFATC2::MA0528.2 ZNF263 | 142 | 424 | N |
|  | MA1621.1 Rbpjl::MA0528.2 ZNF263 | 206 | 424 | N |
|  | MA1620.1 Ptf1a(var.3)::MA0528.2 ZNF263 | 207 | 424 | N |
|  | MA1100.2 ASCL1::MA0528.2 ZNF263 | 208 | 424 | N |
|  | MA0528.2 ZNF263::MA0528.2 ZNF263 | 410 | 424 | N |
|  | MA1525.1 NFATC4::MA0868.2 SOX8 | 174 | 438 | N |
|  | MA1621.1 Rbpjl::MA0868.2 SOX8 | 206 | 438 | N |
|  | MA1620.1 Ptf1a(var.3)::MA0868.2 SOX8 | 207 | 438 | N |
|  | MA1100.2 ASCL1::MA0868.2 SOX8 | 208 | 438 | N |
|  | MA1525.1 NFATC4::MA0868.2 SOX8 | 359 | 438 | N |
|  | MA0528.2 ZNF263::MA0868.2 SOX8 | 410 | 438 | N |
|  | MA0528.2 ZNF263::MA0868.2 SOX8 | 413 | 438 | N |
|  | MA1525.1 NFATC4::MA0867.2 SOX4 | 174 | 439 | N |
|  | MA1621.1 Rbpjl::MA0867.2 SOX4 | 206 | 439 | N |
|  | MA1620.1 Ptf1a(var.3)::MA0867.2 SOX4 | 207 | 439 | N |
|  | MA1100.2 ASCL1::MA0867.2 SOX4 | 208 | 439 | N |
|  | MA0868.2 SOX8::MA0867.2 SOX4 | 338 | 439 | N |
|  | MA1525.1 NFATC4::MA0867.2 SOX4 | 359 | 439 | N |
|  | MA0528.2 ZNF263::MA0867.2 SOX4 | 410 | 439 | N |
|  | MA0528.2 ZNF263::MA0867.2 SOX4 | 413 | 439 | N |
|  | MA0868.2 SOX8::MA0867.2 SOX4 | 429 | 439 | N |
|  | MA0152.1 NFATC2::MA0597.1 THAP1 | 142 | 450 | N |
|  | MA1621.1 Rbpjl::MA0597.1 THAP1 | 206 | 450 | N |
|  | MA1620.1 Ptf1a(var.3)::MA0597.1 THAP1 | 207 | 450 | N |
|  | MA1100.2 ASCL1::MA0597.1 THAP1 | 208 | 450 | N |
|  | MA0528.2 ZNF263::MA0597.1 THAP1 | 410 | 450 | N |
|  | MA0528.2 ZNF263::MA0597.1 THAP1 | 413 | 450 | N |
|  | MA0868.2 SOX8::MA0816.1 Ascl2 | 98 | 217 | N |
|  | MA0152.1 NFATC2::MA0816.1 Ascl2 | 142 | 217 | N |
|  | MA1525.1 NFATC4::MA0816.1 Ascl2 | 174 | 217 | N |
|  | MA1621.1 Rbpjl::MA0816.1 Ascl2 | 206 | 217 | N |
|  | MA1620.1 Ptf1a(var.3)::MA0816.1 Ascl2 | 207 | 217 | N |
|  | MA0816.1 Ascl2::MA0868.2 SOX8 | 208 | 347 | N |
|  | MA0816.1 Ascl2::MA1525.1 NFATC4 | 208 | 368 | N |
|  | MA0816.1 Ascl2::MA0868.2 SOX8 | 208 | 438 | N |
|  | MA0816.1 Ascl2::MA0867.2 SOX4 | 208 | 439 | N |
|  | MA0868.2 SOX8::MA1472.1 BHLHA15(var.2) | 98 | 217 | N |
|  | MA0152.1 NFATC2::MA1472.1 BHLHA15(var.2) | 142 | 217 | N |
|  | MA1525.1 NFATC4::MA1472.1 BHLHA15(var.2) | 174 | 217 | N |
|  | MA0152.1 NFATC2::MA0624.1 NFATC1 | 129 | 183 | R |
|  | MA0152.1 NFATC2::MA0521.1 Tcf12 | 129 | 217 | R |
|  | MA0624.1 NFATC1::MA0521.1 Tcf12 | 174 | 217 | R |
|  | MA0152.1 NFATC2::MA0624.1 NFATC1 | 129 | 368 | R |
|  | MA0624.1 NFATC1::MA0624.1 NFATC1 | 174 | 368 | R |
|  | MA0521.1 Tcf12::MA0624.1 NFATC1 | 207 | 368 | R |
|  | MA0624.1 NFATC1::MA0152.1 NFATC2 | 174 | 367 | R |
|  | MA0521.1 Tcf12::MA0152.1 NFATC2 | 207 | 367 | R |
|  | MA0624.1 NFATC1::MA0152.1 NFATC2 | 359 | 367 | R |
|  | MA0152.1 NFATC2::MA0599.1 KLF5 | 129 | 421 | R |
|  | MA0624.1 NFATC1::MA0599.1 KLF5 | 174 | 421 | R |
|  | MA0521.1 Tcf12::MA0599.1 KLF5 | 207 | 421 | R |
|  | MA0624.1 NFATC1::MA0599.1 KLF5 | 359 | 421 | R |
|  | MA0152.1 NFATC2::MA0599.1 KLF5 | 361 | 421 | R |
|  | MA0152.1 NFATC2::MA0625.1 NFATC3 | 129 | 368 | R |
|  | MA0624.1 NFATC1::MA0625.1 NFATC3 | 174 | 368 | R |
|  | MA0521.1 Tcf12::MA0625.1 NFATC3 | 207 | 368 | R |
|  | MA0625.1 NFATC3::MA0152.1 NFATC2 | 359 | 367 | R |
|  | MA0625.1 NFATC3::MA0599.1 KLF5 | 359 | 421 | R |
| **NC_000074.6:c82403252-82402576 Mus musculus strain C57BL/6J chromosome 8, GRCm38.p6 C57BL/6J, Mus musculus interleukin 15 (Il15), transcript variant 2 promoter** | MA0152.1 NFATC2::MA0528.2 ZNF263 | 12 | 208 | N |
|  | MA0152.1 NFATC2::MA0597.1 THAP1 | 12 | 234 | N |
|  | MA0528.2 ZNF263::MA0597.1 THAP1 | 197 | 234 | N |
|  | MA0152.1 NFATC2::MA1621.1 Rbpjl | 12 | 288 | N |
|  | MA0528.2 ZNF263::MA1621.1 Rbpjl | 197 | 288 | N |
|  | MA0152.1 NFATC2::MA1620.1 Ptf1a(var.3) | 12 | 287 | N |
|  | MA0528.2 ZNF263::MA1620.1 Ptf1a(var.3) | 197 | 287 | N |
|  | MA1621.1 Rbpjl::MA1620.1 Ptf1a(var.3) | 275 | 287 | N |
|  | MA0152.1 NFATC2::MA1100.2 ASCL1 | 12 | 286 | N |
|  | MA0528.2 ZNF263::MA1100.2 ASCL1 | 197 | 286 | N |
|  | MA1621.1 Rbpjl::MA1100.2 ASCL1 | 275 | 286 | N |
|  | MA1620.1 Ptf1a(var.3)::MA1100.2 ASCL1 | 276 | 286 | N |
|  | MA0152.1 NFATC2::MA0597.1 THAP1 | 12 | 317 | N |
|  | MA0528.2 ZNF263::MA0597.1 THAP1 | 197 | 317 | N |
|  | MA1621.1 Rbpjl::MA0597.1 THAP1 | 275 | 317 | N |
|  | MA1620.1 Ptf1a(var.3)::MA0597.1 THAP1 | 276 | 317 | N |
|  | MA1100.2 ASCL1::MA0597.1 THAP1 | 277 | 317 | N |
|  | MA1621.1 Rbpjl::MA1100.2 ASCL1 | 275 | 478 | N |
|  | MA1620.1 Ptf1a(var.3)::MA1100.2 ASCL1 | 276 | 478 | N |
|  | MA0152.1 NFATC2::MA0597.1 THAP1 | 12 | 482 | N |
|  | MA1621.1 Rbpjl::MA0597.1 THAP1 | 275 | 482 | N |
|  | MA1620.1 Ptf1a(var.3)::MA0597.1 THAP1 | 276 | 482 | N |
|  | MA1100.2 ASCL1::MA0597.1 THAP1 | 277 | 482 | N |
|  | MA1100.2 ASCL1::MA0597.1 THAP1 | 469 | 482 | N |
|  | MA0528.2 ZNF263::MA0868.2 SOX8 | 197 | 561 | N |
|  | MA0597.1 THAP1::MA0868.2 SOX8 | 226 | 561 | N |
|  | MA1621.1 Rbpjl::MA0868.2 SOX8 | 275 | 561 | N |
|  | MA1620.1 Ptf1a(var.3)::MA0868.2 SOX8 | 276 | 561 | N |
|  | MA1100.2 ASCL1::MA0868.2 SOX8 | 277 | 561 | N |
|  | MA0597.1 THAP1::MA0868.2 SOX8 | 309 | 561 | N |
|  | MA1100.2 ASCL1::MA0868.2 SOX8 | 469 | 561 | N |
|  | MA0597.1 THAP1::MA0868.2 SOX8 | 474 | 561 | N |
|  | MA0528.2 ZNF263::MA0867.2 SOX4 | 197 | 562 | N |
|  | MA0597.1 THAP1::MA0867.2 SOX4 | 226 | 562 | N |
|  | MA1621.1 Rbpjl::MA0867.2 SOX4 | 275 | 562 | N |
|  | MA1620.1 Ptf1a(var.3)::MA0867.2 SOX4 | 276 | 562 | N |
|  | MA1100.2 ASCL1::MA0867.2 SOX4 | 277 | 562 | N |
|  | MA0597.1 THAP1::MA0867.2 SOX4 | 309 | 562 | N |
|  | MA1100.2 ASCL1::MA0867.2 SOX4 | 469 | 562 | N |
|  | MA0597.1 THAP1::MA0867.2 SOX4 | 474 | 562 | N |
|  | MA0868.2 SOX8::MA0867.2 SOX4 | 552 | 562 | N |
|  | MA0528.2 ZNF263::MA1525.1 NFATC4 | 197 | 566 | N |
|  | MA1621.1 Rbpjl::MA1525.1 NFATC4 | 275 | 566 | N |
|  | MA1620.1 Ptf1a(var.3)::MA1525.1 NFATC4 | 276 | 566 | N |
|  | MA1100.2 ASCL1::MA1525.1 NFATC4 | 277 | 566 | N |
|  | MA0597.1 THAP1::MA1525.1 NFATC4 | 309 | 566 | N |
|  | MA1100.2 ASCL1::MA1525.1 NFATC4 | 469 | 566 | N |
|  | MA0597.1 THAP1::MA1525.1 NFATC4 | 474 | 566 | N |
|  | MA0868.2 SOX8::MA1525.1 NFATC4 | 552 | 566 | N |
|  | MA0528.2 ZNF263::MA0152.1 NFATC2 | 197 | 578 | N |
|  | MA0597.1 THAP1::MA0152.1 NFATC2 | 226 | 578 | N |
|  | MA1621.1 Rbpjl::MA0152.1 NFATC2 | 275 | 578 | N |
|  | MA1620.1 Ptf1a(var.3)::MA0152.1 NFATC2 | 276 | 578 | N |
|  | MA1100.2 ASCL1::MA0152.1 NFATC2 | 277 | 578 | N |
|  | MA0597.1 THAP1::MA0152.1 NFATC2 | 309 | 578 | N |
|  | MA1100.2 ASCL1::MA0152.1 NFATC2 | 469 | 578 | N |
|  | MA0597.1 THAP1::MA0152.1 NFATC2 | 474 | 578 | N |
|  | MA0868.2 SOX8::MA0152.1 NFATC2 | 552 | 578 | N |
|  | MA1525.1 NFATC4::MA0152.1 NFATC2 | 557 | 578 | N |
|  | MA0528.2 ZNF263::MA0152.1 NFATC2 | 197 | 634 | N |
|  | MA0597.1 THAP1::MA0152.1 NFATC2 | 226 | 634 | N |
|  | MA1621.1 Rbpjl::MA0152.1 NFATC2 | 275 | 634 | N |
|  | MA1620.1 Ptf1a(var.3)::MA0152.1 NFATC2 | 276 | 634 | N |
|  | MA1100.2 ASCL1::MA0152.1 NFATC2 | 277 | 634 | N |
|  | MA0597.1 THAP1::MA0152.1 NFATC2 | 309 | 634 | N |
|  | MA1100.2 ASCL1::MA0152.1 NFATC2 | 469 | 634 | N |
|  | MA0597.1 THAP1::MA0152.1 NFATC2 | 474 | 634 | N |
|  | MA0868.2 SOX8::MA0152.1 NFATC2 | 552 | 634 | N |
|  | MA1525.1 NFATC4::MA0152.1 NFATC2 | 557 | 634 | N |
|  | MA0868.2 SOX8::MA1100.2 ASCL1 | 552 | 645 | N |
|  | MA1525.1 NFATC4::MA1100.2 ASCL1 | 557 | 645 | N |
|  | MA0152.1 NFATC2::MA1100.2 ASCL1 | 572 | 645 | N |
|  | MA0152.1 NFATC2::MA1100.2 ASCL1 | 628 | 645 | N |
|  | MA0528.2 ZNF263::MA0816.1 Ascl2 | 197 | 645 | N |
|  | MA0597.1 THAP1::MA0816.1 Ascl2 | 226 | 645 | N |
|  | MA1621.1 Rbpjl::MA0816.1 Ascl2 | 275 | 645 | N |
|  | MA1620.1 Ptf1a(var.3)::MA0816.1 Ascl2 | 276 | 645 | N |
|  | MA1100.2 ASCL1::MA0816.1 Ascl2 | 277 | 645 | N |
|  | MA0597.1 THAP1::MA0816.1 Ascl2 | 309 | 645 | N |
|  | MA1100.2 ASCL1::MA0816.1 Ascl2 | 469 | 645 | N |
|  | MA0597.1 THAP1::MA0816.1 Ascl2 | 474 | 645 | N |
|  | MA0868.2 SOX8::MA0816.1 Ascl2 | 552 | 645 | N |
|  | MA0867.2 SOX4::MA0816.1 Ascl2 | 553 | 645 | N |
|  | MA1525.1 NFATC4::MA0816.1 Ascl2 | 557 | 645 | N |
|  | MA0152.1 NFATC2::MA0816.1 Ascl2 | 572 | 645 | N |
|  | MA0152.1 NFATC2::MA0816.1 Ascl2 | 628 | 645 | N |
|  | MA0528.2 ZNF263::MA1472.1 BHLHA15(var.2) | 197 | 645 | N |
|  | MA0597.1 THAP1::MA1472.1 BHLHA15(var.2) | 226 | 645 | N |
|  | MA1621.1 Rbpjl::MA1472.1 BHLHA15(var.2) | 275 | 645 | N |
|  | MA1620.1 Ptf1a(var.3)::MA1472.1 BHLHA15(var.2) | 276 | 645 | N |
|  | MA1100.2 ASCL1::MA1472.1 BHLHA15(var.2) | 277 | 645 | N |
|  | MA0597.1 THAP1::MA1472.1 BHLHA15(var.2) | 309 | 645 | N |
|  | MA1100.2 ASCL1::MA1472.1 BHLHA15(var.2) | 469 | 645 | N |
|  | MA0597.1 THAP1::MA1472.1 BHLHA15(var.2) | 474 | 645 | N |
|  | MA0868.2 SOX8::MA1472.1 BHLHA15(var.2) | 552 | 645 | N |
|  | MA0867.2 SOX4::MA1472.1 BHLHA15(var.2) | 553 | 645 | N |
|  | MA1525.1 NFATC4::MA1472.1 BHLHA15(var.2) | 557 | 645 | N |
|  | MA0152.1 NFATC2::MA1472.1 BHLHA15(var.2) | 572 | 645 | N |
|  | MA0152.1 NFATC2::MA1472.1 BHLHA15(var.2) | 628 | 645 | N |
|  | MA0528.2 ZNF263::MA0816.1 Ascl2 | 197 | 478 | N |
|  | MA0597.1 THAP1::MA0816.1 Ascl2 | 226 | 478 | N |
|  | MA1621.1 Rbpjl::MA0816.1 Ascl2 | 275 | 478 | N |
|  | MA1620.1 Ptf1a(var.3)::MA0816.1 Ascl2 | 276 | 478 | N |
|  | MA1100.2 ASCL1::MA0816.1 Ascl2 | 277 | 478 | N |
|  | MA0597.1 THAP1::MA0816.1 Ascl2 | 309 | 478 | N |
|  | MA0816.1 Ascl2::MA0868.2 SOX8 | 469 | 561 | N |
|  | MA0816.1 Ascl2::MA0867.2 SOX4 | 469 | 562 | N |
|  | MA0816.1 Ascl2::MA1525.1 NFATC4 | 469 | 566 | N |
|  | MA0624.1 NFATC1::MA0152.1 NFATC2 | 399 | 407 | R |
|  | MA0624.1 NFATC1::MA0624.1 NFATC1 | 399 | 566 | R |
|  | MA0152.1 NFATC2::MA0624.1 NFATC1 | 401 | 566 | R |
|  | MA0152.1 NFATC2::MA0521.1 Tcf12 | 401 | 645 | R |
|  | MA0624.1 NFATC1::MA0521.1 Tcf12 | 557 | 645 | R |
|  | MA0624.1 NFATC1::MA0625.1 NFATC3 | 399 | 566 | R |
|  | MA0152.1 NFATC2::MA0625.1 NFATC3 | 401 | 566 | R |
|  | MA0625.1 NFATC3::MA0152.1 NFATC2 | 399 | 407 | R |
| **NC_000077.6:c53635202-53634702 Mus musculus strain C57BL/6J chromosome 11, GRCm38.p6 C57BL/6J, Mus musculus interleukin 13 (Il13) promoter** | MA0528.2 ZNF263::MA1621.1 Rbpjl | 82 | 160 | N |
|  | MA0528.2 ZNF263::MA1620.1 Ptf1a(var.3) | 82 | 159 | N |
|  | MA1621.1 Rbpjl::MA1620.1 Ptf1a(var.3) | 147 | 159 | N |
|  | MA0528.2 ZNF263::MA1100.2 ASCL1 | 82 | 158 | N |
|  | MA1621.1 Rbpjl::MA1100.2 ASCL1 | 147 | 158 | N |
|  | MA1620.1 Ptf1a(var.3)::MA1100.2 ASCL1 | 148 | 158 | N |
|  | MA0528.2 ZNF263::MA0597.1 THAP1 | 82 | 162 | N |
|  | MA1621.1 Rbpjl::MA0597.1 THAP1 | 147 | 162 | N |
|  | MA1620.1 Ptf1a(var.3)::MA0597.1 THAP1 | 148 | 162 | N |
|  | MA1100.2 ASCL1::MA0597.1 THAP1 | 149 | 162 | N |
|  | MA0528.2 ZNF263::MA0528.2 ZNF263 | 82 | 254 | N |
|  | MA1621.1 Rbpjl::MA0528.2 ZNF263 | 147 | 254 | N |
|  | MA1620.1 Ptf1a(var.3)::MA0528.2 ZNF263 | 148 | 254 | N |
|  | MA1100.2 ASCL1::MA0528.2 ZNF263 | 149 | 254 | N |
|  | MA0528.2 ZNF263::MA1525.1 NFATC4 | 82 | 284 | N |
|  | MA1621.1 Rbpjl::MA1525.1 NFATC4 | 147 | 284 | N |
|  | MA1620.1 Ptf1a(var.3)::MA1525.1 NFATC4 | 148 | 284 | N |
|  | MA1100.2 ASCL1::MA1525.1 NFATC4 | 149 | 284 | N |
|  | MA0597.1 THAP1::MA1525.1 NFATC4 | 154 | 284 | N |
|  | MA0528.2 ZNF263::MA0152.1 NFATC2 | 82 | 375 | N |
|  | MA1621.1 Rbpjl::MA0152.1 NFATC2 | 147 | 375 | N |
|  | MA1620.1 Ptf1a(var.3)::MA0152.1 NFATC2 | 148 | 375 | N |
|  | MA1100.2 ASCL1::MA0152.1 NFATC2 | 149 | 375 | N |
|  | MA0597.1 THAP1::MA0152.1 NFATC2 | 154 | 375 | N |
|  | MA1525.1 NFATC4::MA0152.1 NFATC2 | 275 | 375 | N |
|  | MA0528.2 ZNF263::MA0868.2 SOX8 | 82 | 438 | N |
|  | MA1621.1 Rbpjl::MA0868.2 SOX8 | 147 | 438 | N |
|  | MA1620.1 Ptf1a(var.3)::MA0868.2 SOX8 | 148 | 438 | N |

## Interleukin TFBS result table

Below is the complete table of the TFs that are included in the modules. The modules are also comprised of nuclear factor of activated T cells (NFAT) TFBSs, which was shown to be implicated in the expression of the cytokine interleukin-2. Our analysis shows that NFAT transcription factors are also part of the promoters of other interleukins (Mus musculus interleukin 10, Mus musculus interleukin 13, Mus musculus interleukin 15).

Table S 10 **Interleukin TFBS result table.**

| **Sequence Name** | **Sequence Length** | **Matrix Title** | **Matrix Length** | **Hit No.** | **Hit Sense** | **Hit Start** | **Hit Stop** | **Hit Score (La)** | **Hit Max log likelihood ratio score (Lm)** | **Difference (Ld) (maxscore(Lm) - score(La))** | **Hit Oligo** |
| --- | --- | --- | --- | --- | --- | --- | --- | --- | --- | --- | --- |
| **NC_000067.6:131019345-131019844 Mus musculus strain C57BL/6J chromosome 1, GRCm38.p6 C57BL/6J, Mus musculus interleukin 10 (Il10) promoter** | 500 | MA1100.2 ASCL1 | 10 | 1 | N | 208 | 217 | 11.290903 | 13.919985 | 2.629083 | AACAGCTGTC |
|  |  | MA0816.1 Ascl2 | 10 | 1 | N | 208 | 217 | 14.006852 | 16.538884 | 2.532033 | AACAGCTGTC |
|  |  | MA1472.1 BHLHA15(var.2) | 10 | 1 | N | 208 | 217 | 13.463911 | 13.956604 | 0.492693 | AACAGCTGTC |
|  |  | MA0599.1 KLF5 | 10 | 1 | R | 412 | 421 | 9.668823 | 15.543266 | 5.874443 | AGGGAGGAGG |
|  |  | MA0624.1 NFATC1 | 10 | 1 | R | 174 | 183 | 8.210574 | 12.519140 | 4.308567 | AATGGAATCC |
|  |  |  |  | 2 | R | 359 | 368 | 9.609372 | 12.519140 | 2.909768 | AAAGGAAAAA |
|  |  | MA0152.1 NFATC2 | 7 | 1 | N | 142 | 148 | 8.063484 | 12.341468 | 4.277985 | TTATCCA |
|  |  |  |  | 2 | R | 129 | 135 | 8.254006 | 12.341468 | 4.087463 | TGGAATA |
|  |  |  |  | 3 | R | 361 | 367 | 10.239107 | 12.341468 | 2.102362 | AGGAAAA |
|  |  | MA0625.1 NFATC3 | 10 | 1 | R | 359 | 368 | 10.362899 | 13.675168 | 3.312269 | AAAGGAAAAA |
|  |  | MA1525.1 NFATC4 | 10 | 1 | N | 174 | 183 | 8.090887 | 14.392408 | 6.301521 | AATGGAATCC |
|  |  |  |  | 2 | N | 359 | 368 | 8.522672 | 14.392408 | 5.869736 | AAAGGAAAAA |
|  |  | MA1620.1 Ptf1a(var.3) | 12 | 1 | N | 207 | 218 | 11.556983 | 15.503908 | 3.946925 | GAACAGCTGTCT |
|  |  | MA1621.1 Rbpjl | 14 | 1 | N | 206 | 219 | 11.141220 | 15.911069 | 4.769850 | AGAACAGCTGTCTG |
|  |  | MA0867.2 SOX4 | 10 | 1 | N | 430 | 439 | 9.728276 | 14.601566 | 4.873290 | TAACAAAAAC |
|  |  | MA0868.2 SOX8 | 10 | 1 | N | 98 | 107 | 8.181533 | 14.854627 | 6.673094 | GAAACAATTA |
|  |  |  |  | 2 | N | 338 | 347 | 8.322264 | 14.854627 | 6.532362 | CTTACAATGC |
|  |  |  |  | 3 | N | 429 | 438 | 8.402450 | 14.854627 | 6.452176 | ATAACAAAAA |
|  |  | MA0521.1 Tcf12 | 11 | 1 | R | 207 | 217 | 13.002089 | 15.776101 | 2.774012 | GAACAGCTGTC |
|  |  | MA0597.1 THAP1 | 9 | 1 | N | 442 | 450 | 8.693273 | 11.314089 | 2.620816 | TTGCCAGGA |
|  |  | MA0528.2 ZNF263 | 12 | 1 | N | 410 | 421 | 15.152542 | 16.915819 | 1.763277 | AGAGGGAGGAGG |
|  |  |  |  | 2 | N | 413 | 424 | 10.804723 | 16.915819 | 6.111095 | GGGAGGAGGAGC |
| **NC_000074.6:c82403252-82402576 Mus musculus strain C57BL/6J chromosome 8, GRCm38.p6 C57BL/6J, Mus musculus interleukin 15 (Il15), transcript variant 2 promoter** | 666 | MA1100.2 ASCL1 | 10 | 1 | N | 277 | 286 | 9.914553 | 13.919985 | 4.005432 | GGCACCTGGT |
|  |  |  |  | 2 | N | 469 | 478 | 9.582197 | 13.919985 | 4.337788 | GGCTGCTGCC |
|  |  |  |  | 3 | N | 636 | 645 | 10.315605 | 13.919985 | 3.604380 | TCCAGCTGCC |
|  |  | MA0816.1 Ascl2 | 10 | 1 | N | 469 | 478 | 9.355673 | 16.538884 | 7.183212 | GGCTGCTGCC |
|  |  |  |  | 2 | N | 636 | 645 | 9.632292 | 16.538884 | 6.906592 | TCCAGCTGCC |
|  |  | MA1472.1 BHLHA15(var.2) | 10 | 1 | N | 636 | 645 | 10.132307 | 13.956604 | 3.824296 | TCCAGCTGCC |
|  |  | MA0624.1 NFATC1 | 10 | 1 | R | 399 | 408 | 8.079574 | 12.519140 | 4.439567 | GCTGGAAAGC |
|  |  |  |  | 2 | R | 557 | 566 | 9.664478 | 12.519140 | 2.854663 | AATGGAATTT |
|  |  | MA0152.1 NFATC2 | 7 | 1 | N | 12 | 18 | 8.473572 | 12.341468 | 3.867896 | TTTTCCG |
|  |  |  |  | 2 | N | 572 | 578 | 8.473572 | 12.341468 | 3.867896 | TTTTCCG |
|  |  |  |  | 3 | N | 628 | 634 | 9.852084 | 12.341468 | 2.489385 | TTTTCCC |
|  |  |  |  | 4 | R | 401 | 407 | 8.323546 | 12.341468 | 4.017922 | TGGAAAG |
|  |  | MA0625.1 NFATC3 | 10 | 1 | R | 399 | 408 | 8.040663 | 13.675168 | 5.634504 | GCTGGAAAGC |
|  |  |  |  | 2 | R | 557 | 566 | 9.561851 | 13.675168 | 4.113317 | AATGGAATTT |
|  |  | MA1525.1 NFATC4 | 10 | 1 | N | 557 | 566 | 8.573176 | 14.392408 | 5.819232 | AATGGAATTT |
|  |  | MA1620.1 Ptf1a(var.3) | 12 | 1 | N | 276 | 287 | 9.691503 | 15.503908 | 5.812405 | GGGCACCTGGTA |
|  |  | MA1621.1 Rbpjl | 14 | 1 | N | 275 | 288 | 9.349614 | 15.911069 | 6.561456 | TGGGCACCTGGTAA |
|  |  | MA0867.2 SOX4 | 10 | 1 | N | 553 | 562 | 11.818057 | 14.601566 | 2.783508 | CAACAATGGA |
|  |  | MA0868.2 SOX8 | 10 | 1 | N | 552 | 561 | 11.378412 | 14.854627 | 3.476215 | TCAACAATGG |
|  |  | MA0521.1 Tcf12 | 11 | 1 | R | 635 | 645 | 13.813023 | 15.776101 | 1.963078 | CTCCAGCTGCC |
|  |  | MA0597.1 THAP1 | 9 | 1 | N | 226 | 234 | 8.221750 | 11.314089 | 3.092340 | TTGCCCTTC |
|  |  |  |  | 2 | N | 309 | 317 | 8.207882 | 11.314089 | 3.106207 | CCTCCCGCA |
|  |  |  |  | 3 | N | 474 | 482 | 8.355262 | 11.314089 | 2.958828 | CTGCCCTGC |
|  |  | MA0528.2 ZNF263 | 12 | 1 | N | 197 | 208 | 14.731682 | 16.915819 | 2.184136 | AGGGGGAGGAGA |
| **NC_000077.6:c53635202-53634702 Mus musculus strain C57BL/6J chromosome 11, GRCm38.p6 C57BL/6J, Mus musculus interleukin 13 (Il13) promoter** | 500 | MA1100.2 ASCL1 | 10 | 1 | N | 149 | 158 | 8.332904 | 13.919985 | 5.587081 | CTCACCTGCC |
|  |  | MA0816.1 Ascl2 | 10 | 1 | N | 475 | 484 | 9.583758 | 16.538884 | 6.955126 | AAAAGCTGCT |
|  |  | MA1472.1 BHLHA15(var.2) | 10 | 1 | N | 475 | 484 | 8.229655 | 13.956604 | 5.726949 | AAAAGCTGCT |
|  |  | MA0599.1 KLF5 | 10 | 1 | R | 447 | 456 | 11.424883 | 15.543266 | 4.118383 | GGGGTGAGGC |
|  |  | MA0624.1 NFATC1 | 10 | 1 | R | 233 | 242 | 8.219935 | 12.519140 | 4.299206 | GGTGGAATTA |
|  |  |  |  | 2 | R | 275 | 284 | 10.054376 | 12.519140 | 2.464765 | CGTGGAAATA |
|  |  | MA0152.1 NFATC2 | 7 | 1 | N | 369 | 375 | 12.341468 | 12.341468 | 0.000000 | TTTTCCA |
|  |  |  |  | 2 | R | 277 | 283 | 9.702058 | 12.341468 | 2.639410 | TGGAAAT |
|  |  |  |  | 3 | R | 299 | 305 | 9.171543 | 12.341468 | 3.169925 | TGGAAAC |
|  |  | MA0625.1 NFATC3 | 10 | 1 | R | 275 | 284 | 9.975994 | 13.675168 | 3.699174 | CGTGGAAATA |
|  |  | MA1525.1 NFATC4 | 10 | 1 | N | 275 | 284 | 9.930748 | 14.392408 | 4.461660 | CGTGGAAATA |
|  |  | MA1620.1 Ptf1a(var.3) | 12 | 1 | N | 148 | 159 | 10.008048 | 15.503908 | 5.495860 | ACTCACCTGCCC |
|  |  | MA1621.1 Rbpjl | 14 | 1 | N | 147 | 160 | 10.199248 | 15.911069 | 5.711821 | TACTCACCTGCCCA |
|  |  | MA0867.2 SOX4 | 10 | 1 | N | 430 | 439 | 11.560954 | 14.601566 | 3.040612 | CAACAAAGCA |
|  |  | MA0868.2 SOX8 | 10 | 1 | N | 429 | 438 | 8.156682 | 14.854627 | 6.697945 | CCAACAAAGC |
|  |  | MA0521.1 Tcf12 | 11 | 1 | R | 148 | 158 | 8.710154 | 15.776101 | 7.065947 | ACTCACCTGCC |
|  |  | MA0597.1 THAP1 | 9 | 1 | N | 154 | 162 | 9.368914 | 11.314089 | 1.945175 | CTGCCCAAA |
|  |  | MA0528.2 ZNF263 | 12 | 1 | N | 82 | 93 | 13.683952 | 16.915819 | 3.231867 | GAAGGGAGGAAG |
|  |  |  |  | 2 | N | 243 | 254 | 9.365203 | 16.915819 | 7.550616 | CTGGGGCGGAAG |

## Poly adenylation site motif results

Here are the complete results from the polyadenylation site search.

Table S 11 **Complete result for poly adenylation site motif.** The results from the antisense strand (R) apply only to DNA.

| **Sequence Name** | **Sequence Length** | **Matrix Title** | **Matrix Length** | **Hit No.** | **Hit Sense** | **Hit Start** | **Hit Stop** | **Hit Score (La)** | **Hit Max log likelihood ratio score (Lm) or matrix possible** | **Difference (Ld) (maxscore(Lm) - score(La))** | **Hit Oligo** |
| --- | --- | --- | --- | --- | --- | --- | --- | --- | --- | --- | --- |
| **NM_000600.5 Homo sapiens interleukin 6 (IL6), transcript variant 1, mRNA** | 1127 | human-Poly-A | 6 | 1 | N | 799 | 804 | 7.002311 | 9.889837 | 2.887525 | ACTAAA |
|  |  |  |  | 2 | N | 1025 | 1030 | 9.889837 | 9.889837 | 0.000000 | AATAAA |
|  |  |  |  | 3 | N | 1054 | 1059 | 6.854213 | 9.889837 | 3.035624 | AAGAAA |
|  |  |  |  | 4 | N | 1084 | 1089 | 7.002311 | 9.889837 | 2.887525 | TATAAA |
|  |  |  |  | 5 | N | 1103 | 1108 | 9.889837 | 9.889837 | 0.000000 | AATAAA |
|  |  |  |  | 6 | N | 1118 | 1123 | 6.854213 | 9.889837 | 3.035624 | AAAAAA |
|  |  |  |  | 7 | R | 827 | 832 | 7.002311 | 9.889837 | 2.887525 | TTTAAT |
|  |  |  |  | 8 | R | 837 | 842 | 7.002311 | 9.889837 | 2.887525 | TTTAAT |
|  |  |  |  | 9 | R | 842 | 847 | 9.889837 | 9.889837 | 0.000000 | TTTATT |
|  |  |  |  | 10 | R | 876 | 881 | 7.002311 | 9.889837 | 2.887525 | TTTATG |
|  |  |  |  | 11 | R | 891 | 896 | 7.002311 | 9.889837 | 2.887525 | TTTATA |
|  |  |  |  | 12 | R | 893 | 898 | 7.002311 | 9.889837 | 2.887525 | TATATT |
|  |  |  |  | 13 | R | 923 | 928 | 7.002311 | 9.889837 | 2.887525 | TTTATG |
|  |  |  |  | 14 | R | 927 | 932 | 7.002311 | 9.889837 | 2.887525 | TGTATT |
|  |  |  |  | 15 | R | 998 | 1003 | 6.854213 | 9.889837 | 3.035624 | TTTCTT |
|  |  |  |  | 16 | R | 1062 | 1067 | 7.002311 | 9.889837 | 2.887525 | TTTATA |
|  |  |  |  | 17 | R | 1064 | 1069 | 7.002311 | 9.889837 | 2.887525 | TATATT |
|  |  |  |  | 18 | R | 1069 | 1074 | 7.002311 | 9.889837 | 2.887525 | TGTATT |
|  |  |  |  | 19 | R | 1073 | 1078 | 7.002311 | 9.889837 | 2.887525 | TTTATA |
|  |  |  |  | 20 | R | 1095 | 1100 | 7.002311 | 9.889837 | 2.887525 | TTTATA |
| **NM_000594.4 Homo sapiens tumor necrosis factor (TNF), mRNA** | 1678 | human-Poly-A | 6 | 1 | N | 1655 | 1660 | 9.889837 | 9.889837 | 0.000000 | AATAAA |
|  |  |  |  | 2 | N | 1673 | 1678 | 6.854213 | 9.889837 | 3.035624 | AAGAAA |
|  |  |  |  | 3 | R | 928 | 933 | 9.889837 | 9.889837 | 0.000000 | TTTATT |
|  |  |  |  | 4 | R | 1319 | 1324 | 7.002311 | 9.889837 | 2.887525 | TCTATT |
|  |  |  |  | 5 | R | 1323 | 1328 | 7.002311 | 9.889837 | 2.887525 | TTTATG |
|  |  |  |  | 6 | R | 1345 | 1350 | 9.889837 | 9.889837 | 0.000000 | TTTATT |
|  |  |  |  | 7 | R | 1352 | 1357 | 9.889837 | 9.889837 | 0.000000 | TTTATT |
|  |  |  |  | 8 | R | 1356 | 1361 | 9.889837 | 9.889837 | 0.000000 | TTTATT |
|  |  |  |  | 9 | R | 1363 | 1368 | 9.889837 | 9.889837 | 0.000000 | TTTATT |
|  |  |  |  | 10 | R | 1367 | 1372 | 9.889837 | 9.889837 | 0.000000 | TTTATT |
|  |  |  |  | 11 | R | 1383 | 1388 | 7.002311 | 9.889837 | 2.887525 | TGTATT |
|  |  |  |  | 12 | R | 1387 | 1392 | 9.889837 | 9.889837 | 0.000000 | TTTATT |
|  |  |  |  | 13 | R | 1526 | 1531 | 6.854213 | 9.889837 | 3.035624 | TTTTTT |
|  |  |  |  | 14 | R | 1538 | 1543 | 7.002311 | 9.889837 | 2.887525 | TTTATC |
| **NM_020525.5 Homo sapiens interleukin 22 (IL22), mRNA** | 1165 | human-Poly-A | 6 | 1 | N | 900 | 905 | 7.002311 | 9.889837 | 2.887525 | CATAAA |
|  |  |  |  | 2 | N | 903 | 908 | 6.854213 | 9.889837 | 3.035624 | AAAAAA |
|  |  |  |  | 3 | N | 933 | 938 | 6.854213 | 9.889837 | 3.035624 | AAAAAA |
|  |  |  |  | 4 | N | 981 | 986 | 7.002311 | 9.889837 | 2.887525 | TATAAA |
|  |  |  |  | 5 | N | 1144 | 1149 | 9.889837 | 9.889837 | 0.000000 | AATAAA |
|  |  |  |  | 6 | R | 579 | 584 | 7.002311 | 9.889837 | 2.887525 | TTTATG |
|  |  |  |  | 7 | R | 683 | 688 | 6.854213 | 9.889837 | 3.035624 | TTTTTT |
|  |  |  |  | 8 | R | 684 | 689 | 6.854213 | 9.889837 | 3.035624 | TTTTTT |
|  |  |  |  | 9 | R | 685 | 690 | 6.854213 | 9.889837 | 3.035624 | TTTTTT |
|  |  |  |  | 10 | R | 786 | 791 | 7.002311 | 9.889837 | 2.887525 | TTTATA |
|  |  |  |  | 11 | R | 824 | 829 | 9.889837 | 9.889837 | 0.000000 | TTTATT |
|  |  |  |  | 12 | R | 873 | 878 | 9.889837 | 9.889837 | 0.000000 | TTTATT |
|  |  |  |  | 13 | R | 877 | 882 | 6.854213 | 9.889837 | 3.035624 | TTTTTT |
|  |  |  |  | 14 | R | 973 | 978 | 7.002311 | 9.889837 | 2.887525 | TTTATA |
|  |  |  |  | 15 | R | 975 | 980 | 7.002311 | 9.889837 | 2.887525 | TATATT |
|  |  |  |  | 16 | R | 979 | 984 | 7.002311 | 9.889837 | 2.887525 | TTTATA |
|  |  |  |  | 17 | R | 987 | 992 | 7.002311 | 9.889837 | 2.887525 | TGTATT |
|  |  |  |  | 18 | R | 991 | 996 | 9.889837 | 9.889837 | 0.000000 | TTTATT |
|  |  |  |  | 19 | R | 1015 | 1020 | 9.889837 | 9.889837 | 0.000000 | TTTATT |
|  |  |  |  | 20 | R | 1019 | 1024 | 7.002311 | 9.889837 | 2.887525 | TTTATA |
|  |  |  |  | 21 | R | 1029 | 1034 | 9.889837 | 9.889837 | 0.000000 | TTTATT |
|  |  |  |  | 22 | R | 1043 | 1048 | 9.889837 | 9.889837 | 0.000000 | TTTATT |
|  |  |  |  | 23 | R | 1047 | 1052 | 7.002311 | 9.889837 | 2.887525 | TTTATA |
|  |  |  |  | 24 | R | 1100 | 1105 | 7.002311 | 9.889837 | 2.887525 | TTTATG |
|  |  |  |  | 25 | R | 1131 | 1136 | 9.889837 | 9.889837 | 0.000000 | TTTATT |

## Common TFBSs for AIModules vs Genomatix’s MatInspector

The results from the crude TFBS search from AIModules and Genomatix’s MatInspector were high in number, so we used a python script to find matches in TF families. The results are shown in the tables below for both cathepsins and IL-10.

Table S 12 **Cathepsins.** Common TFBSs 1

| **Homo_sapiens_cathepsin_V_transcript_variant-1_promoter** |  |
| --- | --- |
| **MatInspector TFBS** | AIModules TFBS |
| V$AHRARNT.01 | MA0006.1 Ahr::Arnt |
| V$E2F6.01, V$E2F3.01, V$E2F2.01, V$E2F1.01, V$E2F6.01, V$E2F4.01, V$E2F4.01, | MA0471.2 E2F6 |
| V$EGR1.04, V$EGR1.04, V$EGR1.04, V$EGR1.04, V$EGR1.02 | MA0162.4 EGR1 |
| V$FOXP2.01 | MA0481.3 FOXP1 |
| V$GCM1.01, V$GCM1.02 | MA0767.1 GCM2 |
| V$MAZ.03, V$MAZ.03, V$MAZR.01, V$MAZ.01 | MA1522.1 MAZ |
| V$NFAT5.02 | MA0606.1 NFAT5, MA0624.1 NFATC1, MA0152.1 NFATC2, MA0625.1 NFATC3, MA1525.1 NFATC4 |
| V$NKX31.03, V$NKX31.03, V$NKX31.01 | MA1645.1 NKX2-2, MA0063.2 NKX2-5, MA0673.1 NKX2-8, MA0124.2 Nkx3-1, MA0122.3 Nkx3-2, MA0674.1 NKX6-1 |
| V$RFX1.01 | MA1554.1 RFX7 |
| V$NRF1.02, V$NRF1.01 | MA0506.1 NRF1 |
| V$SPIB.01, V$SP1.03, V$SP1.01 | MA0746.2 SP3, MA0747.1 SP8, MA1564.1 SP9 |
| V$TCFAP2B.01, V$TCFAP2B.01, V$CTCF.01, V$TCFCP2L1.01, V$TCFAP2E.01, V$CTCF.01 | MA1648.1 TCF12(var.2), MA0522.3 TCF3, MA0632.2 TCFL5 |
| V$ZFP57.01 | MA1583.1 ZFP57 |
| V$ZKSCAN3.01 | MA1585.1 ZKSCAN1 |
| V$ZNF282.01, V$ZNF263.01, V$ZNF263.01, V$ZNF219.01, V$ZNF263.02, V$ZNF444.01 | MA1653.1 ZNF148, MA0528.2 ZNF263, MA1630.1 Znf281, MA1655.1 ZNF341, MA1601.1 ZNF75D |

Table S 13 **Cathepsins.** Common TFBSs 2

| **Bos_taurus_cathepsin-Z_promoter** |  |
| --- | --- |
| **MatInspector** *TFBS* | *AIModules TFBS* |
| *V$AHRARNT.02, V$AHRARNT.01* | *MA0006.1 Ahr::Arnt, MA0259.1 ARNT::HIF1A, MA1464.1 ARNT2, MA0603.1 Arntl* |
| *V$CEBPE.02* | *MA0837.1 CEBPE* |
| *V$E2F1.01, V$E2F1.01, V$E2F4.01, V$E2F4_DP1.01, V$E2F4_DP1.01, V$E2F1.01, V$E2F4.01, V$E2F2.01, V$E2F2.01, V$E2F2.01, V$E2F4.01, V$E2F1.01* | *MA0471.2 E2F6* |
| *V$EBF1.01, V$EBF1.01* | *MA0154.4 EBF1, MA1604.1 Ebf2, MA1637.1 EBF3* |
| *V$ETV1.02, V$ETV1.02* | *MA0761.2 ETV1, MA0764.2 ETV4, MA0765.2 ETV5* |
| *V$GATA1.03, V$GATA.01, V$GATA3.02* | *MA0035.4 GATA1, MA0036.3 GATA2, MA0037.3 GATA3, MA0766.2 GATA5* |
| *V$GCM1.02, V$GCM1.02* | *MA0767.1 GCM2* |
| *V$HES1.01* | *MA1099.2 HES1, MA0616.2 HES2* |
| *V$PBX_HOXA9.01, V$MEIS1B_HOXA9.01* | *MA1497.1 HOXA6, MA0594.2 HOXA9* |
| *V$HOXB4.02, V$HOXB4.02* | *MA1500.1 HOXB6* |
| *V$MAZR.01, V$MAZR.01, V$MAZR.01, V$MAZR.01, V$MAZR.01, V$MAZR.01* | *MA1522.1 MAZ* |
| *V$PBX1_MEIS1.02, V$MEIS1B_HOXA9.01* | *MA0498.2 MEIS1, MA0774.1 MEIS2, MA0775.1 MEIS3* |
| *V$MYCMAX.03* | *MA0147.3 MYC* |
| *V$MZF1.01* | *MA0056.2 MZF1* |
| *V$NFATC1.01, V$NFATC1.01* | *MA0152.1 NFATC2* |
| *V$RFX4.01, V$RFX2.01* | *MA0509.2 RFX1, MA0798.2 RFX3* |
| *V$OSR2.01* | *MA1646.1 OSR2* |
| *V$RFX4.01, V$RFX2.01* | *s. rfx* |
| *V$TCF11MAFG.01, V$CTCF.01, V$CTCF.01, V$CTCF.01, V$CTCF.01, V$TCFAP2A.01, V$TCFAP2A.01, V$TCFAP2A.02, V$TCFAP2B.01* | *MA1648.1 TCF12(var.2), MA0522.3 TCF3, MA0830.2 TCF4, MA0769.2 TCF7, MA0632.2 TCFL5* |
| *V$VDR_RXR.05, V$VDR_RXR.04, V$VDR_RXR.01* | *MA0693.2 VDR* |
| *V$ZBTB7.03, V$ZBTB7.03, V$ZBTB7.03, V$ZBTB7.03* | *MA1649.1 ZBTB12, MA1650.1 ZBTB14* |
| *V$ZNF345.01, V$ZNF217.01, V$ZNF263.01, V$ZNF263.02, V$ZNF76_143.01, V$ZNF76_143.01, V$ZNF219.01, V$ZNF444.01, V$ZNF704.01, V$ZNF704.01, V$ZNF219.01, V$ZNF704.01, V$ZNF704.01, V$ZNF263.02* | *MA1653.1 ZNF148, MA0528.2 ZNF263* |

Table S 14 **Cathepsins.** Common TFBSs 3

| **Mus_musculus_cathepsin-F-transcript-variant-X1_promoter_576-1076** |  |
| --- | --- |
| **MatInspector** *TFBS* | *AIModules TFBS* |
| *V$BATF.01* | *MA1634.1 BATF, MA0462.2 BATF::JUN* |
| *V$JUNB.01* | *MA0462.2 BATF::JUN , MA1132.1 JUN::JUNB, MA0490.2 JUNB* |
| *V$BATF.01* | *MA1634.1 BATF, MA0462.2 BATF::JUN* |
| *V$RORA.01* | *MA0071.1 RORA* |
| *V$EN1.02* | *MA0027.2 EN1* |
| *V$ESRRA.02, V$ESRRA.01, V$ESRRA.03* | *MA0592.3 ESRRA* |
| *V$JUNB.01* | *s. jun* |
| *V$JUNB.01* | *s.jun* |
| *V$JUNB.01* | *s. jun* |
| *V$FOXP1_ES.01* | *MA0047.3 FOXA2, MA1683.1 FOXA3, MA0042.2 FOXI1, MA0614.1 Foxj2, MA1103.2 FOXK2, MA0033.2 FOXL1, MA1489.1 FOXN3, MA0157.2 FOXO3, MA0848.1 FOXO4, MA0849.1 FOXO6, MA0481.3 FOXP1, MA0593.1 FOXP2, MA0850.1 FOXP3* |
| *V$MEIS1B_HOXA9.01, V$MEIS1B_HOXA9.01, V$HOXC6.01, V$HOXC9.01, V$HOXC8.01* | *MA1495.1 HOXA1, MA0899.1 HOXA10, MA0900.2 HOXA2, MA1496.1 HOXA4, MA0158.2 HOXA5, MA1497.1 HOXA6, MA1498.1 HOXA7, MA0594.2 HOXA9, MA0902.2 HOXB2, MA0903.1 HOXB3, MA1499.1 HOXB4, MA0904.2 HOXB5, MA1500.1 HOXB6, MA1501.1 HOXB7, MA1502.1 HOXB8, MA0905.1 HOXC10, MA1504.1 HOXC4, MA1505.1 HOXC8, MA0912.2 HOXD3, MA1507.1 HOXD4, MA0910.2 HOXD8, MA0913.2 HOXD9* |
| *V$ISL2.01* | *MA0914.1 ISL2* |
| *V$ISX.01* | *MA0654.1 ISX* |
| *V$KLF12.01, V$KLF12.01, V$EKLF.01, V$KLF2.01, V$KKLF.01, V$GKLF.02* | *MA1512.1 KLF11* |
| *V$LMX1A.02* | *MA0702.2 LMX1A* |
| *V$LMX1B.01, V$LMX1B.01* | *MA0703.2 LMX1B* |
| *V$MEIS1B_HOXA9.01, V$MEIS1.02, V$PBX1_MEIS1.02, V$MEIS1B_HOXA9.01* | *MA0498.2 MEIS1, MA0775.1 MEIS3* |
| *V$MSX1.01* | *MA0666.1 MSX1, MA0708.1 MSX2, MA0709.1 Msx3* |
| *V$NKX25.05, V$NKX29.01, V$NKX12.01, V$NKX61.01, V$NKX12.01, V$NKX61.03, V$NKX25.02* | *MA0063.2 NKX2-5, MA0673.1 NKX2-8, MA0674.1 NKX6-1, MA0675.1 NKX6-2, MA1530.1 NKX6-3* |
| *V$RFX5.01* | *MA1554.1 RFX7* |
| *V$OSR2.01* | *MA1646.1 OSR2* |
| *V$PAX6.03, V$PAX6.03, V$PAX4.02, V$PAX4.02* | *MA0068.2 PAX4* |
| *V$PRDM14.01, V$PRDM4.01, V$PRDM15.01* | *MA0508.3 PRDM1, MA1647.1 PRDM4* |
| *V$PARAXIS.01* | *MA0718.1 RAX, MA0717.1 RAX2* |
| *V$SNAI2.01* | *MA1558.1 SNAI1, MA0745.2 SNAI2, MA1559.1 SNAI3* |
| *V$TEAD4.01* | *MA0808.1 TEAD3, MA0809.2 TEAD4* |
| *V$ZNF771.01, V$ZNF217.01, V$ZNF263.01* | *MA1593.1 ZNF317, MA1655.1 ZNF341, MA1657.1 ZNF652* |

Table S 15 **IL10.** Common TFBSs 1

| **X73536.1_H.sapiens_promoter_region_of_human_IL-10_gene** |  |
| --- | --- |
| **MatInspector** *TFBS* | *AIModules TFBS* |
| *V$BARX2.01, V$BARX2.02* | *MA0875.1 BARX1, MA0875.1 BARX2* |
| *V$CEBPE.02, V$CEBPE.01, V$CEBPB.01, V$CEBPB.01, V$CEBPE_ATF4.02, V$CEBPE.02, V$CEBPE.02* | *MA0102.4 CEBPA, MA0466.2 CEBPB, MA0836.2 CEBPD, MA0837.1 CEBPE* |
| *V$CREB.02* | *MA0638.1 CREB3, MA0608.1 Creb3l2, MA1474.1 CREB3L4* |
| *V$RORA2.01, V$RORA.01* | *MA0071.1 RORA* |
| *V$EN1.01* | *MA0027.2 EN1, MA0642.1 EN2* |
| *V$ESRRA.02* | *MA0592.3 ESRRA* |
| *V$FOXA1.01* | *MA0148.4 FOXA1, MA0047.3 FOXA2, MA1683.1 FOXA3, MA1606.1 Foxf1, MA0479.1 FOXH1, MA0042.2 FOXI1, MA1103.2 FOXK2, MA1489.1 FOXN3, MA0157.2 FOXO3, MA0848.1 FOXO4, MA0481.3 FOXP1, MA0850.1 FOXP3* |
| *V$GBX1.01* | *MA0889.1 GBX1, MA0890.1 GBX2* |
| *V$HOXA1.01* | *MA1495.1 HOXA1, MA0899.1 HOXA10, MA0900.2 HOXA2, MA1496.1 HOXA4, MA0158.2 HOXA5, MA1497.1 HOXA6, MA1498.1 HOXA7, MA0594.2 HOXA9* |
| *V$HOXB3.01, V$HOXB3.01, V$HOXB8.01, V$HOXB3.01, V$HOXB4.01* | *MA0902.2 HOXB2, MA0903.1 HOXB3, MA1499.1 HOXB4, MA0904.2 HOXB5, MA1500.1 HOXB6, MA1501.1 HOXB7, MA1502.1 HOXB8* |
| *V$HOXC4.01, V$HOXC13.02, V$HOXC13.01, V$HOXC4.01, V$HOXC9.01* | *MA0905.1 HOXC10, MA1504.1 HOXC4, MA1505.1 HOXC8* |
| *V$INSM1.01, V$INSM1.01* | *MA0155.1 INSM1* |
| *V$ISX.01* | *MA0654.1 ISX* |
| *V$EKLF.01, V$KLF2.01, V$GKLF.01, V$KLF2.01, V$KLF6.01, V$GKLF.01, V$GKLF.02* | *MA1515.1 KLF2, MA0039.4 KLF4, MA0599.1 KLF5, MA1517.1 KLF6* |
| *V$LHX2.01, V$LHX3.02, V$LHX3.02, V$LHX6.01* | *MA1518.1 LHX1, MA0700.2 LHX2, MA0704.1 Lhx4, MA1519.1 LHX5, MA0658.1 LHX6, MA0705.1 Lhx8, MA0701.2 LHX9* |
| *V$LMX1A.02, V$LMX1A.02* | *MA0702.2 LMX1A* |
| *V$MAX.03* | *MA0058.3 MAX* |
| *V$MEF2A.01* | *MA0052.4 MEF2A, MA0660.1 MEF2B, MA0497.1 MEF2C, MA0773.1 MEF2D* |
| *V$MNT.01* | *MA0825.1 MNT* |
| *V$MSX.01, V$MSX1.01* | *MA0666.1 MSX1, MA0708.1 MSX2, MA0709.1 Msx3* |
| *V$MYBL1.02* | *MA0100.3 MYB* |
| *V$NKX25.02, V$NKX12.01, V$NKX61.02, V$NKX25.02, V$NKX31.01, V$NKX63.01* | *MA0672.1 NKX2-3* |
| *V$MYOD.02, V$MYOD.02* | *MA0499.2 MYOD1* |
| *V$MYOGENIN.03, V$MYOGENIN.03* | *MA0500.2 MYOG* |
| *V$NFAT.01, V$NFAT5.02, V$NFAT5.01* | *MA0606.1 NFAT5, MA0624.1 NFATC1, MA0152.1 NFATC2, MA0625.1 NFATC3, MA1525.1 NFATC4* |
| *V$NR2F6.01* | *MA0017.2 NR2F1, MA1111.1 NR2F2* |
| *V$RFX1.01, V$RFX3.03, V$RFX1.01* | *MA1554.1 RFX7* |
| *V$PRDM14.01, V$PRDM14.01, V$PRDM14.01* | *MA1647.1 PRDM4* |
| *V$RBPJK.02* | *MA1116.1 RBPJ* |
| *V$STAT.01, V$STAT6.01, V$STAT3.02, V$STAT6.01, V$STAT6.01, V$STAT3.02, V$STAT.01* | *MA0137.3 STAT1, MA0144.2 STAT3, MA0518.1 Stat4, MA0519.1 Stat5a::Stat5b* |
| *V$TCF21.01, V$TCF21.01, V$TCF21.01* | *MA0521.1 Tcf12, MA1648.1 TCF12(var.2), MA0832.1 Tcf21, MA0522.3 TCF3, MA0769.2 TCF7* |
| *V$TEAD4.01* | *MA0090.3 TEAD1, MA1121.1 TEAD2, MA0808.1 TEAD3, MA0809.2 TEAD4* |
| *V$VDR_RXR.06, V$VDR_RXR.05, V$VDR_RXR.06* | *MA0693.2 VDR* |
| *V$ZBTB7.01* | *MA1649.1 ZBTB12, MA1579.1 ZBTB26* |
| *V$ZNF444.01, V$ZNF282.01, V$ZNF300.01, V$ZNF282.01* | *MA0528.2 ZNF263, MA1630.1 Znf281, MA1593.1 ZNF317, MA1655.1 ZNF341, MA1125.1 ZNF384* |
| *O$ZSCAN4.01, V$ZSCAN10.01* | *MA1602.1 ZSCAN29* |

Table S 16 **IL10.** Common TFBSs 2

| **AY486432.1_Macaca-mulatta_interleukin-10-(IL-10)_gene_promoter_region** |  |
| --- | --- |
| **MatInspector** *TFBS* | *AIModules TFBS* |
| *V$BARX2.01, V$BARX2.02* | *MA0875.1 BARX1, MA1471.1 BARX2* |
| *V$CEBPB.01, V$CEBPE_ATF4.01, V$CEBPE.02, V$CEBPE.01, V$CEBPB.01, V$CEBPB.01, V$CEBPE_ATF4.02, V$CEBPE.02, V$CEBPE.02* | *MA0102.4 CEBPA, MA0466.2 CEBPB, MA0836.2 CEBPD, MA0837.1 CEBPE, MA1636.1 CEBPG(var.2)* |
| *V$CREB1.02, V$CREB.02* | *MA0018.4 CREB1, MA0638.1 CREB3, MA0608.1 Creb3l2, MA1474.1 CREB3L4, MA1475.1 CREB3L4(var.2), MA0840.1 Creb5* |
| *V$RORA2.01, V$RORA.01* | *MA0071.1 RORA* |
| *V$E2F1.01, V$E2F7.02* | *MA0471.2 E2F6* |
| *V$EN1.01, V$EN1.01* | *MA0027.2 EN1, MA0642.1 EN2* |
| *V$ERG.02* | *MA0474.2 ERG* |
| *V$ESRRA.02, V$ESRRA.04* | *MA0592.3 ESRRA* |
| *V$ETS1.01* | *MA0098.3 ETS1, MA1484.1 ETS2* |
| *V$ETV1.02* | *MA0761.2 ETV1, MA0763.1 ETV3, MA0764.2 ETV4, MA0765.2 ETV5, MA0645.1 ETV6* |
| *V$JUNDM2.01* | *MA0488.1 JUN, MA1132.1 JUN::JUNB, MA1133.1 JUN::JUNB(var.2), MA0492.1 JUND(var.2)* |
| *V$GATA1.01* | *MA0035.4 GATA1, MA0037.3 GATA3, MA0482.2 GATA4* |
| *V$GBX1.01* | *MA0889.1 GBX1, MA0890.1 GBX2* |
| *V$GFI1.01, V$GFI1.02* | *MA0038.2 GFI1, MA0483.1 Gfi1b* |
| *V$GRHL1.01, V$GRHL2.01* | *MA1105.2 GRHL2* |
| *V$HMBOX.01* | *MA0895.1 HMBOX1* |
| *V$HOXB6.01, V$HOXD13.01, V$HOX1-3.01, V$HOXB8.01, V$HOXC4.01, V$HOXC13.02, V$PHOX2.01, V$HOXA1.01, V$HOXC13.01, V$HOXB3.01, V$MEIS1A_HOXA9.01, V$HOXB9.02* | *MA1495.1 HOXA1, MA0899.1 HOXA10, MA0900.2 HOXA2, MA1496.1 HOXA4, MA0158.2 HOXA5, MA1497.1 HOXA6, MA1498.1 HOXA7, MA0594.2 HOXA9, MA0901.2 HOXB13, MA0902.2 HOXB2, MA0903.1 HOXB3, MA1499.1 HOXB4, MA0904.2 HOXB5, MA1500.1 HOXB6, MA1501.1 HOXB7, MA1502.1 HOXB8, MA0905.1 HOXC10, MA1504.1 HOXC4, MA1505.1 HOXC8, MA1507.1 HOXD4, MA0910.2 HOXD8, MA0913.2 HOXD9* |
| *V$INSM1.01, V$INSM1.01, V$INSM1.01* | *MA0155.1 INSM1* |
| *V$ISX.01* | *MA0654.1 ISX* |
| *V$JUNDM2.01* | *MA0488.1 JUN, MA1132.1 JUN::JUNB, MA1133.1 JUN::JUNB(var.2), MA0492.1 JUND(var.2)* |
| *V$GKLF.01, V$KKLF.01, V$EKLF.01, V$KLF2.01, V$GKLF.01, V$EKLF.01, V$KLF2.01, V$KLF6.01, V$GKLF.01, V$GKLF.02* | *MA1515.1 KLF2, MA0039.4 KLF4, MA0599.1 KLF5, MA1517.1 KLF6* |
| *V$LHX2.01, V$LHX3.02, V$LHX3.02, V$LHX6.01* | *MA1518.1 LHX1, MA0700.2 LHX2, MA0704.1 Lhx4, MA1519.1 LHX5, MA0658.1 LHX6, MA0705.1 Lhx8, MA0701.2 LHX9* |
| *V$LMX1A.02* | *MA0702.2 LMX1A* |
| *V$MEF2.01, V$MEF2A.01* | *MA0052.4 MEF2A, MA0660.1 MEF2B, MA0497.1 MEF2C, MA0773.1 MEF2D* |
| *V$MEIS1A_HOXA9.01* | *MA0498.2 MEIS1, MA0774.1 MEIS2, MA0775.1 MEIS3* |
| *V$MSX.01, V$MSX1.01* | *MA0666.1 MSX1, MA0708.1 MSX2, MA0709.1 Msx3* |
| *V$VMYB.01, V$MYBL1.02, V$MYBL1.02, V$CMYB.01* | *MA0100.3 MYB* |
| *V$NKX25.02, V$NKX25.02* | *MA0672.1 NKX2-3* |
| *V$MYOD.02* | *MA0499.2 MYOD1* |
| *V$MYOGENIN.03* | *MA0500.2 MYOG* |
| *V$MZF1.02, V$MZF1.02, V$MZF1.02, V$MZF1.01* | *MA0056.2 MZF1* |
| *V$NFAT.01, V$NFAT5.01, V$NFATC1.01, V$NFATC1.01, V$NFAT.01* | *MA0606.1 NFAT5, MA0624.1 NFATC1, MA0152.1 NFATC2 , MA0625.1 NFATC3, MA1525.1 NFATC4* |
| *V$NR2F6.01* | *MA0017.2 NR2F1, MA1111.1 NR2F2* |
| *V$RFX2.01, V$RFX1.01* | *MA1554.1 RFX7* |
| *V$OTX2.01* | *MA0711.1 OTX1, MA0712.2 OTX2* |
| *V$PRDM15.01, V$PRDM14.01, V$PRDM14.01, V$PRDM14.01* | *MA0508.3 PRDM1* |
| *V$PROX1.01, V$PROX1.01* | *MA0794.1 PROX1* |
| *V$SIX4.01, V$SIX2.02* | *MA1118.1 SIX1* |
| *V$SNAI3.01* | *MA1558.1 SNAI1, MA0745.2 SNAI2, MA1559.1 SNAI3* |
| *V$SRY.05* | *MA0084.1 SRY* |
| *V$STAT1.01, V$STAT.01, V$STAT6.01, V$STAT6.01, V$STAT6.01, V$STAT3.02, V$STAT.01* | *MA0137.3 STAT1, MA0144.2 STAT3, MA0518.1 Stat4, MA0519.1 Stat5a::Stat5b* |
| *V$TEAD4.01* | *MA0090.3 TEAD1, MA1121.1 TEAD2, MA0808.1 TEAD3, MA0809.2 TEAD4* |
| *V$TEF.01, V$TEF_HLF.01* | *MA0843.1 TEF* |
| *V$VDR_RXR.01, V$VDR_RXR.04, V$VDR_RXR.03, V$VDR_RXR.05, V$VDR_RXR.06* | *MA0693.2 VDR* |
| *V$YY2.01* | *MA0748.2 YY2* |
| *V$ZBTB3.01* | *MA1649.1 ZBTB12, MA1579.1 ZBTB26, MA1581.1 ZBTB6* |
| *V$ZKSCAN3.01* | *MA1585.1 ZKSCAN1* |
| *V$ZNF35.01, V$ZNF263.02, V$ZNF771.01, V$ZNF444.01, V$ZNF219.01, V$ZNF300.01, V$ZNF282.01* | *MA0528.2 ZNF263, MA1630.1 Znf281, MA1593.1 ZNF317, MA1655.1 ZNF341, MA1125.1 ZNF384* |
| *O$ZSCAN4.01, V$ZSCAN10.01* | *MA1602.1 ZSCAN29* |

Table S 17 **IL10.** Common TFBSs 3

| **AF121965.1_Mus-musculus_interleukin-10-(IL10)_gene_promoter_partial_sequence** |  |
| --- | --- |
| **MatInspector** *TFBS* | *AIModules TFBS* |
| *V$E2F7.02* | *MA0471.2 E2F6* |
| *V$ETV1.02* | *MA0761.2 ETV1, MA0764.2 ETV4, MA0765.2 ETV5* |
| *V$FOXO1.01, V$FOXP1_ES.01* | *MA0148.4 FOXA1, MA0047.3 FOXA2, MA1683.1 FOXA3, MA0845.1 FOXB1, MA0032.2 FOXC1, MA0846.1 FOXC2, MA0847.2 FOXD2, MA0041.1 Foxd3, MA0042.2 FOXI1, MA0614.1 Foxj2, MA1103.2 FOXK2, MA0033.2 FOXL1, MA0480.1 Foxo1, MA0848.1 FOXO4, MA0849.1 FOXO6, MA0481.3 FOXP1, MA0593.1 FOXP2, MA0850.1 FOXP3* |
| *V$HOXD10.01* | *MA1495.1 HOXA1, MA0594.2 HOXA9, MA1501.1 HOXB7* |
| *V$KLF6.01, V$IKLF.01, V$KLF2.01, V$GKLF.02* | *MA0741.1 KLF16, MA1515.1 KLF2, MA0039.4 KLF4, MA0599.1 KLF5* |
| *V$LHX6.02* | *MA1518.1 LHX1, MA0704.1 Lhx4, MA0705.1 Lhx8* |
| *V$SOX9.03* | *MA1563.1 SOX18* |
| *V$RFX5.01* | *MA1554.1 RFX7* |
| *V$PAX6.05, V$PAX3.02, V$PAX6.04* | *MA0014.3 PAX5* |
| *V$PLAGL1.02* | *MA1548.1 PLAGL2* |
| *V$PRDM4.01* | *MA1647.1 PRDM4* |
| *V$RBPJK.02* | *MA1116.1 RBPJ* |
| *V$SRY.05* | *MA0084.1 SRY* |
| *V$CTCF.01* | *MA0769.2 TCF7* |
| *V$TEAD4.01* | *MA1121.1 TEAD2, MA0808.1 TEAD3, MA0809.2 TEAD4* |
| *V$ZNF652.02, V$ZNF263.02, V$ZNF217.01, V$ZNF444.01* | *MA0528.2 ZNF263, MA1655.1 ZNF341, MA0116.1 Znf423, MA1601.1 ZNF75D* |

## Comparison of the different tools for TFBS discovery

### MotifMap (3)

MotifMap shows TFBSs for model organisms using not only databases of transcription factor binding motifs, but genome alignments and statistical approaches (see Figure S 1, Figure S 2 and Figure S 3) (4).


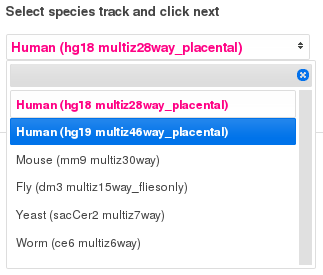


Figure S 1 **Model Species can be selected in MotifMap.**


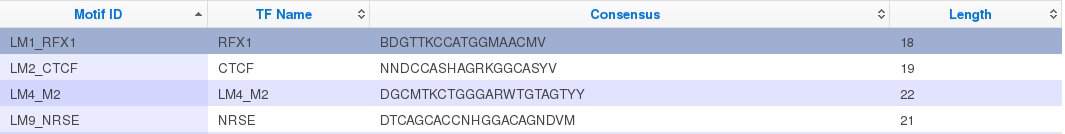


Figure S 2 **A motif from that model organism can be selected.**


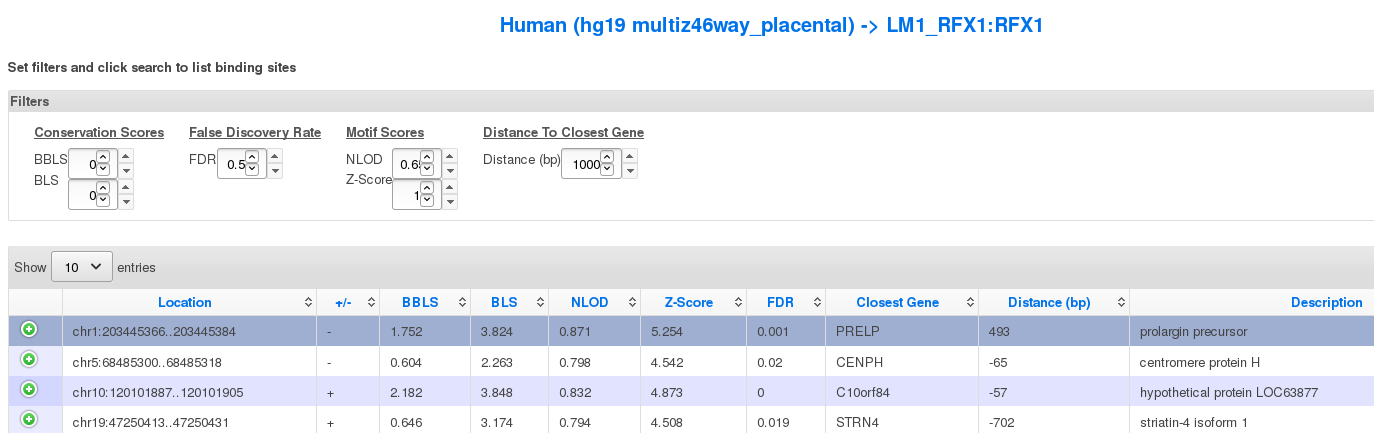


Figure S 3 **MotifMap searches for this motif in the model organism's genome.**

However, the tool does not support searches for common modules.

### Promo (5) (6)

Promo is using the TRANSFAC^®^ database to search for TFBSs for different organisms. In the first step you select the factors that should be considered. In the second step you can input DNA sequences to be searched for TFBSs, which are shown on the result page (see Figure S 4 to Figure S 9). Common Modules cannot be detected.


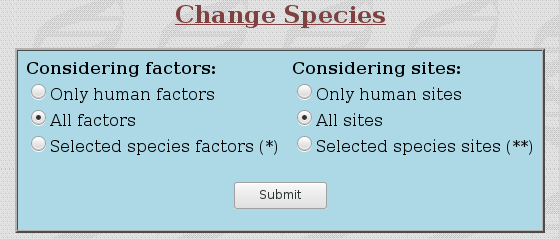


Figure S 4 **Step one:** What to consider for the analysis.


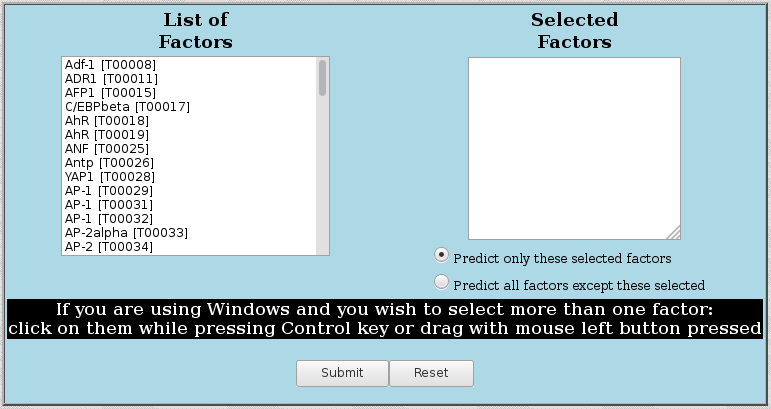


Figure S 5 **Matrices can also be directly selected for analysis.**


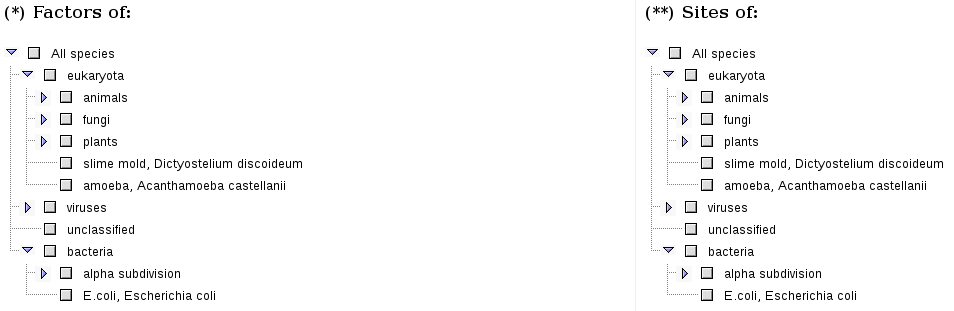


Figure S 6 **Factors and sites to consider can be selected via checkboxes.**


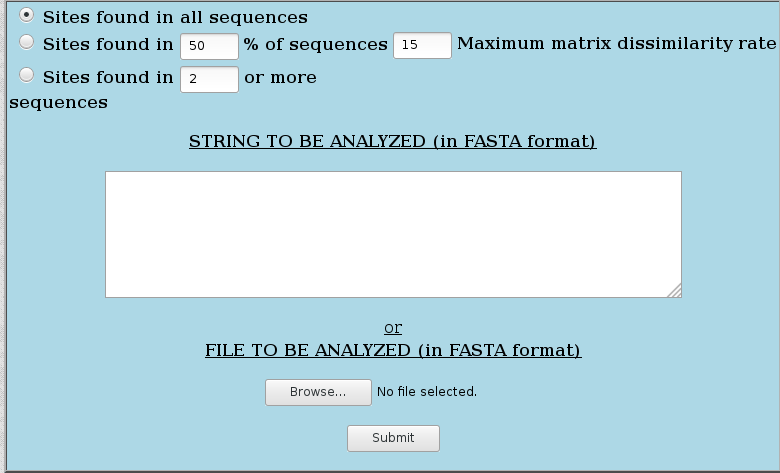


Figure S 7 **...or multiple sequences.**


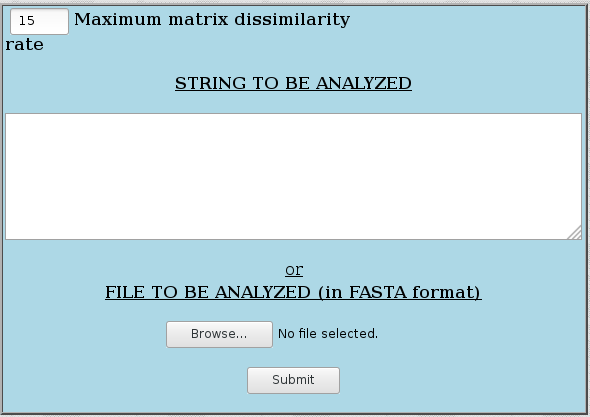


Figure S 8 **One sequence can be analyzed...**


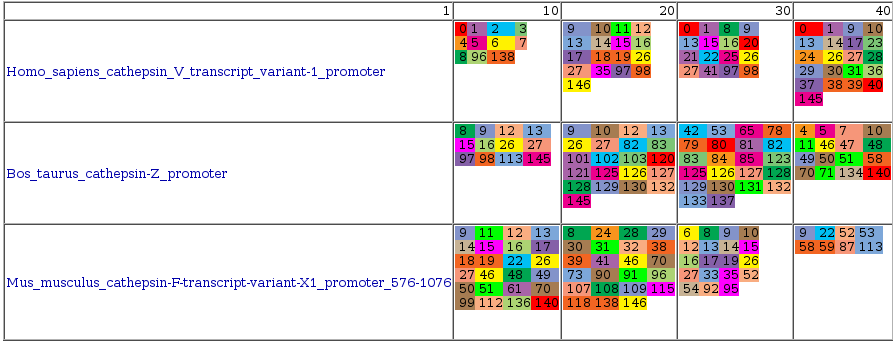


Figure S 9 **The result from Promo show color encoded TFBSs.**

### ModuleMaster (7)

According to its website ModuleMaster allows identification of cis-regulatory modules using promoter analysis and microarray expression data regression. However, the authors of this paper were not able to start the WebStart application ModuleMaster on both Linux Mint 64bit and Windows 10 64bit. As the originating institute discontinued bioinformatics research there was no support. Furthermore, besides not starting, the user is confronted with a certificate warning at application start.

The authors tried to start the WebStart App via

*$ javaws mm.jnlp*

and

*$ javaws http://www.ra.cs.uni-tuebingen.de/software/ModuleMaster/downloads/start.php?fn=mm.jnlp*

In both cases the error was
*Caused by: java.lang.NoClassDefFoundError: javax/xml/soap/SOAPException*

This error could not be resolved.

### Prodoric® (8)

Prodoric® (*PRO*caryot*ICD*atabase *O*f Gene *R*egulation) predicts TFBSs in prokaryotes using manually curated binding sites. This is done in the module *Virtual Footprint* (9) and in the submodule *Promoter Analysis.* There, the user can select a prokaryotic genome, then fixed matrices and begin the analysis (see Figure S 10).


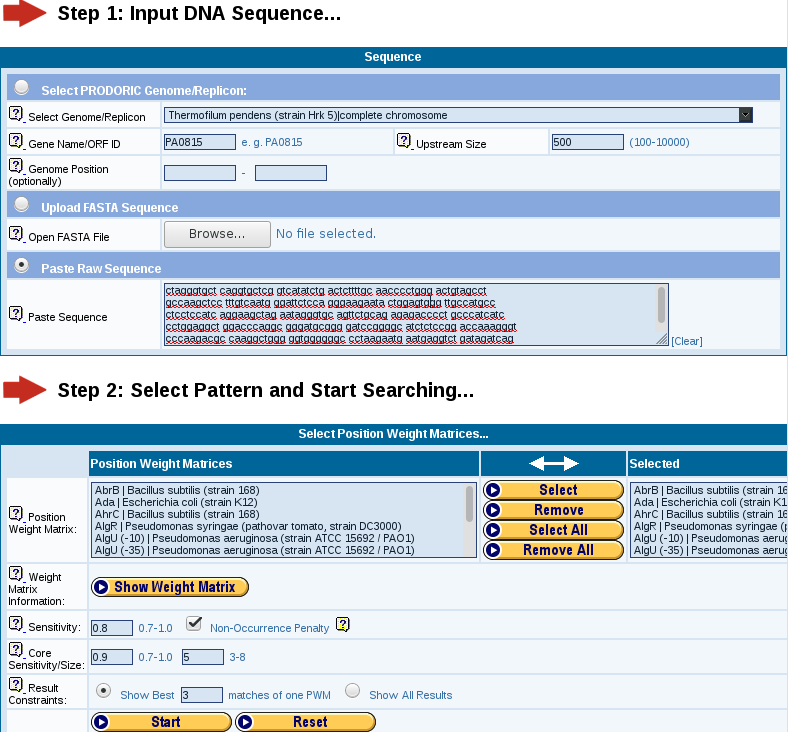


Figure S 10 **Prodoric.** Sequence and Matrix selection.

The result depicts found prokaryotic TFBSs (see Figure S 11).


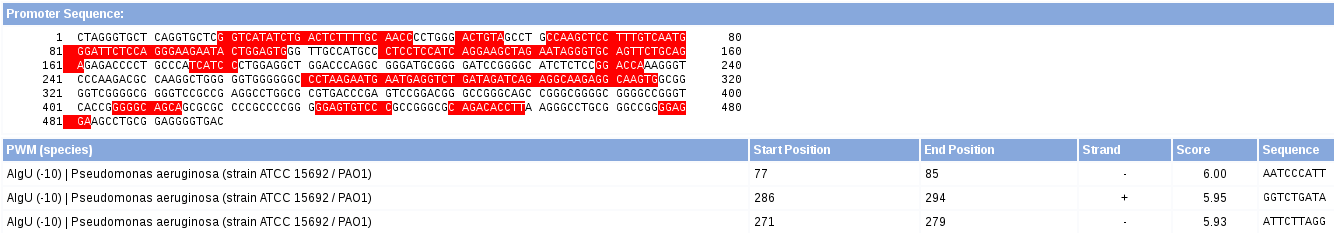


Figure S 11 **Prodoric.** Result of analysis. TFBSs are shown in a table.

Common Modules, however, cannot be detected.

### Softberry

Softberry, Inc. develops software tools for genomic research, amongst tools to search for functional and conserved TFBSs:

- PROMH(G): Promoter prediction using orthologous sequences (10)

- Nsite (11): Recognition of regulatory motifs (12) (13)

- NsiteH (14): Search for functional motifs conserved in orthologous sequences (13)

- NsiteM (15): Recognition of conserved regulatory motifs (12) (13)

All these tools produce text-style results without information about putative common modules. The user can insert one sequence at a time and another when comparing a sequence with an orthologous one.

### TAIR (16)

Tair (*T*he *A*rabidopsis *I*nformation *R*esource) (see Figure S 13) is a tool for promoter analysis, which allows for user input sequences. They are processed for TFBSs, which are common to at least three sequences. The TFBSs are constrained to 6-mers and the tool is made for the organism *A. thaliana*. Searches for common modules are not supported.


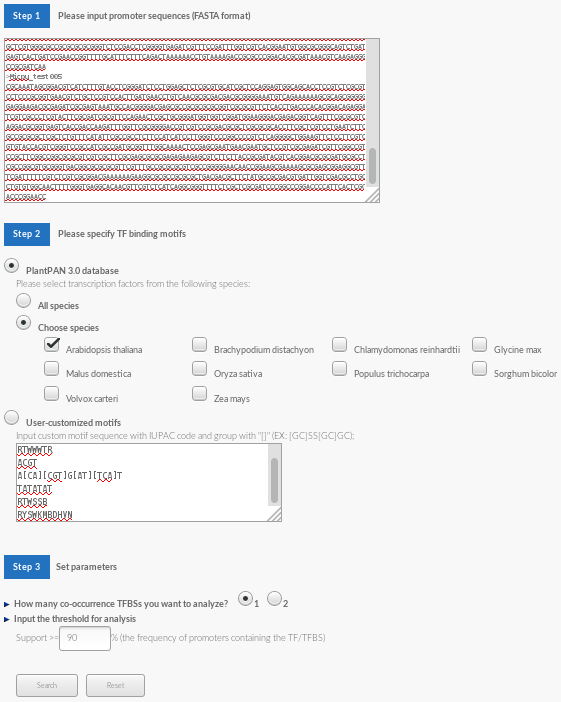


Figure S 12 **PlantPan 3.0.** The tool Gene Group Analysis allows for searching for TFBSs on a set of sequences.


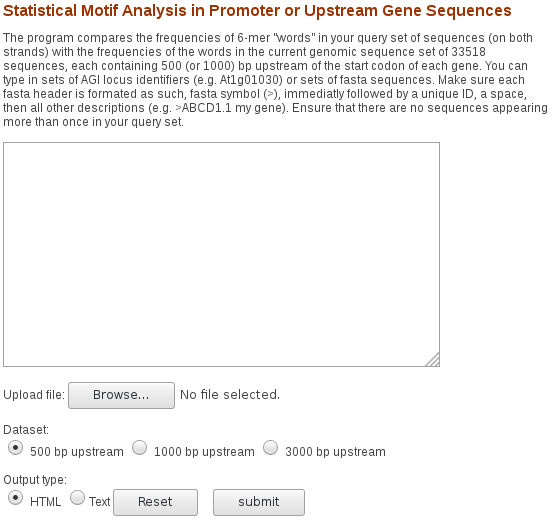


Figure S 13 **Tair.** A tool to search for 6-mer TFBSs from A. thaliana.

### PlantPan 3.0 (17)

PlantPan 3.0 (The *PlantP*romoter *A*nalysis *N*avigator) is a TFBS prediction tool for plant species. The user can input matrices as IUPAC code or can use predefined sets of matrices. The tool *Gene Group Analysis* (18) allows for scanning user provided sequences for TFBSs (see Figure S 12). Common Module discovery is not supported.

### TESS - Transcription Element Search System

TESS (19) (20; 21) (According to the Software License this sentence has to be included in this document: „This product includes software developed by CBIL at the Center for Bioinformatics at the University of Pennsylvania.“) was a website for TFBSs prediction. According to the website, the tool itself is not available anymore, as the author has left the group. The code for parts of the backend is provided and is used in AIModules for TFBS prediction.

### Genomatix

Genomatix (22) (Owned by Intrexon Bioinformatics Germany GmbH) is a company that offers commercial web services. After a free one-week trial period, it must be licensed for further use. Furthermore, the trial version lacks some features. Available tools:

- MatInspector: Search sequence(s) for transcription factor binding sites, restriction sites, etc.
- Common TFs: Search multiple sequences for common transcription factor binding sites
- MatDefine: Create user-defined matrix from a set of short sequences
- FastM: Build user-defined models, i.e. modules, with spacers between them
- ModelInspector: Search sequence(s) for promotor co-cited modules or user-defined modules

The tool ModelInspector searches for modules that are found in each user-input sequence, but does not show modules that are common to the user input sequences.

### TRANSFAC^®^ (23), (24)

TRANSFAC^®^ is another commercial product that can detect modules for eukaryotes. The *Match* algorithm can search for TFBSs and the *Composite Search* searches for modules (25). Genomes are available for eukaryotes, but sequences can be provided by the user as well. Matrices and modules are provided by TRANSFAC^®^ but custom ones can be inserted as well. There is a free version of TRANSFAC^®^ available that is outdated, from 2005 (26) and lacks functionalities (27). Furthermore, there is a trial access for TRANSFAC^®^ Professional available, which can be obtained via E-Mail (not tested by authors). The user may license TRANSFAC^®^ Professional. However, the service does not provide a functionality to search for common modules for the user input sequences.

### ConTraV3 (28), (29)

ConTraV3 is a web application that allows for TFBS searches. For that, model organisms can be chosen as well as a file with FASTA sequences. Matrices can be selected from the database or can be uploaded via a file upload (see Figure S 14). Common Module discovery is not supported.


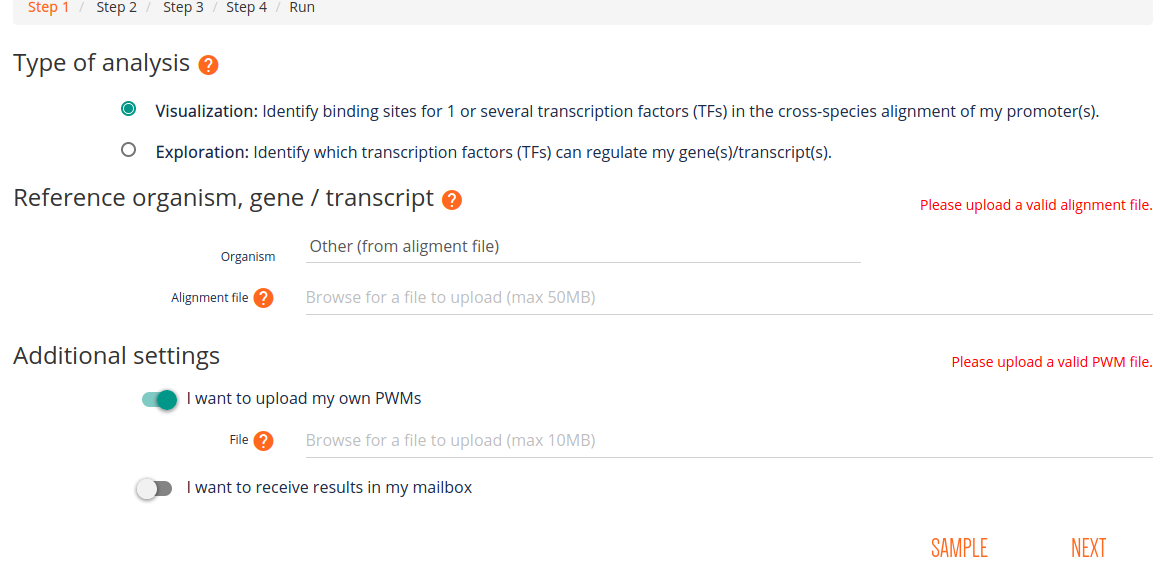


Figure S 14 **ConTraV3.** TFBSs can be searched for a user’s own sequences and with a user’s own matrices.

### CisBP (30; 31)

CisBP offers a very large database of eukaryotic matrices and genomes. With this service the user can predict TFBSs (see Figure S 15). Moreover, matrices may be downloaded for many species or TF-families. Module discovery is not supported.


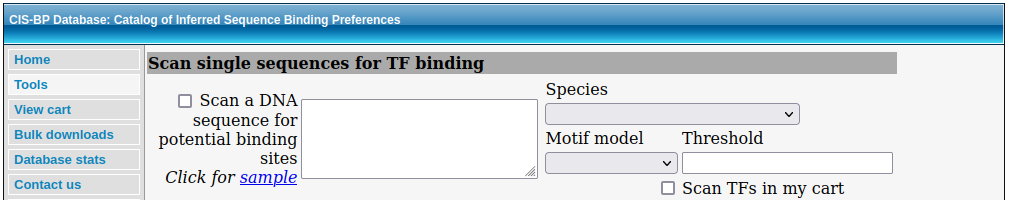


Figure S 15 **CisBP.** Prediction of TFBSs

### UniPROBE (32; 33)

With this service the user may search for eukaryotic TFBSs. However, the service does not work reliably, so that the analysis did not work during the test (33). Module searches are not supported.


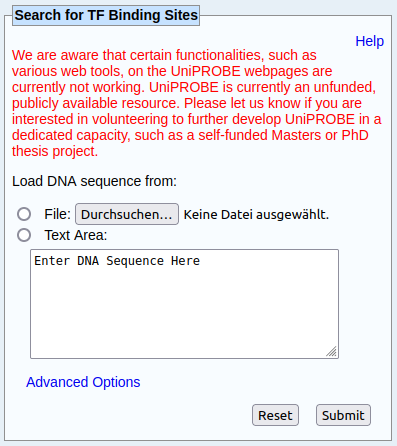


Figure S 16 **UniPROBE.** Search for eukaryotic TFBSs, however the search did not succeed due to an internal error (33)

### HOCOMOCO (34; 35)

HOMOCODO (HOmo sapiens COmprehensive MOdel COllection) is a collection of models for human and mouse. These models have their basis on ChiP-seq data. Via the “MoLoTool” the user may search for TFBSs (see Figure S 17). Module searches are not supported.


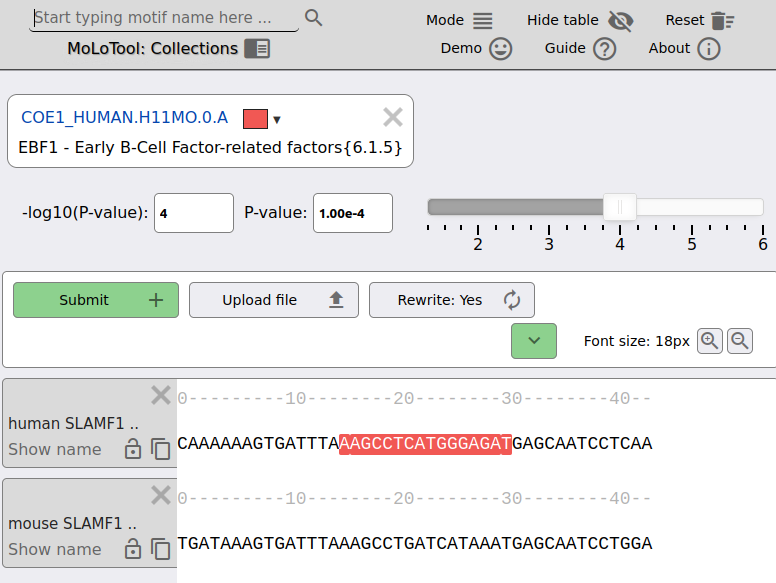


Figure S 17 **HOMOCOCO MoLoTool.** Search for TFBSs

### FlyFactorSurvey (36)

FlyFactorSurvey provides genomes and matrices to predict TFBSs for *Drosophila melanogaster* (see Figure S 18). However, module searches are not possible via this service.


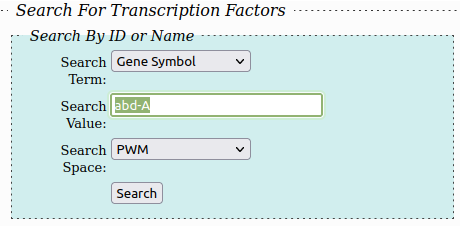


Figure S 18 **FlyFactorSurvey.** Search for TFBSs in Drosophila melanogaster

### MEME Suite (37; 38)

The MEME Suite offers many tools as web services. One of which is “MEME” that supports motif discovery in sequences (see Figure S 19). The suite, however, does not support module prediction.


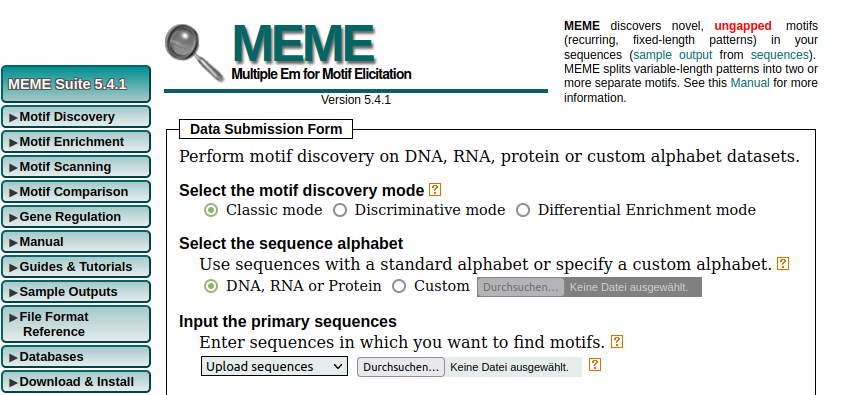


Figure S 19 **MEME.** Discovery of motifs on sequences

### YeTFaSCo (39; 40)

YeTFaSCO (The Yeast Transcription Factor Specificity Compendium) allows the search for TFBSs via matrices for *Saccharomyces cerevisiae* (see *Figure S 20*). The search for modules is not supported.

*
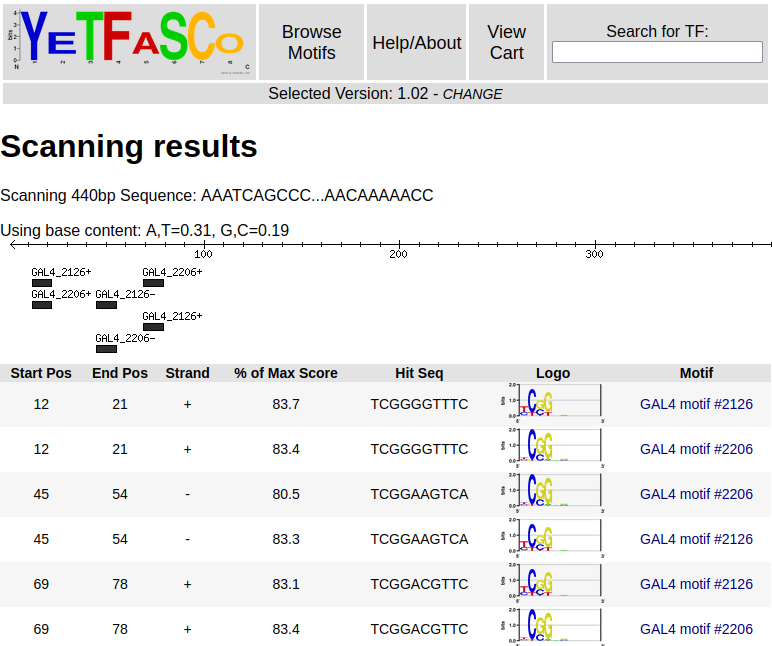
Figure S 20* ***YeTFaSCo.*** *TFBSs searches in S. cerevisiae with matrices*

## Used sequences

Below are sequences that are used for analyses in this paper.

### conTraV3 comparison

>AJ223836.1 Chionodraco hamatus mRNA for cathepsin

CAGACGGAGAGACAGACAGACGGAGAGACACACACACACACAGACACACACACACTCACAGACATGAGGT

CGGTGTTGCTGCTGCTGTGTATCTGGACATGCAGGAGCTCAGCGCTGATCAGGGTGCCTCTGAGGAAGGT

GCCCACGATCCGCTCTCAGCTCCGCTCTGAAGGTCTGCTGCAGGACTTCCTGGTGGAGAATCGGCCCGAC

ATGTTCAGTCGCCGCTACGCTCAGTGTTTCCCCGCCGGGACGCCCTCTCTGAGGCTGGGGCGCTCCAGTG

AGAAGATCTACAACTTCATGGACGCTCAGTATTACGGTGACATCGCGTTGGGGACCCCGGAGCAAAACTT

CTCTGTGGTCTTTGACACCGGATCGTCCGATCTGTGGGTGCCTTCGGCCTACTGCGTCACCGAAGCCTGT

GCGTTGCCAAAGCGCTTCAAGGCGTTTAAATCCACGTCTTTCCTCCATGACGGTCGGCAGTTTGGGATAA

ACTACGGATCAGGACACCTGCTGGGGGTCATGGGCCGAGACTACCTGATGGTGGCAGGTATGATGGTTAA

GAGGCAGGAGTTCCGGGAGTCGGTCTATGAGCCCGGCACTGCGTTTTTGAAGGCGAGGTTTGATGGCGTT

TTGGGCTTGGGTTACCCGGCCCTGGCAGAGATCCTGGGAAACCCCGTTTTCGACAACATGTTGGCGCAGA

ACTTGTTGGACAAGCCCATCTTCTCCTTCTACCTGAGCAGGAAATTAAATGGAAGCCCAGAGGGCGAGCT

GTTGCTGGGCGGAACAGACGAGAGGTTGTACGACTTACCAATCAACTGGCTCCCCGTGACTGCTAAGGCC

TACTGGCAGATCAAGATAGACAGCGTGGTGGTGCAGGGTGTGAATCCCTTCTGTCCCCACGGCTGTCAGG

CCATCGTCGACACGGGAACTTCTCTCATCACTGGACCCACTGACGACATCCTGGACATCCAGCAGCTGAT

CGGAGCCACGCCCACCAACTTCGGAGAGTTCATCGTCGACTGTGCCAGGTTGTCCAACTTTCCTCAACAT

CAACATTTCGTCCTCGGTGGGAAGGAGTACACTCTGACGTCCGACCAGTACATCAGGAAGGAGATGCTCG

GCGACAGGAAGTTATGCTTCAGTGGCTTCCAGGCCGTGGACATGATTTCCTCCGAAGGCCCCCTGTGGAT

TCTGGGAGATGTGTTTCTGACACAGTACTACAGCATTTTCGACAGAGGACAGGACCGGGTCGGCTTCGCC

ATCGCTAGATAACCAGTCGAAGTTTAACGATAATACGAGAGCTGAAAACTGCAATAAATTCTACACAATG

AC

### Sequences for poly adenylation site motif analysis

>NM_000600.5 Homo sapiens interleukin 6 (IL6), transcript variant 1, mRNA

ATTCTGCCCTCGAGCCCACCGGGAACGAAAGAGAAGCTCTATCTCCCCTCCAGGAGCCCAGCTATGAACT

CCTTCTCCACAAGCGCCTTCGGTCCAGTTGCCTTCTCCCTGGGGCTGCTCCTGGTGTTGCCTGCTGCCTT

CCCTGCCCCAGTACCCCCAGGAGAAGATTCCAAAGATGTAGCCGCCCCACACAGACAGCCACTCACCTCT

TCAGAACGAATTGACAAACAAATTCGGTACATCCTCGACGGCATCTCAGCCCTGAGAAAGGAGACATGTA

ACAAGAGTAACATGTGTGAAAGCAGCAAAGAGGCACTGGCAGAAAACAACCTGAACCTTCCAAAGATGGC

TGAAAAAGATGGATGCTTCCAATCTGGATTCAATGAGGAGACTTGCCTGGTGAAAATCATCACTGGTCTT

TTGGAGTTTGAGGTATACCTAGAGTACCTCCAGAACAGATTTGAGAGTAGTGAGGAACAAGCCAGAGCTG

TGCAGATGAGTACAAAAGTCCTGATCCAGTTCCTGCAGAAAAAGGCAAAGAATCTAGATGCAATAACCAC

CCCTGACCCAACCACAAATGCCAGCCTGCTGACGAAGCTGCAGGCACAGAACCAGTGGCTGCAGGACATG

ACAACTCATCTCATTCTGCGCAGCTTTAAGGAGTTCCTGCAGTCCAGCCTGAGGGCTCTTCGGCAAATGT

AGCATGGGCACCTCAGATTGTTGTTGTTAATGGGCATTCCTTCTTCTGGTCAGAAACCTGTCCACTGGGC

ACAGAACTTATGTTGTTCTCTATGGAGAACTAAAAGTATGAGCGTTAGGACACTATTTTAATTATTTTTA

ATTTATTAATATTTAAATATGTGAAGCTGAGTTAATTTATGTAAGTCATATTTATATTTTTAAGAAGTAC

CACTTGAAACATTTTATGTATTAGTTTTGAAATAATAATGGAAAGTGGCTATGCAGTTTGAATATCCTTT

GTTTCAGAGCCAGATCATTTCTTGGAAAGTGTAGGCTTACCTCAAATAAATGGCTAACTTATACATATTT

TTAAAGAAATATTTATATTGTATTTATATAATGTATAAATGGTTTTTATACCAATAAATGGCATTTTAAA

AAATTCA

>NM_000594.4 Homo sapiens tumor necrosis factor (TNF), mRNA

AGCAGACGCTCCCTCAGCAAGGACAGCAGAGGACCAGCTAAGAGGGAGAGAAGCAACTACAGACCCCCCC

TGAAAACAACCCTCAGACGCCACATCCCCTGACAAGCTGCCAGGCAGGTTCTCTTCCTCTCACATACTGA

CCCACGGCTCCACCCTCTCTCCCCTGGAAAGGACACCATGAGCACTGAAAGCATGATCCGGGACGTGGAG

CTGGCCGAGGAGGCGCTCCCCAAGAAGACAGGGGGGCCCCAGGGCTCCAGGCGGTGCTTGTTCCTCAGCC

TCTTCTCCTTCCTGATCGTGGCAGGCGCCACCACGCTCTTCTGCCTGCTGCACTTTGGAGTGATCGGCCC

CCAGAGGGAAGAGTTCCCCAGGGACCTCTCTCTAATCAGCCCTCTGGCCCAGGCAGTCAGATCATCTTCT

CGAACCCCGAGTGACAAGCCTGTAGCCCATGTTGTAGCAAACCCTCAAGCTGAGGGGCAGCTCCAGTGGC

TGAACCGCCGGGCCAATGCCCTCCTGGCCAATGGCGTGGAGCTGAGAGATAACCAGCTGGTGGTGCCATC

AGAGGGCCTGTACCTCATCTACTCCCAGGTCCTCTTCAAGGGCCAAGGCTGCCCCTCCACCCATGTGCTC

CTCACCCACACCATCAGCCGCATCGCCGTCTCCTACCAGACCAAGGTCAACCTCCTCTCTGCCATCAAGA

GCCCCTGCCAGAGGGAGACCCCAGAGGGGGCTGAGGCCAAGCCCTGGTATGAGCCCATCTATCTGGGAGG

GGTCTTCCAGCTGGAGAAGGGTGACCGACTCAGCGCTGAGATCAATCGGCCCGACTATCTCGACTTTGCC

GAGTCTGGGCAGGTCTACTTTGGGATCATTGCCCTGTGAGGAGGACGAACATCCAACCTTCCCAAACGCC

TCCCCTGCCCCAATCCCTTTATTACCCCCTCCTTCAGACACCCTCAACCTCTTCTGGCTCAAAAAGAGAA

TTGGGGGCTTAGGGTCGGAACCCAAGCTTAGAACTTTAAGCAACAAGACCACCACTTCGAAACCTGGGAT

TCAGGAATGTGTGGCCTGCACAGTGAAGTGCTGGCAACCACTAAGAATTCAAACTGGGGCCTCCAGAACT

CACTGGGGCCTACAGCTTTGATCCCTGACATCTGGAATCTGGAGACCAGGGAGCCTTTGGTTCTGGCCAG

AATGCTGCAGGACTTGAGAAGACCTCACCTAGAAATTGACACAAGTGGACCTTAGGCCTTCCTCTCTCCA

GATGTTTCCAGACTTCCTTGAGACACGGAGCCCAGCCCTCCCCATGGAGCCAGCTCCCTCTATTTATGTT

TGCACTTGTGATTATTTATTATTTATTTATTATTTATTTATTTACAGATGAATGTATTTATTTGGGAGAC

CGGGGTATCCTGGGGGACCCAATGTAGGAGCTGCCTTGGCTCAGACATGTTTTCCGTGAAAACGGAGCTG

AACAATAGGCTGTTCCCATGTAGCCCCCTGGCCTCTGTGCCTTCTTTTGATTATGTTTTTTAAAATATTT

ATCTGATTAAGTTGTCTAAACAATGCTGATTTGGTGACCAACTGTCACTCATTGCTGAGCCTCTGCTCCC

CAGGGGAGTTGTGTCTGTAATCGCCCTACTATTCAGTGGCGAGAAATAAAGTTTGCTTAGAAAAGAAA

>NM_020525.5 Homo sapiens interleukin 22 (IL22), mRNA

ACAAGCAGAATCTTCAGAACAGGTTCTCCTTCCCCAGTCACCAGTTGCTCGAGTTAGAATTGTCTGCAAT

GGCCGCCCTGCAGAAATCTGTGAGCTCTTTCCTTATGGGGACCCTGGCCACCAGCTGCCTCCTTCTCTTG

GCCCTCTTGGTACAGGGAGGAGCAGCTGCGCCCATCAGCTCCCACTGCAGGCTTGACAAGTCCAACTTCC

AGCAGCCCTATATCACCAACCGCACCTTCATGCTGGCTAAGGAGGCTAGCTTGGCTGATAACAACACAGA

CGTTCGTCTCATTGGGGAGAAACTGTTCCACGGAGTCAGTATGAGTGAGCGCTGCTATCTGATGAAGCAG

GTGCTGAACTTCACCCTTGAAGAAGTGCTGTTCCCTCAATCTGATAGGTTCCAGCCTTATATGCAGGAGG

TGGTGCCCTTCCTGGCCAGGCTCAGCAACAGGCTAAGCACATGTCATATTGAAGGTGATGACCTGCATAT

CCAGAGGAATGTGCAAAAGCTGAAGGACACAGTGAAAAAGCTTGGAGAGAGTGGAGAGATCAAAGCAATT

GGAGAACTGGATTTGCTGTTTATGTCTCTGAGAAATGCCTGCATTTGACCAGAGCAAAGCTGAAAAATGA

ATAACTAACCCCCTTTCCCTGCTAGAAATAACAATTAGATGCCCCAAAGCGATTTTTTTTAACCAAAAGG

AAGATGGGAAGCCAAACTCCATCATGATGGGTGGATTCCAAATGAACCCCTGCGTTAGTTACAAAGGAAA

CCAATGCCACTTTTGTTTATAAGACCAGAAGGTAGACTTTCTAAGCATAGATATTTATTGATAACATTTC

ATTGTAACTGGTGTTCTATACACAGAAAACAATTTATTTTTTAAATAATTGTCTTTTTCCATAAAAAAGA

TTACTTTCCATTCCTTTAGGGGAAAAAACCCCTAAATAGCTTCATGTTTCCATAATCAGTACTTTATATT

TATAAATGTATTTATTATTATTATAAGACTGCATTTTATTTATATCATTTTATTAATATGGATTTATTTA

TAGAAACATCATTCGATATTGCTACTTGAGTGTAAGGCTAATATTGATATTTATGACAATAATTATAGAG

CTATAACATGTTTATTTGACCTCAATAAACACTTGGATATCCTAA

### Analysis on how well AIModules recognizes motifs shown with the example of NFAT (41)

>NC_000067.6:131019345-131019844 Mus musculus strain C57BL/6J chromosome 1, GRCm38.p6 C57BL/6J, Mus musculus interleukin 10 (Il10) promoter
AAATCAGCCCTCTCGGGGTTTCCTTTGGGTAACTGAGTGCTAAGGTGACTTCCGAGTCAGCAAGAAATAT
CGGACGTTCAACCCAGGTTGAGTGGAGGAAACAATTATTTCTCAATCCTAATATGTTCTGGAATAGCCCA
TTTATCCACGTCATTATGACCTGGGAGTGCGTGAATGGAATCCACAGATGAGGGCCTCTGTACATAGAAC
AGCTGTCTGCCTCAGGAAATACAACTTTTAGTATTGAGAAGCTAAAAAGAAAAAAAAATTAAAAGAGAGG
TAGCCCATACTAAAAATAGCTGTAATGCAGAAGTTCATTCCGACCAGTTCTTTAGCGCTTACAATGCAAA
AAAAAGGGAAAGGAAAAAAAAAAAGAAAGAAATTAAACTCAAAAATTGCATGGTTTAGAAGAGGGAGGAG
GAGCCTGAATAACAAAAACCTTTGCCAGGAAGGCCCCACTGAGCCTTCAGTATAAAAGGGGGACCAAGAA
CAGGAGGTCT

>NC_000074.6:c82403252-82402576 Mus musculus strain C57BL/6J chromosome 8, GRCm38.p6 C57BL/6J, Mus musculus interleukin 15 (Il15), transcript variant 2 promoter
ACCCAAGGTCCTTTTCCGTTTCCTCCCGGTACAGTCTCTGTCCAGATCTCCTCCGGGCTTCTATGGGGAA
GCCAAACTGCCTCCCTGCAAGGCCAGTTGCTGTAGATGCAAGCAACCTGTGAACTCAGGCCAACTCCTTG
AAACTTCACAGAGGCAAAGGCATTCCAGGACACACAGAGGCTGTGGCCAACTGCCCAGGGGGAGGAGACT
GCTCTCTGCTCTCAGTTGCCCTTCAGGTTCTGCGCCCTGGGGACCTGGCAGTGGCAGAATCATGTGGGCA
CCTGGTAAGGTGGCCGGGACCATGTGTTCCTCCCGCAACCTCCCCAGGGTGTTGATCTCCGCCTCAGCTT
GGGCTCTTTCTCTTTCACTTTTCTGTTAGCTGGGGTTGGGACTCCCCGGCTGGAAAGCACTGGGGGAAAC
CGGGGAAACCCCAGCTGATTCGCTCCTTGTGCCTTGATTGCTCCCGCTGGCTGCTGCCCTGCATCCTGCA
CCCTTCAACCAGAACCCGATGGAGGTACAGAATGGGAGGTGGTAGTGCTGGTGGTGGTGGATCAACAATG
GAATTTTTTTTTTTTCCGAAAGCCTACGCCCCGGGCCCCTCCCAGCTCTGGCTCTGCTCAGGCACCCTTT
TCCCCTCCAGCTGCCGGCCAGGCCGCCCCGCCCTCT

>NC_000077.6:c53635202-53634702 Mus musculus strain C57BL/6J chromosome 11, GRCm38.p6 C57BL/6J, Mus musculus interleukin 13 (Il13) promoter
CATGCATTGCTTTGGTGATTTATCAGATACGTTTGTTGAAATTAAGAAGAAAAAAAGAAAAGAAAGAAAG
AAGAAAGGAAGGAAGGGAGGAAGGAAGAAAGAAAGAAAGGAGAAAACTTCCCCAATTCTCACAGCTAATA
GACTGATACTCACCTGCCCAAAGGGTGACAAGTACTTAGTGATCAAGGGGTCAGCATTGGGCTGGCTGCT
CAGGAGCTTGGGGCGGTCAGCGGGTGGAATTACTGGGGCGGAAGTTAGCTTTGCTGATGCCCACCGTGGA
AATAAACCACCCAGAACCTGGAAACCCTGTCCCAGACCCTTCTCAATAAATCCACTAAATCAGACTCTTT
CCTTTAGCGGCCACTGGATTTTCCAAAAAAGAAAAAAAAAAATTCAAGATGAGTAAAGATGTGGTTTTCA
GATAATGCCCAACAAAGCAGAGACCAGGGGTGAGGCGTCATCACTTTGGTTTATAAAAGCTGCTTCAACA
GGCTAAGGCC

### Genomatix’s solutions vs AIModules: here IL-10

>X73536.1_H.sapiens_promoter_region_of_human_IL-10_gene

AGCTTTCAGCAAGTGCAGACTACTCTTACCCACTTCCCCCAAGCACAGTTGGGGTGGGGGACAGCTGAAG

AGGTGGAAACATGTGCCTGAGAATCCTAATGAAATCGGGGTAAAGGAGCCTGGAACACATCCTGTGACCC

CGCCTGTCCTGTAGGAAGCCAGTCTCTGGAAAGTAAAATGGAAGGGCTGCTTGGGAACTTTGAGGATATT

TAGCCCACCCCCTCATTTTTACTTGGGGAAACTAAGGCCCAGAGACCTAAGGTGACTGCCTAAGTTAGCA

AGGAGAAGTCTTGGGTATTCATCCCAGGTTGGGGGGACCCAATTATTTCTCAATCCCATTGTATTCTGGA

ATGGGCAATTTGTCCACGTCACTGTGACCTAGGAACACGCGAATGAGAACCCACAGCTGAGGGCCTCTGC

GCACAGAACAGCTGTTCTCCCCAGGAAATCAACTTTTTTTAATTGAGAAGCTAAAAAATTATTCTAAGAG

AGGTAGCCCATCCTAAAAATAGCTGTAATGCAGAAGTTCATGTTCAACCAATCATTTTTGCTTACGATGC

AAAAATTGAAAACTAAGTTTATTAGAGAGGTTAGAGAAGGAGGAGCTCTAAGCAGAAAAAATCCTGTGCC

GGGAAACCTTGATTGTGGCTTTTTAATGAATGAAGAGGCCTCCCTGAGCTTACAATATAAAAGGGGGACA

GAGAGGTGAAGGTCTACACATCAGGGGCTTGCTCTTGCAAAACCAAACCACAAGACAGACTTGCAAAAGA

AGGCATGCACAGCTCAGCACTGC

>AY486432.1_Macaca-mulatta_interleukin-10-(IL-10)_gene_promoter_region

TTTGGGAAGGGGAAGTAGGGATAGGTAAGAGGAAAGTAAGGGACCTCCTATCCAGCCTCCATGGAATCCT

GACTTCTTTTCCCTGTTATTACAACTTCTTCCTCCCCATCTTTTAAACTTTAGACTCCCGCCACAGAAGC

TTACAACTAAAAGAAACTCTAAGGCCAATTTAATCCAAGGTTTCATTCTATGTGTTGGAGATGGCATAGA

GTAGGGTGAGGGAACCAAATTCTCAGTTGGCACTGGTGTACCCTTGTACAGGTGATGTAATCGCTCTGTG

CCTCAGTTTGCTCACTATAAAATAGAGATGGTAGGGGTCATGGTGAGCACTACCTGACTAGCATATAACA

AGCTTTCGGCAAGTGCAGACTACTCTTACCCACTTCCCCCAAGCACAGTTGGGGTGGGGGACACCTGGGA

CAGCTGAAGAGGAGGAAATGTGTGCCTGAGAATCCAAATGAAATCAGGGTAAAGGAGCCTGGAGCACATC

CTGTGACCCTGCCTGTCCTGTAGGAAGCCGGTCTCTGGAAGGTAAAATGGAAGAGCTGCTTGGGAGCTTT

GAGGATATTTAGCCCACCCCCTCATTTTTACTTGGGGAAACTAAGGCCCACAGACCTAAGGTGACTGTCT

AAGTTAGCAAGAAGAAGTCTTGGGTATTCACCCTGGGTTGGGGGGACCCAATTATTTCTCAATCCCATTG

TATTCTGGAATGGGCAATTTGTCCACGTCACTGTGACCTAGGAACACGCGAATGGGAACCCACAACTGCG

GGCCTCTGCGCACAGAACAGCTGTTCTCCCCAGGAAATCAACTTTTTTTAATTGAGAAGCTAAAAAATTA

TTCTAAGAGAGGTAGCCCATCCTAAAAATAGCTGTGCAGAAGTTCATGTTCAACCAATCCTTTTTGCTTA

CGATGCAAAATTTGAAAACTAAGTTTATTAGAGAGGTTAGAGAAGGAGGAGCTCTAAGCAGAAAAAATCC

TGTGCCGGGAAACCTGTGATTGTGGCTTTTTATGAATGAAGAGGCCTCCCTGAGCTTACAATATAAAAGG

GGGACAGAGAGGTGAAGGTCTACACATCAGGGGCTTGCTCTTGCAAAACCAAACCACAAGACAGACTTGC

AAAAGAAGGCATGCACAGCTCAGCACTGCTCTGTTGCCTGGTCCTCC

>AF121965.1_Mus-musculus_interleukin-10-(IL10)_gene_promoter_partial_sequence

TTCCTGTTCTACCAGCCCTGGTGTGGTAACCCTCTCCAATGGGGCAGGCTTGGAACCCTGTGCCAACGAA

GATCCTCCCCCGTACTGATGCAGGAAGGACAGCCCGGGAGTGTACCCTCTACATGGGTCTACTTTTATTT

AAGCAAACATTCCCTGGTCAACAGGACGTGTAGCATTGCCCCCCCCCCTTGGGTCACACAGAAAACAGGT

ACCAGGAGGACAAGTAGTTGCTTGCCCAGGGTACAGAATGAAAGGCAATAGGGGACTCTAGGCGAATGTT

CTTCCCACCCAAACTGAGGTAGTAGGAGAAGTCCCTACTGAAGGGAAGGTCCAGACATAATCAAAGGACT

ACCAGAGATCTCCCAAT

### Genomatix’s solutions vs AIModules: here cathepsins

>Homo_sapiens_cathepsin_V_transcript_variant-1_promoter

cttctaaagccctgatgcacccacccttaagtggaaggtaatgcttctgctgggggctgt

gcgttcttccactccgtcccacctttgccgtggactaaacaggagccactgaacgagagt

accctggctcccggccctgcagattttcaccaaaaactctaggactaagttaaagtaggg

actgaaataccacacagagctcgctgtaaaccacccgctcagcgcagtaagtcgggatag

tcagtagatctgagcccttgagggaggcgcctccccctcctccgcgcagcccccgggcgc

ccggcccagcttcccgcagccagggtgtattgaggtaggcgcgcccagacctgagacggg

ttgggactgggctgcgtcacgcgcgggctctaagcgcccggggccccgcccagtggccgg

cacagccaatcgcagcgcgggaaggcggtgggggcggggaaggccgcctggaaacttaaa

tcccgaggcgggcgaacctg

>Bos_taurus_cathepsin-Z_promoter

ctagggtgctcaggtgctcggtcatatctgactcttttgcaacccctgggactgtagcct

gccaagctcctttgtcaatgggattctccagggaagaatactggagtgggttgccatgcc

ctcctccatcaggaagctagaatagggtgcagttctgcagagagacccctgcccatcatc

cctggaggctggacccaggcgggatgcggggatccggggcatctctccggaccaaagggt

cccaagacgccaaggctgggggtggggggccctaagaatgaatgaggtctgatagatcag

aggcaagaggcaagtggcggggtcggggcggggtccgccgaggcctggcgcgtgacccga

gtccggacgggccgggcagccgggcggggcggggccgggtcaccgggggcagcagcgcgc

cccgccccgggggagtgtcccgccgggcgcagacaccttaagggcctgcgggccggggag

gaagcctgcggaggggtgac

> Mus_musculus_cathepsin-F-transcript-variant-X1_promoter_576-1076

gagccaccttgcaatgatcccctggtgtgcccactgcctgagacgaagaaaacagtggtg

agtgaagaggtcagctattgttggccctagctggcaaacaattggaatggtgggtcctgt

cgaggcattggttactcctgagataccaagttagagaccaagatgctctgaaagggtggg

tgttggggtgctgggtgtctgctgctggcctccctctttcaccaagttatgactgccttc

catgtggctctcctttgaggaacttctagctgctcttgccaggaccttgtttcaaactgt

cccaagaccgtcttctttctatgcctccagctctgcagttttgaagtcctggaagagcta

aaagaacacttgctgctgaggagggactgtagcccagtgaatgccaaggtcacaggtgct

gggattgctcaaggagactgggagagcccagaccatacttgagtcagaatccagccttaa

ttacttctctgttccgaccct

### Homo sapiens Immunoglobulin

> Homo sapiens chromosome 14, GRCh38.p13 Primary Assembly IGH 105588394-105588894

tggggtgggg ctcatggagt ggtgggtgtt ggactgagac tctgaccagg gacaggggga

tggggtcaca gccaagccac tccaccccta ccccatgcac acagcactca gagcccaggc

cccctcctca gagcccccac caaaatcctc tctaggggca ggggaaagag caagacatgt

cccccaccca gagcaggaac tggggtcagg gagctcaggg gactcagcca ctccatggca

gagccctgtt taatataact tgtgtctggg atggcctggg tcagaggccc tatctaagga

gcatgttcag aaactgtgtc gctgggatga gacagctggg tccaaccgca ggcccatggt

gcaggagctg tgtaaccttg gggctgtcac caggcctctc tgtgctgggt tcctccagtg

tagaggagag gcaggtacag cctgtcctcc tggggacatg gcatgagggc cgcgtcctca

cagcgcattc tgtgttccag c

> Homo sapiens chromosome 2, GRCh38.p13 Primary Assembly IGKC 88857683-88858183

gggctcaggg cctgctctgc agggaggttt tagcccagcc cagccaaagt aacccccggg

agcctgttat cccagcacag tcctggaaga ggcacagggg aaataaaagc ggacggaggc

tttccttgac tcagccgctg cctggtcttc ttcagacctg ttctgaattc taaactctga

gggggtcgga tgacgtggcc attctttgcc taaagcattg agtttactgc aaggtcagaa

aagcatgcaa agccctcaga atggctgcaa agagctccaa caaaacaatt tagaacttta

ttaaggaata gggggaagct aggaagaaac tcaaaacatc aagattttaa atacgcttct

tggtctcctt gctataatta tctgggataa gcatgctgtt ttctgtctgt ccctaacatg

ccctgtgatt atccgcaaac aacacaccca agggcagaac tttgttactt aaacaccatc

ctgtttgctt ctttcctcag g

> Homo sapiens chromosome 1, GRCh38.p13 Primary Assembly PIGR 206946466-206946966

tataaggggc atttttgtcc aggctaagta acctagggag tcggagggga ttccagagca

actggggata tgagaccaag gactacgaca gccactcctg ccacctgtgc cccatcagat

gatgtcaact tcaaatcaag cattgggcca ggtattttag agctaatacc gggctatatc

ctctacctgt agatttggta ttaccatccc tcttttccag atgaaaagaa ataggaaggt

gacttgccaa aggtcttgca gctagaaagc gacagaacag catcttcacg cttgacattc

tgtccctcat cctgaagctg caacgatgga ggattcccaa gtaacagagt ctccccaagg

tcaaaggaaa ccaaatggag ccagccagga aggccaaaat gaaaggaaag caagggatct

gtgagagtca catgaccctg gctggccacg gtgcctgtgg gagagtggcc ctttaagagc

ccaggtgtgg gtcaaacact g

### Drosophila melanogaster *oskar* (osk)

>NM_169248.4 Drosophila melanogaster oskar (osk), transcript variant A, mRNA

GGATCACTTTCCTCCAAGCGATGGCCGCAGTCACAAGTGAATTCCCCAGCAAACCGATCAGTTATACCAG

CACCAATACTTCCGCCAAAACCTATTATCTTAAGTCCGTGAAAAAGCGGGTGACCACGTGTTTCCAGCAG

TTGCGCGATAAACTCCAGTCATCCGGTTCCTTTCGCAAGAGTTCCTCCAGCTGCCTCAACCAGATCTTCG

TAAGGTCCGATTTCTCCGCATGCGGCGAACGCTTTCGGAAGATCTTCAAATCGGCGCGAAAAACCGAATT

GCCGGAGTTGTGGAAGGTGCCATTGGTGGCCCATGAACTCACCAGCCGGCAGAGCAGCCAGCAGTTGCAA

GTCGTAGCCAGACTCTTCTCGTCCACTCAGATTTCCACCAAGGAAATCACCTACAACAGCAACAGCAACA

CCAGCGAGAACAACATGACCATCATCGAGAGCAACTATATATCCGTGCGCGAGGAATATCCCGATATAGA

TAGTGAGGTGCGCGCCATATTGCTGAGCCACGCCCAGAATGGAATCACGATATCGAGCATCAAGAGTGAA

TATCGAAAACTGACGGGCAATCCATTTCCACTGCACGACAACGTGACGGATTTCCTGCTGACCATTCCCA

ATGTGACCGCTGAGTGCAGCGAGTCCGGTAAGCGGATCTTCAACCTGAAAGCGAGCCTGAAGAACGGTCA

CCTCCTGGATATGGTGCTCAACCAGAAAGAGCGCACCAGCGACTACAGCAGCGGAGCTCCGTCCCTGGAG

AACATACCACGAGCACCTCCACGCTACTGGAAGAATCCCTTCAAACGGAGGGCTCTGTCCCAGCTGAACA

CCAGCCCGAGGACCGTGCCCAAGATAACGGATGAAAAGACCAAGGATATCGCCACCAGGCCGGTTTCGCT

GCATCAAATGGCCAATGAGGCAGCGGAGTCGAACTGGTGCTACCAGGATAATTGGAAGCATCTCAACAAT

TTCTACCAGCAAGCCAGCGTAAATGCGCCAAAAATGCCAGTACCCATCAACATCTACAGCCCCGATGCCC

CAGAGGAACCAATCAATTTGGCTCCACCTGGGCATCAGCCAAGCTGCAGAACCCAAAGCCAGAAAACCGA

ACCGACTGAAAACCGCCATTTGGGCATCTTTGTGCATCCATTTAACGGCATGAACATAATGAAGAGACGC

CACGAAATGACGCCCACGCCAACGATTTTAACCAGTGGAACCTACAACGATTCTCTGCTGACGATTAACT

CGGATTACGATGCCTATCTGCTGGACTTTCCGCTTATGGGCGATGATTTTATGCTATATCTCGCCCGAAT

GGAGCTAAAATGCCGATTTAGGCGTCACGAACGCGTCCTGCAGTCAGGACTTTGTGTATCCGGACTGACG

ATCAATGGCGCCCGAAATCGTTTAAAAAGAGTCCAATTACCCGAGGGTACTCAGATCATCGTCAATATCG

GATCGGTGGACATTATGCGCGGCAAGCCTTTGGTTCAGATCGAGCACGATTTTCGGCTACTGATCAAGGA

GATGCACAATATGCGATTGGTGCCGATTCTAACAAATCTTGCACCGCTGGGCAACTATTGTCACGATAAG

GTATTATGTGACAAAATCTACCGATTCAACAAGTTTATCCGAAGCGAATGCTGTCACCTAAAGGTCATCG

ACATACACTCCTGTCTAATCAACGAAAGGGGCGTGGTGCGATTTGATTGCTTTCAGGCCTCACCACGCCA

AGTCACCGGTTCCAAGGAACCCTATCTGTTCTGGAACAAAATCGGTCGGCAGCGCGTACTGCAAGTTATT

GAAACGAGTCTGGAGTATTAAGTTGGGTTCTTAATCAAGATACATATATGCAAATTTTGACTGGGCTGGC

ACCGGAACCGACAAAATAAGAACTTTTTGATGATTTTACGATTTACGCTGATGGATCGCTGCTTTTACGG

AATTCGCTTAGTTTTAATATGTTTTATATGTAGTATGTTCTCTGTCTTTGTTTATTTATATGTTCGTGCA

CTTGTCCTAGTCCATTATTGTATATTATTGTGTGTTTTGTGTTCTATGTTAGATTTAAACTTCTCAATTT

TTCGCTGTCTGTGATTTGTTTTGCCAATGCCATTGATTTTCTGCACACTTTTTGCTGCTATCCCAAAAGC

TGTGTAAAATAATCAAATGCAAAATAAGCGCAAGCAGCTGAAAACTTCTCTTCAAACTTTTCCGCTTTTC

CCAAAACCATTTTGCTTTGAAATCTGTTTTTACCAAATCAGATTAAACTGCAAAATGGAACTTAAATGCA

AATCATTGCAATGCTTATAAACTGTTTTTTGTTCTATATACTTTTGTGTGGGTCAAAATTCGGCATGCTC

CTGTATCACACAACCTGCCACTTGCCCTTAAAAAGAAGGGCGCAGTGGGCGTGGTACGTATACATATGAG

CCATGCTGCATTTTGGCCGTAATGAAAATGCACTGCTTTACTTGGAAAATTCGCTTGCACAAAATCAACG

CCGCGGCTGATTTATTATTGATGTGCTCAAGCAAATTCAAGTGAAGCATTTGCGCGATTTTCGTCTTTCT

GTTTCCGTTTGCAAAAAAGTTTATAAAATGCTTACACTCTGCTGCAGACACGCCAACCGGAAGTGCGCAC

TAAGCGCTTGTTTGTAGCACAGTGTAGAATTCTGGCGTAATTTACAGCTCTACTTTAAAGTCTTCTAGAT

AGCTATCTACTATTTATAAACTTATTTATTGTCTTGAATGTATGTTAATTGTATGTATTGATGGTGATCA

CGTTTTTTTTGTCCTATAACAAGCTGCAATGTAAAATCCAAAAAAAAAATGAAAAAATAAAATAAAAGG

# Technical Hints and Details for AIModules

## Matrix Generation

TFs bind to TFBSs and therefore play a major role in gene expression. They bind promoters and enhancers and can recruit other factors or RNA-Polymerase; they can also reduce gene transcription by binding to repressors. TFs bind specifically to DNA sequences with the highest similarity to the consensus sequence. Identifying TFBSs suggest that transcription is regulated by the corresponding TF. This task of finding those sites is performed *in silico* by using a sort of matrix.

A position weight matrix (PWM) is a form of representing a consensus sequence while also considering the variability of nucleotides at each position (42) (43). For generating such a PWM, functional related sequences are aligned (Table S 18) and the occurrences of each nucleotide in each position are counted (44) (45). In this first step you'll get the position frequency matrix (PFM) with the absolute frequencies of each nucleotide (46) (47).

Table S 18 **Sequence alignment.**

| Sequenz 1 | GAGGTAAAC |
| --- | --- |
| Sequenz 2 | TCCGTAAGT |
| Sequenz 3 | CAGGTTGGA |
| Sequenz 4 | ACAGTCAGT |
| Sequenz 5 | TAGGTCATT |
| Sequenz 6 | TAGGTACTG |
| Sequenz 7 | ATGGTAACT |
| Sequenz 8 | CAGGTATAC |
| Sequenz 9 | TGTGTGAGT |
| Sequenz 10 | AAGGTAAGT |

Position Frequency Matrix (PFM)

$$M=\begin{matrix} A \\ C \\ G \\ T \end{matrix}\left[ \begin{matrix} 3 & 6 & 1 & 0 & 0 & 6 & 7 & 2 & 1 \\ 2 & 2 & 1 & 0 & 0 & 2 & 1 & 1 & 2 \\ 1 & 1 & 7 & 10 & 0 & 1 & 1 & 5 & 1 \\ 4 & 1 & 1 & 0 & 10 & 1 & 1 & 2 & 6 \end{matrix} \right]$$

The PFM can be converted into a position probability matrix (PPM) where the relative frequencies or probabilities are depicted. For that each position in the PFM is divided by the total amount of sequences, or by using the equation from below. Each element in this matrix can be seen as the probability of a nucleotide being found at a specific position in a functional site.

Equation for calculating from a PFM a position probability matrix (PPM)$\begin{aligned} M_{i,j}=\frac{1}{n}\sum_{k=1}^{n} I_{i}\left( s_{k,j} \right) \\ with s_{k}=s_{k1},\cdots,s_{kl} \\ s_{k,j} an Element of\{A,C,G,T\} \\ i=A,C,G,T \\ j=1,\cdots,l \\ The indicator function \\ I_{i}\left( q \right)=\{\begin{matrix} 1, if i=q \\ 0,else \end{matrix}\} \end{aligned}$

The PPM is displayed below.

Position Probability Matrix (PPM)

$$M=\begin{matrix} A \\ C \\ G \\ T \end{matrix}\left[ \begin{matrix} 0.3 & 0.6 & 0.1 & 0.0 & 0.0 & 0.6 & 0.7 & 0.2 & 0.1 \\ 0.2 & 0.2 & 0.1 & 0.0 & 0.0 & 0.2 & 0.1 & 0.1 & 0.2 \\ 0.1 & 0.1 & 0.7 & 1.0 & 0.0 & 0.1 & 0.1 & 0.5 & 0.1 \\ 0.4 & 0.1 & 0.1 & 0.0 & 1.0 & 0.1 & 0.1 & 0.2 & 0.6 \end{matrix} \right]$$

The probability of finding a functional site with this PPM of the length nine on a sequence is the product of each probability from the matrix corresponding the nucleotides on the sequence. The ratio between the probability on a real sequence and a random sequence is called the likelihood ratio, the logarithm thereof the log likelihood ratio.

The log likelihood ratio can also be computed and put into a position weight matrix (PWM). PWMs are represented as log likelihoods in the form that the probabilities at each position in the matrix are divided by the background model and the logarithm to the base two is applied to the result of the division. Therefore, the elements of the PPM are transformed with the equation from below.

Equation for calculating from a PPM a position weight matrix (PWM) $\begin{aligned} M_{i,j}={log}_{2}\left( {M_{i,j}}/{p_{i}} \right) \\ with firstM_{i,j}as the resulting log likelihood of the PWM of nucleotide i at position j \\ second M_{i,j}as the probability of the PPM of nucleotide i atposition j \\ p_{i} as the background model for nucleotide i \left( assuming a uniform distribution: p=0,25 \right) \end{aligned}$

The result is the PWM (see below).

Position Weight Matrix (PWM)

$$M=\begin{matrix} A \\ C \\ G \\ T \end{matrix}\left[ \begin{matrix} 0.26 & 1.26 & -1.32 & -\infty& -\infty& 1.26 & 1.49 & -0.32 & -1.32 \\ -0.32 & -0.32 & -1.32 & -\infty& -\infty& -0.32 & -1.32 & -1.32 & -0.32 \\ -1.32 & -1.32 & 1.49 & 2.00 & -\infty& -1.32 & -1.32 & 1.00 & -1.32 \\ 0.68 & -1.32 & -1.32 & -\infty& 2.00 & -1.32 & -1.32 & -0.32 & 1.26 \end{matrix} \right]$$

If the dataset is small -∞ can occur as a result. To avoid this, pseudocounts can be added. When scanning a sequence with a PWM for functional sites the log likelihoods can be summed up, rather than multiplied when using a PPM. The PWM considers the frequencies of each base and the information content compared to a random sequence, hence reducing entropy.

Each position in a PWM (and PPM) is thought to be statistically independent from all other positions. The resulting sequence score shows the tendency of the site to be a functional site. Since a log likelihood score is used: (*1*) If the score is less than zero, then the site is more likely a result of a random event, (*2*) if it is greater than zero, then the site is more likely to be a functional one, (*3*) if it is equal to zero, than the site's probability to be functional or random is equal.

Using PFMs *AIModules* can predict TFBSs and present them in a graphical form. The PFMs are converted into PWMs during the calculation on the server. Furthermore, the module search functionality can be activated to find putative modules on two or more sequences.

## Mathematical Consideration

A matching unambiguous base contributes to the score by the value of two, i.e. that a matrix of six base pairs or length would have a max score of 6*2 = 12 (=Lm). La represents the minimum log likelihood ratio or smoothed log likelihood ratio score. As some PWMs have a low number of sequences as a basis these can be improved by smoothing the estimates, i.e. by adding a small number of pseudocounts to each position in proportion of a background model. Since the JASPAR (48) matrices with less than 20 aligned sequences are not included in this software setup, the smoothing of estimates does not play a crucial role and is by default one, i.e. 0.25 for each nucleotide. Lm represents the maximum possible score of a PWM. Therefore, it represents the consensus sequence. Ld represents the maximum log likelihood deficit (Lm-La) and is used to filter the results as the upper threshold. It should be noted that La and Ld can produce conflicting results as a higher Ld allows for longer PWMs that have poorer scores and a large La will favor longer and prevent short PWMs. For default scoring we use an La of 7 and Ld of 8. These values can be changed to limit the result of the TFBSs or found modules. The result always represents the (+) and (-) strand. Furthermore, the rendering of the result is done on the client side so it is possible that this step takes a long time. If it takes too long, then the user may decrease the number of input sequences or matrices and increase La and/or decrease Ld.

The putative binding sites include false negatives and false positives. These can be reduced in number by using short sequences or fewer matrices or long matrices with many aligned sequences or a combination of the mentioned hints. Furthermore, setting the La and Ld parameters more strictly can help filter out false negatives and false positives. The use of a background model which is found in the backend of AIModules is second in place when it comes to increasing the value of the predictions. That is, CG poor input sequences with a uniform background model or a slightly AT rich background model do not produce more reliable results, because AT-rich hits get decreased in significance and GC rich hits increased.

It is better to compare the distribution of the scores to the score distribution of a selected test set specifically assembled for each analyzed promoter. This set should reflect the base content of the input sequences (49). As far as we know this has not yet been implemented in any solution.

The log likelihood ratio score is the sum of all the bases from a PWM, where each base can contribute up to 2 bits. When every base contributes these 2 bits of information, you will get the maximum log likelihood ratio score (Lm) or the consensus sequence (19) (20) (21) (50).

The self-information of an element in a PWM is

$$I\left( i \right)=-p_{i,j}*{log}_{2}\left( p_{i,j} \right)$$

and the Information Content (IC) of a PWM

$$I\left( i \right)=-\sum p_{i,j}*{log}_{2}\left( p_{i,j} \right)$$

and with the background frequency the IC is

$$I\left( i \right)=-\sum p_{i,j}*{log}_{2}\left( {p_{i,j}}/{p_{j}} \right)$$

The IC measures the level of conservation which begins from zero (no conservation of position) and ends with two (strictly conserved position). For completely conserved positions the IC is two bits. To get the total IC of the whole PWM the IC of each position has to be summed up. The next occurrence of this PWM appearing next to a found hit is 2^IC^. That means that longer and stronger PWMs with a high La produce more reliable matches. A perfectly conserved 8-mer would therefore have an IC of 8*2=16.

The backend calculates the score via a smoothed log likelihood ratio score (19) (21).

## Using Docker containers

As the backend code is not optimized for data parallelism, we sought a way to handle future high loads on the system. Therefore, we prepared Docker swarm to increase the number of instances per layer as the load increases (see Figure S 21). The Docker swarm operates on nodes. These nodes can be physical or virtual servers, i.e. the load of the containers can be shared across many systems.


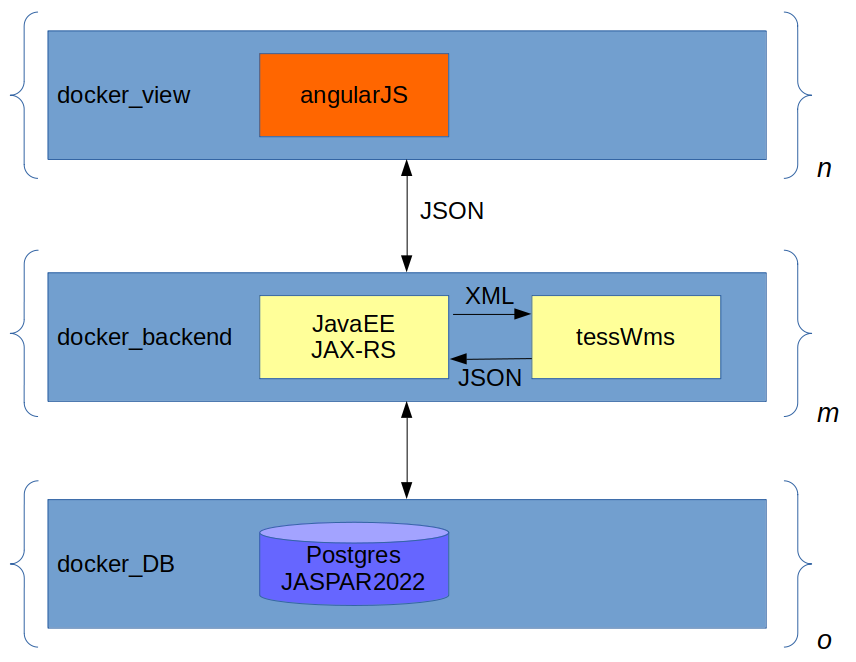


Figure S 21 **Architecture of AIModules when Docker swarm is used.** Each layer has an image template that can be used to create N containers.

## Text field regex Warnings

When the text fields are empty the warnings are:


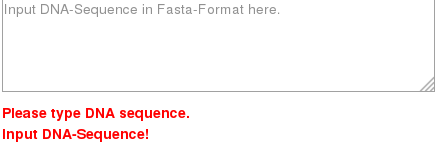


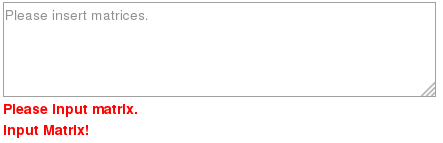


When regex validation fails:


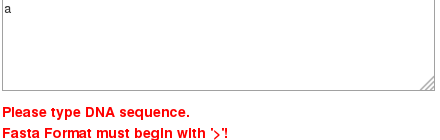


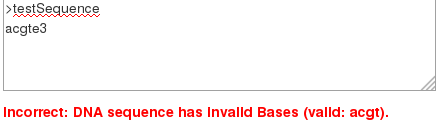


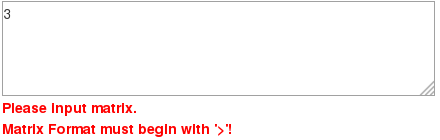


When all the input is correct the user will see this remark:


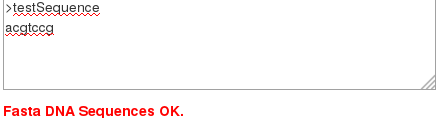


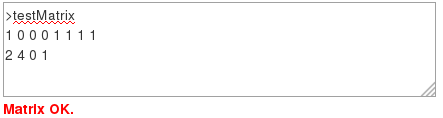


## Development

We used a three-layered architecture where the front-end is developed using angularJS and yeoman with grunt and bower. The back-end is implemented with Eclipse Mars and jdk_1.8.0_74. The executable for searching TFBS (tessWms) is called from the back-end. JAXRS is used for the REST-Services and EclipseLink for the connection to the database. Storage of the matrices is done in a postgres database.

## Build and Deploy

The sources of the tool are published via the URL depicted in the paper under the section *Full availability of data and program.* The front-end can be found in the folder *Sources/frontend*, the back-end in the folder *Sources/backend* and the database in the folder *Sources/postgres*.

The front-end is built with yeoman (<https://github.com/yeoman/generator-angular>, accessed June 13, 2021). For that, change the URL for the back-end first. This is done in the file *tools.js*. The line for that reads:

*$scope.callURL = 'add URL of backend';*

After adding the back-end URL the front-end is built by executing *grunt build* or *grunt build --force*. The resources that were built can be deployed on a webserver.

The back-end project is located in the folder *pwm_rest* and can be loaded into *Eclipse* (https://www.eclipse.org/downloads/, accessed June 13, 2021). Then the URL and port of the postgres installation has to be changed as well as the credentials to this database. This is done in the file *persistence.xml*:

*<property name="javax.persistence.jdbc.url" value="jdbc:postgresql://localhost:5432/docker" />*

*<property name="javax.persistence.jdbc.user" value="user" />*

*<property name="javax.persistence.jdbc.password" value="password" />*

The project can then be build using *Maven* (https://maven.apache.org/guides/getting-started/maven-in-five-minutes.html, accessed June 13, 2021). This generates a *war* archive which can be deployed on a *Tomcat* (http://tomcat.apache.org/, accessed June 13, 2021) application server. When the application server is started the binary file located under *api/WEB-INF/classes/Executable/tessWMS* has to be made executable via the command:

*chmod u+x tessWMS*

The *postgres* (https://www.postgresql.org/, accessed June 13. 2021) database has to be installed first. Then the database may be populated with the provided database backup or the SQL scripts. This will fill the database with the matrices from JASPAR 2022.

Furthermore, this solution can be started as individual *docker* (https://www.docker.com/, accessed June 13, 2021) containers or a *docker swarm* (https://docs.docker.com/engine/swarm/, accessed June 13, 2021). For that, the images first have to be built. First, the updated build from the front-end may be copied into the folder *docker/container/frontend/dist* and then the command to build the docker image is executed:

*docker build -t scorepwm_frontend*

For the back-end, change into the folder *docker/container/backend*. Here the *war* archive may be replaced to point to the postgres database. Then this command will build the image:

*docker build -t scorepwm_backend*

The image for the database can be built in the folder *docker/container/postgres-db* by executing the command:

*docker build -t eg_postgresql*

When all Docker images are built, they can be started individually by executing the commands:

*docker run -v /tmp:/tmp -d -p 80:80 scorepwm_frontend*

*docker run -v /tmp:/tmp -v /tmp/tomcat-logs/:/usr/share/tomcat8/logs/ -v /tmp/tomcat-conf/:/usr/share/tomcat8/conf/ -d -p 8080:8080 scorepwm_backend*

*docker run -d eg_postgresql*

The containers can also be started in a swarm. For that, the swarm must be initialized:

*docker swarm init*

To populate the swarm, this command must be executed in the folder *Sources/docker*:

*docker stack deploy -c docker-compose.yml modules*

To test if the layers are executed and running, go to *http://localhost* for the front-end, to *http://localhost/8080/api/areyoualive* for the back-end and connect to the database with *pg-admin* (https://www.pgadmin.org/, accessed June 13, 2021).

# References

1. Genomatix. [Online] [Cited: July 30, 2021.] http://www.genomatix.de/.

2. Download TESS. [Online] [Zitat vom: 06. April 2022.] https://www.cbil.upenn.edu/downloads/TESS/.

3. Kenneth, D., Vishal, R. P., Paul, R., Xiaohui, X., Pierre, B. MotifMap: integrative genome-wide maps of regulatory motif sites for model species. *BMC Bioinformatics.* 2011, Vol. 12, p. 495. doi:10.1186/1471-2105-12-495.

4. Xie, X., Rigor, P., Baldi, P. MotifMap: a human genome-wide map of candidate regulatory motif sites. *Bioinformatics.* 2009, Vol. 25, pp. 167 - 174.

5. Messeguer, X. et al. PROMO: detection of known transcription regulatory elements using species-tailored searches. *Bioinformatics.* 2002, pp. 333-334.

6. Farré, D. et al. Identification of patterns in biological sequences at the ALGGEN server: PROMO and MALGEN. *Nucleic Acids Res.* 2003, 31(13), pp. 3651-3. http://alggen.lsi.upc.es/cgi-bin/promo_v3/promo/promoinit.cgi?dirDB=TF_8.3.

7. Wrzodek, C. et al. ModuleMaster: A new tool to decipher transcriptional regulatory networks. *Biosystems.* 2010, 99(1), pp. 79-81.

8. Münch, R. et al. PRODORIC: prokaryotic database of gene regulation. *Nucleic Acids Res.* 2003, 31(1), pp. 266-9.

9. —. Virtual Footprint and PRODORIC: an integrative framework for regulon prediction in prokaryotes. *Bioinformatics.* 2005, Vol. 21, pp. 4187-4189.

10. Solovyev, V. V., Shahmuradov, I. A. PromH: Promoters identification using orthologous genomic sequences. *Nucleic Acids Res.* 2003, Vol. 31(13), pp. 3540-3545.

11. Softberry NSITE. [Online] [Zitat vom: 11. May 2020.] http://www.softberry.com/berry.phtml?topic=nsite&group=programs&subgroup=promoter.

12. Solovyev, V. V., Shahmuradov, I. A., Salamov, A. A. Identification of promoter regions and regulatory sites. *Methods Mol Biol.* 2010, 674, pp. 57-83.

13. Shahmuradov, I., Solovyev, V. Nsite, NsiteH and NsiteM Computer Tools for Studying Transcription Regulatory Elements. *Bioinformatics.* 2015, 31(21), pp. 3544-5.

14. Softberry NSITEH. [Online] [Zitat vom: 5. Nov 2020.] http://www.softberry.com/berry.phtml?topic=nsiteh&group=programs&subgroup=promoter.

15. Softberry NSITEM. [Online] [Zitat vom: 5. Nov 2020.] http://www.softberry.com/berry.phtml?topic=nsitem&group=programs&subgroup=promoter.

16. TAIR. [Online] [Cited: July 30, 2021.] https://www.arabidopsis.org/tools/bulk/motiffinder/index.jsp.

17. Chow, C. N. et al. PlantPAN3.0: a new and updated resource for reconstructing transcriptional regulatory networks from ChIP-seq experiments in plants. *Nucleic Acids Res.* 2019, pp. 1154–1160.

18. PlantPan 3.0. [Online] [Zitat vom: 6. Nov 2020.] http://plantpan.itps.ncku.edu.tw/gene_group.php?#multipromoters.

19. Schug, J. Using TESS to predict transcription factor binding sites in DNA sequence. *Bioinformatics.* 2008, Chapter 2:Unit 2.6.

20. Schug, Jo. Tess. [Online] [Cited: July 30, 2021.] https://www.cbil.upenn.edu/tess.

21. Overton, G.C., Schug, J. Tess: Transcription element search software on the www. *Laboratory, School of Medicine, University of Pennsylvania.* 1997.

22. Genomatix. [Online] [Cited: Aug 30, 2018.] http://www.genomatix.de/.

23. Kel, A. E. et al. MATCH: A tool for searching transcription factor binding sites in DNA sequences. *Nucleic Acids Res.* 31(13), July 1, 2003, pp. 3576-9. PMID: 12824369; PMCID: PMC169193.

24. Matys, V. et al. TRANSFAC: transcriptional regulation, from patterns to profiles. *Nucleic Acids Res.* 31(1), Jan 1, 2003, pp. 374-8. PMID: 12520026; PMCID: PMC165555.

25. TRANSFAC Match. [Online] [Zitat vom: 7. Nov 2020.] https://portal.genexplain.com/archive/videos/composite_elements_2014_1.mp4.

26. Public Transfac Database. [Online] [Zitat vom: 22. Oct 2020.] http://gene-regulation.com/pub/databases.html.

27. Transfac Profession vs. Public. [Online] [Zitat vom: 12. Nov 2020.] https://portal.genexplain.com/archive/documents/transfac_comparison.pdf.

28. Kreft, Ł. et al. ConTra v3: a tool to identify transcription factor binding sites across species, update 2017. *Nucleic Acids Research.* Volume 45, 3. July 2017, Bd. Issue W1, S. W490–W494. https://doi.org/10.1093/nar/gkx376.

29. —. Website: ConTra v3: a tool to identify transcription factor binding sites across species, update 2017. *ConTra v3.* [Online] [Zitat vom: 4. Oct 2020.] http://bioit2.irc.ugent.be/contra/v3/#/step/1.

30. Weirauch, M. T. et al. Determination and inference of eukaryotic transcription factor sequence specificity. *Cell.* 11. Sep 2014, 158(6), S. 1431-43.

31. CisBP. [Online] [Zitat vom: 06. Nov 2021.] http://cisbp.ccbr.utoronto.ca/TFTools.php.

32. Hume, M. A., Barrera, L. A., Gisselbrecht, S. S., Bulyk, M. L. UniPROBE, update 2015: new tools and content for the online database of protein-binding microarray data on protein-DNA interactions. *Nucleic Acids Research.* 2014.

33. UniPROBE. UniPROBE. [Online] [Zitat vom: 31. Oct 2021.] http://the_brain.bwh.harvard.edu/uniprobe/index.php?ncsg=1.

34. HOCOMOCO. [Online] [Zitat vom: 06. Nov 2021.] https://hocomoco11.autosome.ru.

35. Kulakovskiy, I. V. et al. HOCOMOCO: towards a complete collection of transcription factor binding models for human and mouse via large-scale ChIP-Seq analysis. *Nucl. Acids Res.* 11. Nov 2017, Database issue, S. gkx1106.

36. FlyFactorSurvey. [Online] [Zitat vom: 06. Nov 2021.] https://pgfe.umassmed.edu/ffs/.

37. MEME Suite. [Online] [Zitat vom: 06. Nov 2021.] https://meme-suite.org/meme/tools/meme.

38. Bailey, T. L., Johnson, J., Grant, C. E., Noble, W. S. The MEME Suite. *Nucleic Acids Research.* 2015, 43(W1), S. W39-W49.

39. YeTFaSCo. [Online] [Zitat vom: 06. Nov 2021.] http://yetfasco.ccbr.utoronto.ca/.

40. de Boer, C. G., Hughes, T. R. YeTFaSCo: a database of evaluated yeast transcription factor sequence specificities. *Nucleic Acids Research.* 01. Jan 2012, Bd. Issue D1, Volume 40, S. D169–D179.

41. Chow, C. W., Rincón, M., Davis, R. J. Requirement for transcription factor NFAT in interleukin-2 expression. *Mol Cell Biol.* 19(3), 1999, S. 2300-2307. doi:10.1128/mcb.19.3.2300.

42. Stormo, G. D., Schneider, T. D., Gold, L., Ehrenfeucht, A. Use of the 'Perceptron' algorithm to distinguish translational initiation sites in E. coli. *Nucleic Acids Res.* 1982, 10(9), pp. 2997-3011.

43. Stormo, G. D. Dna binding sites: representation and discovery. *Bioinformatics.* 2000, 16(1), S. 16-23.

44. Chen, X., Guo, L., Fan, Z., Jiang, T. Learning position weight matrices from sequence and expression data. *Comput Syst Bioinformatics Conf.* 2007, 6, S. 249-60.

45. Gribskov, M., McLachlan, A. D., Eisenberg, D. Profile analysis: detection of distantly related proteins. *Proc Natl Acad Sci U S A.* 1987, 84(13), S. 4355-8.

46. Guigo, R. An introduction to position specific scoring matrices. 2003.

47. Wikipedia. *Position weight matrix.* [Online] [Cited: July 30, 2021.] https://en.wikipedia.org/wiki/Position_weight_matrix.

48. Castro-Mondragon, J.A., et al. JASPAR 2022: the 9th release of the open-access database of transcription factor binding profiles. *Nucleic Acids Res.* gkab1113, 2021.

49. DaveTang. [Online] 2019. [Cited: Dec 23, 2019.] https://davetang.org/muse/2013/10/02/transcription-factor-binding-site-prediction/.

50. Wikipedia. Position weight matrix. [Online] 2018. [Cited: Aug 31, 2018.] https://en.wikipedia.org/wiki/Position_weight_matrix.

51. Chen, X., Guo, L., Fan, Z., Jiang, T. Learning position weight matricesfrom sequence and expression data. *Comput Syst Bioinformatics Conf.* 2007, 6, pp. 249-60.

52. Fornes, O. et al. JASPAR 2020: update of the open-access database of transcription factor binding profiles. *Nucleic Acids Research.* Volume 48, Januar 8, 2020, Vol. Issue D1, pp. D87–D92.

53. Stormo, G. D., Schneider, T. D., Gold, L., Ehrenfeucht, A. Use of the 'Perceptron' algorithm to distinguish translational initiation sites in E. coli. *Nucleic Acids Research.* 1982, 10(9), pp. 2997-3011.

54. Xie, B., Jankovic, B. R., Bajic, V. B., Song, L., Gao, X. Poly(A) motif prediction using spectral latent features from human DNA sequences. *Bioinformatics.* 29(13), 1. July 2013, S. i316-25. doi: 10.1093/bioinformatics/btt218.
