# Supplementary material for: Motif and conserved module analysis in DNA (promoters, enhancers) and RNA (lncRNA, mRNA) using AlModules
Source: Sci Rep. 2022 Oct 20;12:17588. doi: 10.1038/s41598-022-21732-0 (PMC9584888; doi:10.1038/s41598-022-21732-0)
Supplement: Supplementary file 3 — Supplementary Information 3. [file 41598_2022_21732_MOESM3_ESM.docx]

# Tutorial

**Motif and conserved module analysis in DNA (promotors, enhancers) and RNA (lncRNA, mRNA) using AlModules**

Muharrem Aydinli^1^*, Chunguang Liang^1^*, Thomas Dandekar^1,§^

^1^ Dept. of Bioinformatics, Biocenter, Am Hubland, D-97074 University of Würzburg

Independent 2^nd^ file: The Evaluation file evaluates and compares AIModules to alternative software and programs.

Independent 3^rd^ file:

- Data-Supplements.zip (the detailed evaluation results on finding conserved modules and TFBS are given in comparing AIModules_vs_Genomatix)

# How to access the web application

The web application AIModules can be accessed via the Internet. For that, our group provides two secure servers – one main server and one as backup. However, we invite the readers to use either server. The URLs are

- <https://bioinfo-wuerz.de/aimodules>
- <https://aimodules.heinzelab.de>

The software is also available from our software home page (via redirect)

- <https://www.biozentrum.uni-wuerzburg.de/bioinfo/computing/aimodules>

The User Interface is designed to support the user, so that an easy and fast start is possible: The user sequence has to be given in FASTA format for the software to start.

First, the presence of only nucleotide characters is tested. Only then the submit button appears. Click to start the search.

If not specified in the menu below, the application uses default parameters, i.e. the JASPAR transcription factor database, version 2022 (all matrices and is doing a TFBS search). Progress is indicated by a rotating wheel close by. Alternatively, the user can first hit any of the demo buttons to see the results AIModules delivers for different use cases.

After module calculation, the result for the module search is depicted visually and can be downloaded as a detailed Excel file (see Figure S 9 and text following that figure). An svg file delivers a picture of all transcription factors found in the search.

Moreover, using “mouse over,” the user sees the matrices of the transcription factors.

Looking directly at the annotated promotor region, the mouse over delivers the information from where to where the transcription factor binding region was found.

If the reader (e.g. a company) wants to install the web application on their own servers or on a laptop, they may do so with the provided source code (see main manuscript). The minimum system requirements should include 10 GB of hard disk space, a modern processor and at least 2 GB of Ram.

# Use Cases: What can I do with AI-Modules?

The web application offers useful functionalities for biomedical, bioinformatics or molecular biology questions.

In order to execute a new analysis, the web application has to be reloaded first. For that, the red button “Restart” may be used, which appears after each analysis.

The first step of the calculations is very fast because it is executed on the server. The next two steps “module search” and “rendering” must be carried out within the browser. Currently, the complex module analysis consisting of many DNA stretches relies on the resources of the client PC. This means that these two steps may take minutes for a final result and the browser may seem unresponsive, accompanied by a warning from the browser to “wait” or “cancel” the browser tab. If the user waits, the result will appear.

The calculations on the client PC rely heavily on memory. Therefore, the PC should be equipped with at least 8 GB of Ram (better 16 GB), if complex analyses are executed.

If low Ram is preventing the result to appear, it is a reasonable idea to start analyses with a low number of DNA sequences, or only search for few manually selected transcription factors, or deactivate the module search. By doing so, the user can easily select the TFs of interest and use those for the following module search.

This approach, to separate the calculation steps between the server and the client PC, effectively allow our servers to serve many more users at the same time. However, to increase the user experience, we will move all the calculations onto the servers in a future step.

## Use Case 1: Promoter motifs: TFBS and combinations (modules)

### Use Case 1.1: Identify for a specific TF the specific TFBS in a promoter region comparing several promoter regions

AIModules allows in its basic functionality the search for transcription factor binding sites. For that, the user inserts sequences and selects predefined or manually selected matrices. The result is then rendered as seen in Figure S 1, where the black lines represent the inserted sequences of interleukin-10 promoters from different species with colored binding sites. These are located above those lines for the *for* strand and below the line for the *rev* strand. The checkboxes in the lower half of Figure S 1 correlate with the binding sites in the above half, so that the user may only select binding sites of interest.


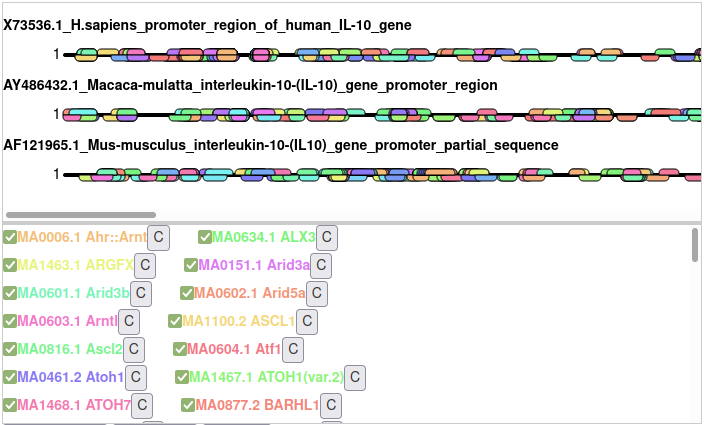


Figure S 1 Transcription factor binding site search

### Use case 1.1b: Adapt the TF motif as more variation is recognized by more experimental data, comparing several binding site data sets

AIModules allows insertion of user selected matrices. As new motifs and motif combinations are discovered by e.g. the ENCODE consortium (1; 2), they can be used within AIModules to conduct up-to-date analyses.

### Use case 1.2: Identify conserved TF binding sites comparing several genes in a gene family either shared between species or occurring within a species

Since AIModules offers flexibility towards the user, both sequences and matrices may be manually selected and used for analyses. Therefore, carefully selected related sequences can be searched for conserved TFBSs.

### Use case 1.3: Identify a conserved promoter **module** in a gene family comparing several promoter regions

One of the main functionalities of AIModules is the search for conserved modules (coupled motifs). As depicted in Figure S 2 we searched for modules in different interleukins from *Mus musculus*. The found modules show very well that similar modules are shared among all sequences.


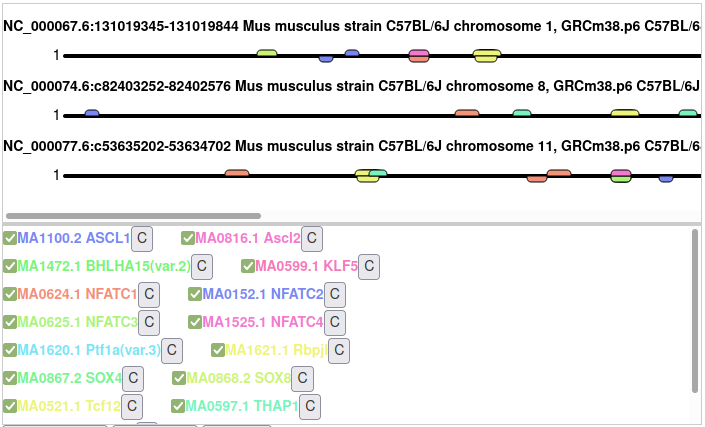


Figure S 2 Result of a module search

### Use case 1.4: Find a cell-type specific **module**, e.g. a liver-specific module

Modules are often cell-type specific. This means that our general approach of TFBS and module searches allows discovery of cell-type specific modules, once the relevant matrices are provided. Examples are liver-specific transcription factor combinations (including liver-specific binding factor 1 in different module combinations) or neuronal cell-specific modules (which then include transcription factors and TFBS which are specific for neurons).

### Use case 1.5: Find a functional **module**, e.g. an immunoglobulin module

Figure S 1 illustrates very nicely the use case for TFBS searches, where TFs for interleukin-10 from different species are calculated. With the appropriate matrices, our solution facilitates finding functional modules. This is depicted for immunoglobulin modules in Figure S 3. As described above, the top half of that figure shows TFBSs in sense and antisense direction. The colors of each TFBS correlates with the bottom half of the figure, where the user may select or deselect TFs to assemble a custom TF-profile.


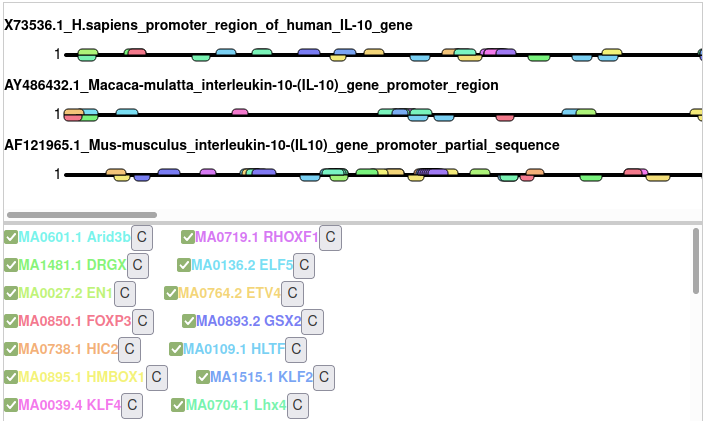


Figure S 3 Module search of interleukin-10 from different species

### Use case 1.6: Promoter Module search

Modules are transcription factors that share a specific distance to each other and are conserved amongst different species. Our solution supports this kind of study very well, as it offers custom searches. For that, the reader may select their own DNA sequences and start the search with predefined matrices. In addition, the web application also supports user defined matrices.

The section Hands-on-Tutorial on the User Interface describes in more detail the sections of the web application. We invite the reader to read about the procedure and correct handling of it there.

Figure S 4 shows a result of our web application. The figure depicts a module search for three different cathepsins. The top section presents the module matches for three DNA sequences. The matches are depicted in color for both sense and antisense. The colors of the DNA matches correlate with the lower part of the figure. Here, the user finds the name of the transcription factor and may choose to show or deselect specific TFs. In that way, the upper part of the figure gets updated to emphasize a specific module.


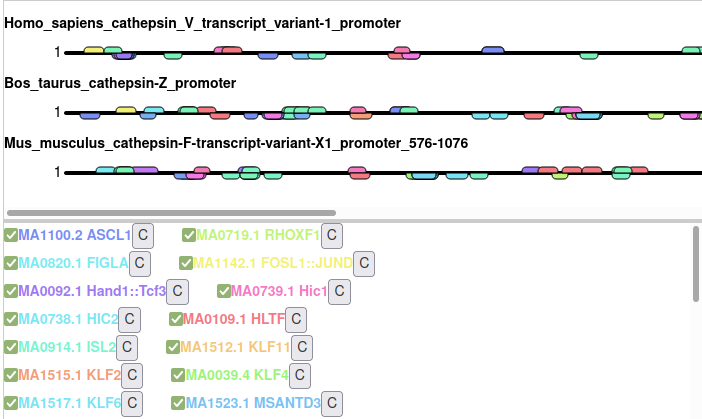


Figure S 4 Module search of cathepsins

## Use Case 2 Searching for RNA motifs

Our solution not only finds modules, but also support the search for RNA motifs such as poly adenylation site motifs or splice sites. We wanted to offer a web application with consistent behavior and usage. Therefore, scanning sequences for RNA motifs is similar to scanning for modules. For further advice we recommend further readings in section Hands-on-Tutorial on the User Interface.

### Use Case 2.1: Searching for a polyadenylation site motif in the 3´region of an mRNA

Figure S 5 depicts such a search from a matrix that was generated from twelve consensus sequences. The matches are colored and positioned above the black sequence line for the sense strand and below the line for the antisense strand.


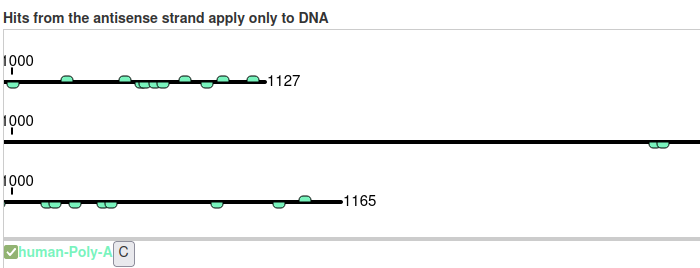


Figure S 5 Searching for a polyadenylation site motif

The [processive](https://en.wikipedia.org/wiki/Processivity) polyadenylation complex in the nucleus of eukaryotes works on products of RNA polymerase II, such as precursor mRNA. Here, a multi-protein complex cleaves the 3′-most part of a newly produced RNA and polyadenylates the end produced by this cleavage. The cleavage is catalyzed by the enzyme CPSF and occurs 10–30 nucleotides downstream of its binding site. This site often has the polyadenylation signal sequence AAUAAA on the RNA, but variants of it exist, which bind more weakly to CPSF. Two other proteins add specificity to the RNA binding: CstF and CFI. CstF binds to a GU-rich region further downstream of CPSF's site. CFI recognizes a third site on the RNA (a set of UGUAA sequences in mammals) and can recruit CPSF even if the AAUAAA sequence is missing. The polyadenylation signal – the sequence motif recognized by the RNA cleavage complex – varies between groups of eukaryotes (3; 4).

Most human polyadenylation sites contain the AAUAAA sequence or the AAUUAA sequence (5) but this sequence is less common in plants and fungi (6).

Hence, looking for such a motif, you can identify where the polyadenylation starts and a simple matrix containing this basic motif is given for the user to allow such predictions.

### Use case 2.2: Identify RNA protein binding region

The sm-site motif occurs in RNAs to direct proteins binding to this motif. In particular, splicing RNAs such as U1, U2, U4, U5 and U6 contain the sm-site motif. This motif contains two purines followed by a poly U stretch, which may contain another nucleotide, followed by another two purines (Table S 1).

Table S 1 snRNP motifs

| **snRNP motifs** |
| --- |
| Gguucugg |
| aguuugugg |
| gauucuuga |
| aguuauuuga |
| aauauuaa |
| aguauuuaa |
| gauuuuuaa |
| ggucuuga |
| aguuguag |
| gauauuugg |
| gauucugg |
| aguuuguuugg |
| aauuuuaa |
| aauuuuuga |
| gauauuugg |

A matrix from the motifs from Table S 1 can be produced within AIModules to be used for splicing site searches.

### Use case 2.3: RNA localization motif. Look for the anterior pole localizing motif in *oskar* mRNA

The localization of mRNA in *D. melanogaster* oocytes is shown in (7). Here the *oskar* mRNA is localized at the posterior pole of the oocyte. The protein IMP (insulin growth factor II mRNA–binding protein) is co-localized and binds to the mRNA *oskar* in the 3’UTR. The binding motif for IMP is the IMP-binding element (IBE), which has the sequence UUUAY. We used both pyrimidine binding sequences UUUAC and UUUAU to generate a matrix within AIModules (Figure S 6). THE IBE motif occurs 13 times in the *oskar* 3’UTR.


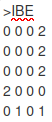


Figure S 6 Matrix for IBE bonding site

We used the *oskar* transcript variant A (NM_169248.4) (see supplemental evaluation for “Used sequences”) for the analysis in AIModules, which produced a fitting result (Figure S 7).


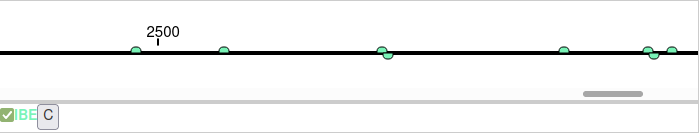


Figure S 7 IBE binding sites in the oskar mRNA

We encourage the reader to visit our web application (<https://bioinfo-wuerz.de/aimodules> or <https://aimodules.heinzelab.de>) and recreate the examples from our tutorial.

## Use case 3: Further DNA motifs

The encyclopedia of DNA elements shows a number of DNA motifs apart from promoter motifs. (1; 2)

Due to the generic nature of AIModules, analyses may incorporate a wide variety of functional genomic elements, including promoters. The prerequisite for this is that the tool is supplied with the appropriate matrices.

### Use Case 3.1: Enhancers

We hope that with our approach we can help to shed light into the realm of enhancers, which needs more tools to understand that subject better.

### Use Case 3.2: Origins of replication

Furthermore, our tool can help elucidate origins of replication. Our solution is not domain specific, so that origins of replication can be searched in Archaea, Bacteria and Eukarya (8).

### Use Case 3.3: Centromeres

The kinetochore assembly and the segregation of chromatids happen at centromeres that contain satellite-rich chromatin domains in mammals. With the consensus sequences of the core centromere and the pericentromeric satellite repeats a matrix can be deduced (9) and used in AIModules for centromere recognition.

### Use Case 3.4: Telomere repeats

The human telomere is guanine rich and consists of repeats of (TTAGGG)_n_ (10) at the end of the chromosomes and the repeats differ among species. With this motif it is an easy task for AIModules to generate a matrix for successful telomere enumeration on DNA sequences.

# Hands-on-Tutorial on User Interface

We tried to keep the User Interface as simple as possible so any new user can easily use AIModules (see Figure S 8 - Figure S 14 in this document). The “Submit” button from Figure S 8 gets visible when the FASTA sequence from the user passes the technical tests which assures that only nucleotides are inserted. After clicking the “Submit” button default parameters are set and “All” matrices from JASPAR 2022 are selected for the analysis. An animation below the button appears that indicates an active search. When it disappears, the result is printed into an svg container which can be downloaded as an svg file. If the user prefers to download an Excel file with detailed information, the application allows this as well. For that, the button “TFBS” from Figure S 12 can be used to open a new window as depicted in Figure S 13, on which the button “Export Result” generates and downloads the Excel file.

For custom searches the user may define parameters (La and Ld) and the matrices as the data source (see Figure S 9) as well, and for module searches may activate the checkbox in Figure S 9. In any case, the result is rendered into the svg container. In the case of module searches, another button “Modules” appears close to the “TFBS” button (as depicted in Figure S 12), which shows detailed information on modules and allows download of this information as an Excel file as well.


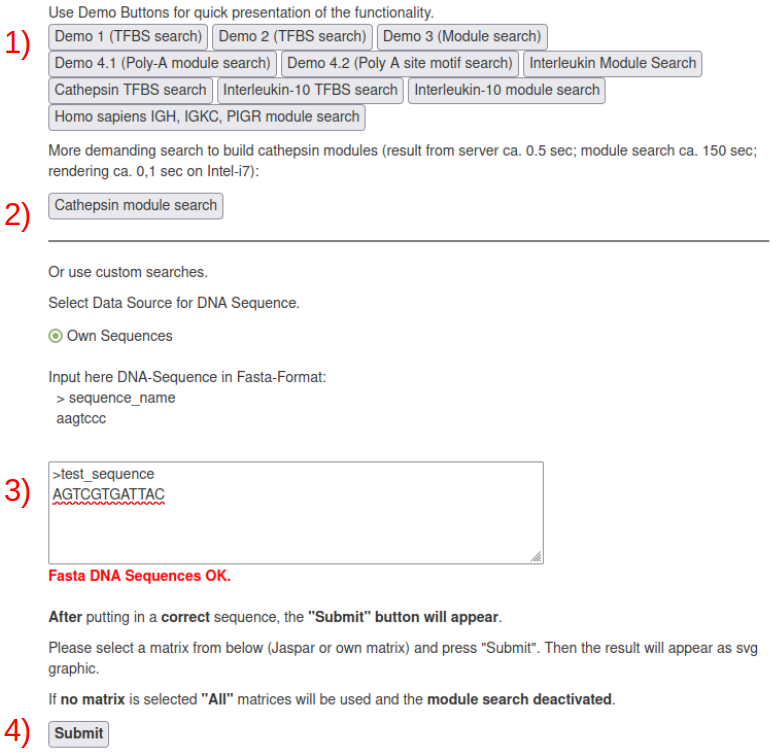


Figure S 8 **AIModules.** The numbers in RED are inserted to ease the description in this paper. 1) Buttons for different analyses. 2) A more demanding analysis: The backend responds with the TFBSs in 0.5 sec, and the client calculates the modules in 150 secs and renders the final result in 0.1 sec. 3) The text field in which the user can input FASTA formatted DNA sequences and also RNA sequences. In the latter case the Uracil (U) is converted into Thymine (T). 4) With a correct sequence in 3) the “Submit” button appears and can be used to start the TFBS analysis with default parameters and all JASPAR 2022 matrices. When the analysis is finished statistical information is shown below the “Submit” button. The button itself gets deactivated. For the next analysis the webpage has to be reloaded or the “RESTART” button at the bottom below the SVG container with the result has to be pressed. If the response takes too long, the request has to be altered in a way to reduce the load for the backend or frontend, e.g. by using fewer sequences or increasing La or decreasing Ld or reducing the number of the matrices. (Please visit <https://bioinfo-wuerz.de/aimodules> or <https://aimodules.heinzelab.de> for the newest version.)


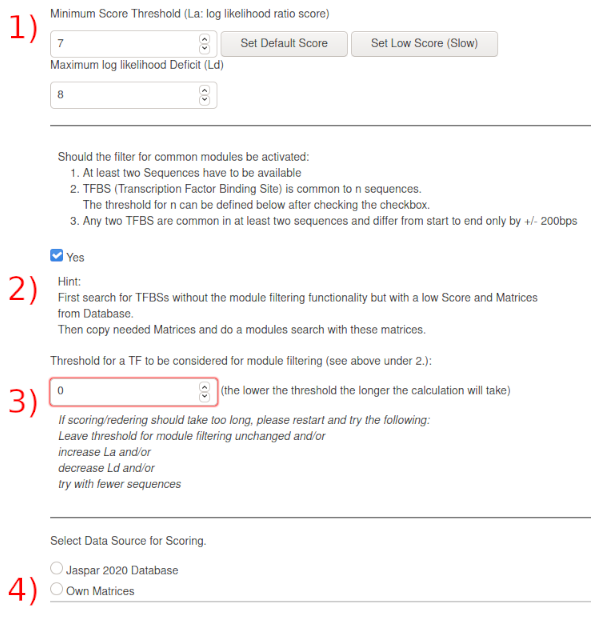


Figure S 9 **AIModules continued.** The numbers in RED are inserted to ease the description in this paper. 1) The maximum log likelihood Deficit defines a threshold below the maximum log likelihood ratio where results are valid. The maximum log likelihood ratio for a matrix is the consensus sequence, where each position can contribute up to 2 bits to the result. 2) This checkbox activates the module filtering function but is optional for a plain search for TFBSs. 3) When the checkbox is checked a stepper is revealed, which shows the number of sequences the user has input. This parameter can be changed and defines in how many of the user input sequences a TF has to be found to be valid for module filtering. 4) Two radio buttons: The upper one allows selection of saved Jaspar Matrices, the lower one activates a text field for the user to input their own matrices in FASTA format. (Please note, that the figure states “Jaspar 2020”. However, the database has been updated to JASPAR 2022 without changes to the website. Please visit <https://bioinfo-wuerz.de/aimodules> or <https://aimodules.heinzelab.de> for the newest version.)

Matrices can either be selected from the database (see Figure S 10) or input manually (see Figure S 11).


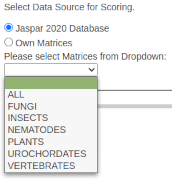


Figure S 10 **Matrices from supported phyla can be chosen from...**(Please note, that the figure states “Jaspar 2020”. However, the database has been updated to JASPAR 2022 without changes to the website. Please visit <https://bioinfo-wuerz.de/aimodules> for the newest version.)


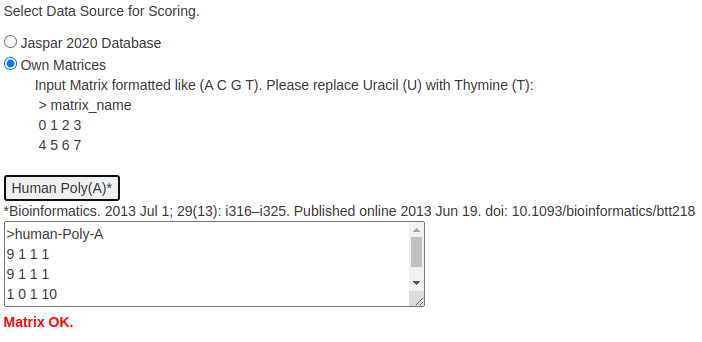


Figure S 11 **...or manually inserted.** Furthermore, a matrix from human poly adenylation site motifs is available for selection.

(Please note, that the figure states “Jaspar 2020”. However, the database has been updated to JASPAR 2022 without changes to the website. Please visit <https://bioinfo-wuerz.de/aimodules> for the newest version.)

When the DNA/RNA sequences and the matrices pass the regular expression search pattern, the *Score* button becomes visible. When it is used the request is send to the backend. The response then is rendered (see Figure S 12).


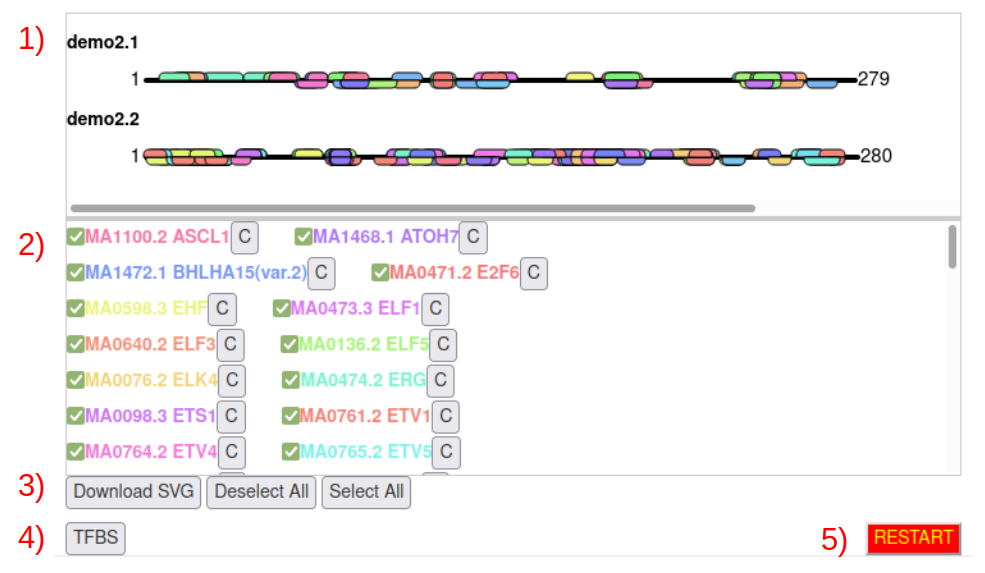


Figure S 12 **Result for TFBS search.** The numbers in RED only ease the description done here and are not shown on the website. Most of the controls on the website have a mouse-over functionality implemented. 1) The result is rendered into a SVG container. The sequence name is depicted. The black line indicates the sequence itself. Above the black line are the TFBS hits for the (+) strand and below for the (-) strand. Each matrix has its own color which corresponds to the list in 2). Here are all the valid results with the colors used in 1). To the right is a button with a C. This button can be used to copy the matrix into the clipboard. Each matrix can be deselected and selected again. This selection has a direct binding to 1). If the user deselects all matrices the resulting picture in 1) will be empty. 1) and 2) can be downloaded as SVG files. For that, the user clicks the Download SVG button. The selection from 2) can be viewed as a table. For that the user clicks the TFBS button (4)). For the next analysis the session has to be restarted (5)).

The timer below the *Score* button becomes active and indicates that the request has been sent. It stops when the response is retrieved, which is then rendered on the client side. Furthermore, module and rendering timers are also visible. We chose client-side rendering to reduce the load on our servers. If rendering is completed the result is shown in an SVG-container. If either the timer takes too long and does not stop, or the rendering takes too long, then the user may send another request with either fewer sequences, or higher La, or lower Ld, or fewer matrices, or any combination of these.

The TFBS button opens another browser instance and shows the selected result as a table (see Figure S 13).


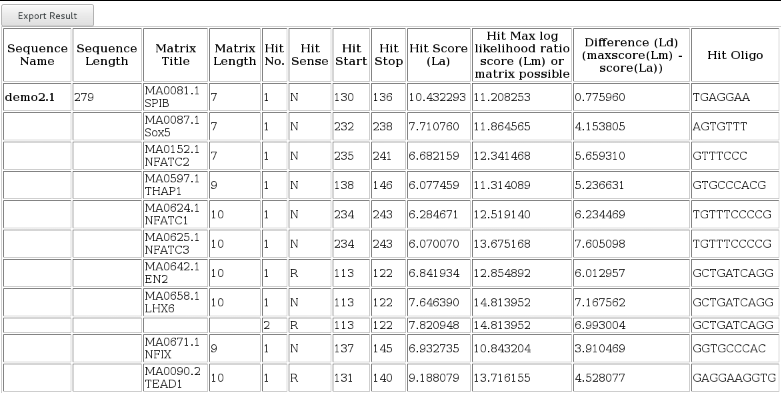


Figure S 13 **The TFBS Result Page.** The table can be downloaded as an Excel file using the Export Result button.

When the initial analysis to find TFBSs is finished the user can venture towards finding modules. For that the matrices of interest can be selected and input into the *Own Matrices* text field (see Figure S 11). For module searches the corresponding checkbox has to be activated. A putative module is an arrangement of two consecutive TFBSs. These have to be *(a)* common to n input sequences (see Figure S 9 2)-3)), *(b)* cannot differ from start to end more than +/- 200 base pairs and *(c)* this arrangement of two TFBSs have to be common to at least two input sequences. When response rendering is completed the user is presented with an extra *Modules* button.

Behind this extra button lies the putative modules depicted as a table. The table can be downloaded as an Excel file using the *Export Result* button as well. Moreover, the user may select and deselect TFBSs to arrange and analyze selected modules.

Furthermore, the web service allows the conversion of putative homologous TFBSs sequences to a matrix format which can be used in the web service itself (see Figure S 14).


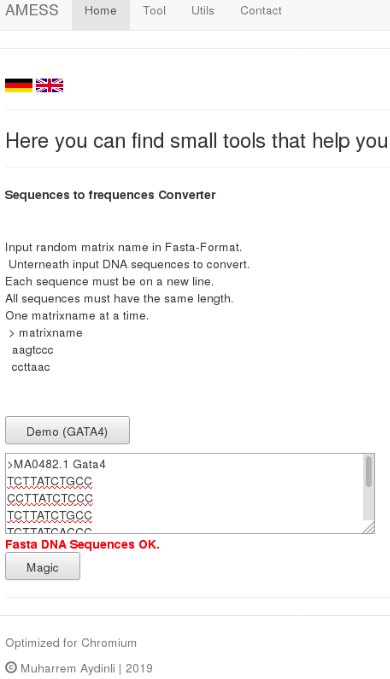


Figure S 14 **Matrix Generator.** The user can input putative homologous TFBSs with the same length. The tool then calculates the matrix to be used in this web service.

The result is a matrix (see Figure S 15) which can be used to find TFBSs or modules (see Figure S 11).


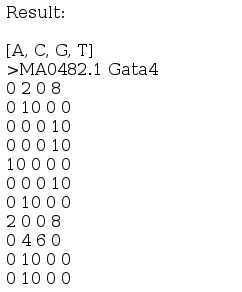


Figure S 15 **Result from Matrix Generator.**

# References

1. **Zhou, B. et al.** Comprehensive epigenomic profiling of human alveolar epithelial differentiation identifies key epigenetic states and transcription factor co-regulatory networks for maintenance of distal lung identity. *BMC Genomics.* Dec 18, 2021, 22(1), p. 906.

2. **Moore, J. E. et al.** Expanded encyclopaedias of DNA elements in the human and mouse genomes. *Nature.* Jul 2020, 583(7818), pp. 699-710.

3. **Ji, G. et al.** Predictive modeling of plant messenger RNA polyadenylation sites. *BMC Bioinformatics.* 2007, 8:43.

4. **Koh, C. H., Wong, L.** Recognition of polyadenylation sites from Arabidopsis genomic sequenses. *In Proceedings of 18th International Conference on Genome Informatics.* 2007, pp. 73-82.

5. **Beaudoing, E. et al.** Patterns of variant polyadenylation signal usage in human genes. *Genome Research.* July 2000, 10 (7), pp. 1001–10.

6. **Shen, Y. et al.** Genome level analysis of rice mRNA 3′-end processing signals and alternative polyadenylation. *Nucleic Acids Research.* May 2008, 36 (9), pp. 3150–61.

7. **Munro, T. P., Kwon, S., Schnapp, B. J., Johnston, B. S.** A repeated IMP-binding motif controls oskar mRNA translation and anchoring independently of Drosophila melanogaster IMP . *J Cell Biol.* Feb 13, 2006, 172(4), pp. 577-588.

8. **Leonhard, A. C., Méchali, M.** DNA replication origins. *Cold Spring Harb Perspect Biol.* Oct 1, 2013, 5(10), p. a010116.

9. **Perea-Resa, C., Blower, M. D.** Centromere Biology: Transcription Goes on Stage. *Mol Cell Biol.* Aug 28, 2018, 38(18), pp. e00263-18.

10. **Meyne, J., Ratliff, R. L., Moyzis, R. K.** Conservation of the human telomere sequence (TTAGGG)n among vertebrates. *Proc Natl Acad Sci U S A.* 1989, 86(18), pp. 7049-7053.

11. **Genomatix.** [Online] [Cited: 08 30, 2018.] http://www.genomatix.de/.

12. **Download TESS. [Online] [Cited: 04 06, 2022.] https://www.cbil.upenn.edu/downloads/TESS/.**
